# Social media users experience more political hostility in less economically equal and less democratic societies

**DOI:** 10.1038/s41562-026-02432-5

**Published:** 2026-04-03

**Authors:** Alexander Bor, Antoine Marie, Lea Pradella, Michael Bang Petersen

**Affiliations:** 1 Democracy Institute, Central European University, Budapest, Hungary; 2 Center for Political Research, Sciences Po, Paris, France; 3 Department of Political Science, Aarhus University, Aarhus, Denmark

**Keywords:** Human behaviour, Politics and international relations

## Abstract

There is widespread concern about the hostility of political discussions on social media, but there is no consensus about the underlying dynamics. In particular, the relationship between online hostility and the broader sociopolitical context has received less attention, in part because of limited research outside Western countries. Here we report results from observational data collected through quota-sampled online surveys in 30 countries across six continents (*N* = 15,202) about experiences of online hostility. Our findings show that people in less democratic and less economically equal countries experience more hostility online. We also found that, in every country, respondents who are hostile online are also hostile offline and that these people score higher in status-seeking motivations. Exploratory analyses suggest that less democratic societies include more status-motivated individuals and young men—groups showing higher hostility on average. Overall, these findings highlight how online political hostility is intertwined with wider societal tensions.

## Abstract (web summary)

Drawing on survey data from 30 countries, Bor et al. find that online political hostility is higher in more unequal and less democratic societies.

## Main

The early days of the Internet were characterized by a sense of techno-optimism. Commentators and researchers hoped that the Internet would become a platform for more equal and inclusive public debates by offering a way to circumvent traditional gatekeepers such as media editors and party officials^1,2^. This optimism extended to autocratic regimes, and the Arab Spring was often cited as an illustration of the Internet’s liberating potential^3,4^ (but see ref. ^5^).

However, with the emergence of social media platforms (also known as ‘very large online platforms’ such as Facebook, Weibo and Telegram), expectations have shifted. Today, political hostility on social media ranks among the most pressing social and political challenges of the twenty-first century^6^. Most citizens and elites perceive online political discussions to be rife with smears, slurs and slander rather than constructive debate. Representative surveys consistently show that people find online political discussions to be much more aggressive, uncivil and hostile than face-to-face discussions^7^. Opinion leaders accuse social media platforms of eroding rather than upholding democratic discourse. As argued in a widely shared commentary, “platforms were almost perfectly designed to bring out our most moralistic and least reflective selves. The volume of outrage was shocking”^6^ (see also ref. ^8^). This quote encapsulates the view that social media platforms seem to be a key driver of rising hostility online. Inherent features of platform-based communication—such as fast-paced, written messages exchanged mostly between anonymous strangers—may inhibit empathetic concerns and breed hostility^9^. Ranking algorithms often reward controversial, moralistic messages, driving more attention to them^10,11^. In turn, the frequent exposure of social media users to divisive and opposing viewpoints can provoke hostile reactions, perpetuating a vicious cycle^12,13^. A strong version of this argument even holds that anyone who logs on to a social media platform becomes susceptible to hostility^14^.

Here we provide descriptive evidence on the nature of online political hostility by mapping its prevalence across diverse countries. We examine whether online hostility differs substantially and systematically between countries depending on characteristics known from previous research to elicit political polarization and instability. If so, online hostility may not solely be a product of the technological affordances of global social media platforms. Rather, social media platforms may also be intertwined with broader socio-economic and political tensions, produced outside the platforms.

Prior research has, in particular, highlighted how different forms of inequality are key sources of wider societal conflict^15,16^. Political and economic inequalities make the competition for upward mobility (or against downward mobility) more fierce at each step of the social ladder, generating substantial societal tension. For example, cross-national levels of inequality have been found to predict differences in motivations to acquire status via dominance—that is, through the use of fear and intimidation^17^. A rich literature in personality psychology has shown that people who are more motivated to acquire status are more aggressive in general^18,19^ and in political interactions^20–22^. Experimental studies have demonstrated that exposure to status threats activates aggression^23,24^. Prior studies have also found that more status-oriented individuals are more hostile in the online sphere: studies from the USA and Europe have shown that status-oriented individuals are more hostile in online political discussions^25^, share more hostile and false news^16,26^ and engage in more trolling^27^. Given that both offline and online hostility are grounded in similar dispositions, studies have also found remarkably high associations between individuals’ propensities to engage in hostile debates across very different topics^28^ and irrespective of whether these debates occur face-to-face or on social media platforms^7^. A recent study linking online behaviour to administrative records in Denmark even found that people with more hostile tweets have more criminal verdicts^29^. Social media can amplify the voices of a few highly motivated individuals. For example, a large proportion of political and medical misinformation in the USA originates from a handful of users^30–32^. Importantly, however, most prior studies on the interplay between offline circumstances and psychological motivations to engage in online hostility have focused on a select number of Western countries (in particular, the USA).

On the basis of this research, we expect systematic variation across countries in their levels of online political hostility. Status-seeking individuals may be more active or numerous in politically and economically unequal countries (that is, those that are less democratic and have greater income disparity), making all forms of interactions more hostile, including online discussions about societal matters. According to this account, psychological motivations for acquiring status would get activated or exacerbated to varying degrees by the levels of inequalities observed in one’s broader environment, thereby generating variable levels of hostile discussions about politics across countries.

**Fig. 1:**
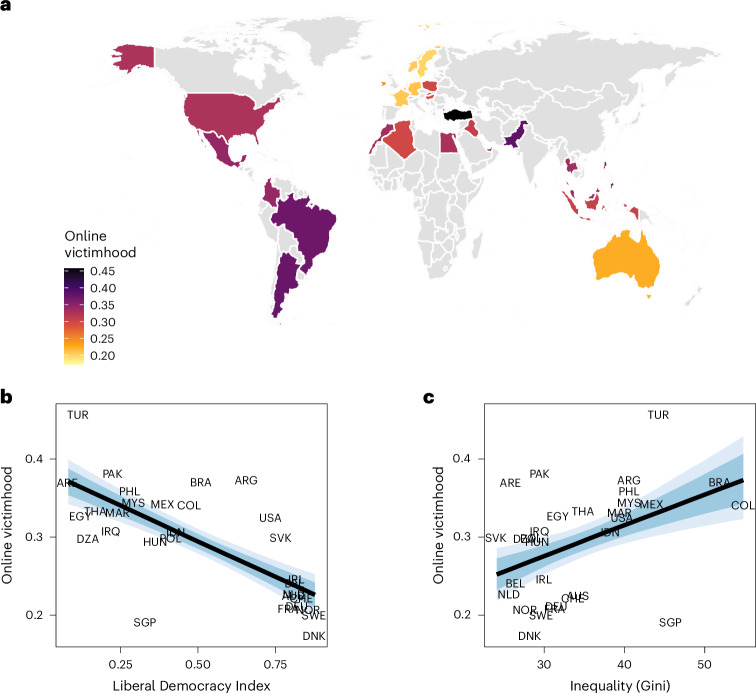
Online political hostility victimhood in the 30 countries surveyed. **a**–**c**, People living in countries with higher levels of liberal democracy and less economic inequality fall victim to less online political hostility on average. Panel **a** displays the country average level of online political victimhood and provides a geographical overview of the 30 countries in our study. Panels **b** and **c** display the association between victimhood and V-Dem’s Liberal Democracy Index and economic inequality (World Bank’s Gini estimates), respectively. The country labels are ISO three-letter codes and denote the country means as in **a**; see the details in Supplementary Information section E. The trend lines are estimated with a Bayesian multilevel regression with weakly informative priors. The blue shading denotes 67% and 89% CIs. *N* = 14,882.

To test descriptive implications of this account, we carried out a large international observational study of online political hostility. Remedying existing studies’ focus on Western countries, we surveyed 500 respondents resembling the national or online populations of 30 highly different countries from all inhabited continents on the globe (Fig. 1a). Countries were selected to maximize variation in the degree of democracy and economic equality within the set of nations with relatively large Internet penetration. This diverse sample allows us to compare the levels of online hostility across countries (democratic and less democratic, economically equal and unequal and so on). Our study also allows us to conduct a cross-cultural test of the prediction that striving for dominance at the individual level correlates with the political context. Importantly, our investigation does not allow us to provide direct causal evidence in favour of the theory. Rather, we build on the existing literature demonstrating this causal link and provide cross-cultural descriptive data consistent with it. In this effort, we heed calls by policymakers (for example, the United Nations^33^) and scientists^34^ to broaden the scope of scientific inquiries to the Global South.

In each country, respondents were surveyed about their experiences in online discussions about societal issues and their personalities (total *N* = 15,202; sample descriptive statistics are provided in Supplementary Information section A). Relying on these unique data, we first demonstrate that, as predicted, experiences of online political hostility vary systematically and strongly across countries according to their levels of political and economic inequality. Second, we demonstrate that this variation reflects a culturally ubiquitous role of status motivations in fuelling hostile online debates. Individuals with status-seeking motivations are more numerous in less democratic countries, and individuals with such motivations are consistently (that is, across democratic as well as less democratic countries) more likely to express their frustrations via hostility in both offline and online discussions.

These findings provide evidence that online political hostility cannot be understood in isolation from the larger socio-economic and political contexts of societies. In addition, our results suggest that social media may empower groups of people who are strongly dissatisfied with their standing in society.

## Cross-country differences in online political hostility

Which countries suffer more from online political hostility? A rich literature in the social sciences has documented how the grievances, resentments and frustrations caused by various forms of inequalities contribute to social conflict^15^. Here we zoom in on two forms: political inequalities measured by the level of democracy in a country and economic inequalities operationalized as the distribution of income.

It is a core mission of democratic regimes to reduce and institutionalize social conflict by equalizing access to political power. In a well-functioning democracy, the judiciary ensures that the rights and freedoms of citizens are respected, and elections allow all citizens to elect and be elected for leadership positions^35^. Democracies secure a peaceful transition of power^36^ and are much less likely to engage in armed conflicts. Democracies rarely wage war on other democracies and almost never succumb to civil war^37^.

It is also well established that economic inequality leads to social discontent and fosters political instability^15,38^. Recent psychological and epidemiological work shows that inequality has profound negative effects on people’s health and well-being^39^. One key mechanism that makes inequality a major source of societal conflict is that inequality fosters competition for status at every level of society: the larger the steps on the social ladder, the more fiercely people will fight to climb higher and avoid slipping lower^15,16,40^. In line with these mechanisms, research has found a clear association between status orientations and individual expressions of aggression as personal violence, political violence and online aggression^7,18,21^.

On this basis, we began our empirical investigation by examining the preregistered, descriptive predictions that countries with less (versus more) liberal democracy have higher levels of online political hostility (H1) and that countries with more (versus less) economic inequality have higher levels of online political hostility (H2). Finally, as a corollary of H2, we preregistered the prediction that countries with more (versus less) poverty have more online political hostility (H3). These three hypotheses are denoted H5–H7 in our preregistration. We changed only the order but not the content of our hypotheses; an overview is provided in Supplementary Information section B.

To test these descriptive hypotheses, we recruited samples of *N* = 500 resembling the national or online population in 30 countries via the YouGov survey agency (total *N* = 15,202). See Fig. 1a for an overview and the Methods for more details. To compare the observed levels of online political hostility between countries, we measured how often respondents encountered various forms of hostility—from ridicule to harassment—directed at people like them in the 30 days prior to the interview. This is a multi-item, self-reported measure of victimhood. While future work should explore the robustness of our findings to alternative measures, we believe this approach offers valid and reliable data today. First, it focuses on the main normative problem with hostility: the distress and offense it causes to those who are exposed to it. Second, to the best of our knowledge, this is an appropriate approach to make valid cross-country comparisons. We exploited well-honed techniques of survey research to ensure that our national samples represent the experiences of the citizens of the given societies, thus overcoming the problem of different platforms playing different roles and attracting different subpopulations in national online ecosystems. Also, relying on our respondents’ own minds to tally what is and is not hostile overcomes the problem that cross-cultural automated hostility detection is still in its infancy^41,42^. Third, our measure of online victimhood reflects the unique role that group-based experiences play online. Prior research has found that the bad reputation of online political discussions can be attributed not to negative personal experiences but to the elevated exposure to indirect hostility targeted at groups or individuals the person feels close to^7^. Finally, while self-reported measures pose the risk of bias from imprecise recollection and social desirability, we conducted a series of validation studies to quantify these biases in our measures (Methods and Supplementary Information section C). Consistent with our evidence of little bias, prior work has found that self-reported measures of hostility correlate highly with behavioural measures in experimental settings and on social media^7,43^. In Supplementary Information section G, we offer further support for our self-reported measures by reporting strong correlations with several existing country-level indicators of violence and hostility. All details and further elaboration on the measurements and their validity can be found in the Methods.

Figure 1 displays the levels of victimhood in online political discussions in the 30 countries surveyed, revealing considerable variation. People in most Western European countries experience political hostility less than once a month. For example, in Sweden the mean victimhood is 0.18 (89% credible interval (CI), (0.16, 0.20)), averaging across the five items, which is considerably lower than the average level in the 30 countries (global mean, 0.29; 89% CI, (0.27, 0.32)). Turkish respondents reported the highest average levels of online political victimhood with close to two to three incidents per month (mean, 0.46; 89% CI, (0.44, 0.48)).

Overall, we found support for hypotheses H1 and H2. First, more democratic countries (as measured by V-Dem’s Liberal Democracy Index^44^) have lower levels of online political hostility than less democratic countries, confirming H1. Countries that are one standard deviation more democratic experience five percentage points less online hostility (89% CI, (−0.07, −0.04)), which constitutes a remarkable 71% of a standard deviation in cross-country victimhood. We report simple rank-order correlations for all country-level variables in Supplementary Information section E. Second, supporting H2, people living in countries that are one standard deviation more economically unequal report falling victim to online political hostility three percentage points more often than people living in more egalitarian societies (89% CI, (0.01, 0.06)). Again, this difference is sizable, constituting 39% of a standard deviation in cross-country variance in victimhood.

We also found support for H3 as people living in countries that are one standard deviation higher in poverty experience three percentage points more online hostility (89% CI, (0.02, 0.05)). However, the association between poverty and political hostility disappears once we adjust our estimates for differences in democracy and inequality. By contrast, the estimates for democracy and economic inequality on hostility remain similar in size whether we adjust for the other two macro predictors or not. Supplementary Information section E reports the model details and bivariate correlations for the country-level variables. We map variation in online victimhood across social media platforms in Supplementary Information section F.

## Psychological roots of online hostility

Our prediction of a positive cross-sectional correlation between societal inequalities and online political hostility was grounded in psychological studies arguing that status motivations fuel hostility both offline and online. At the same time, it is possible that alternative psychological or sociological theories entail similar predictions. To further investigate the link between inequality and hostility, we shifted our focus from countries to individuals, providing a stronger foundation for the observed cross-country differences. If online hostility reflects (at least in part) the offline frustrations of status-motivated individuals, we would find that—across very different countries—people who are hostile online are also hostile offline, and that they have strong status motivations. While prior research has found evidence for both of these associations in the USA and Denmark^7^, here we asked whether these associations generalize across the world.

**Fig. 2:**
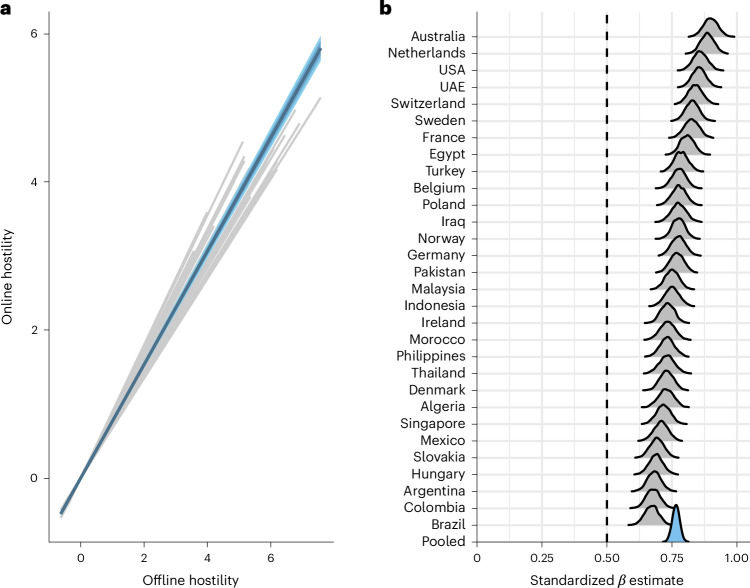
People who are hostile in political discussions online are also hostile offline. **a**, Predicted level of online political hostility as a function of reported offline political hostility. The blue line denotes the pooled estimate. The blue ribbons display 67% and 89% CIs. The grey lines indicate country-level estimates. The figure displays predicted hostility for male respondents below the country-sample median age and without higher education. **b**, Posterior distributions for the standardized *β* estimates for the entire dataset (blue) and each of the 30 countries (grey). Dashed vertical line denotes the pre-registered threshold for a strong relationship. *N* = 13,628. Details are provided in Supplementary Information section E.

Specifically, we preregistered the descriptive hypothesis that people who report more (versus less) offline hostility also report more online hostility (H4). We relied on behaviourally validated measures from prior research^7,43^, and, to make our prediction more ambitious, we predicted the relationship to be strong, defined as a standardized regression coefficient (*β*) larger than 0.5. In support of H4, Fig. 2 demonstrates that those who are hostile on the Internet are also hostile in face-to-face conversations (standardized *β* = 0.77; 89% CI, (0.74, 0.79)). The associations are above our preregistered threshold in every country surveyed.

Prior research suggests that status-driven motivations play a key role in fuelling online hostility, particularly through individual differences in status-driven risk-taking^7,16,45^. However, this research has been conducted exclusively in a few Western, democratic countries. Here we asked whether this association generalizes globally, providing a plausible psychological foundation for the strong association between societal inequalities and online hostility. To this end, we predicted that people higher (versus lower) in status-driven risk-taking are also more hostile online (H5). We expected to find at least a medium-size difference, defined as a standardized *β* coefficient larger than 0.1.

We also predicted that people who feel more (versus less) meaningless are more hostile online, and we expected to find a positive interaction between status drive and meaninglessness. We found no evidence for these two predictions, and for the sake of brevity we relegate these results to Supplementary Information section H.

We sought to benchmark the roles of status-driven risk-taking and meaninglessness to a popular psychological predictor, affective polarization. To our surprise, but consistent with a recent critique of the polarization literature^46^, we found no evidence for a relationship between polarization and political hostility either; see Supplementary Information section I for the details.

**Fig. 3:**
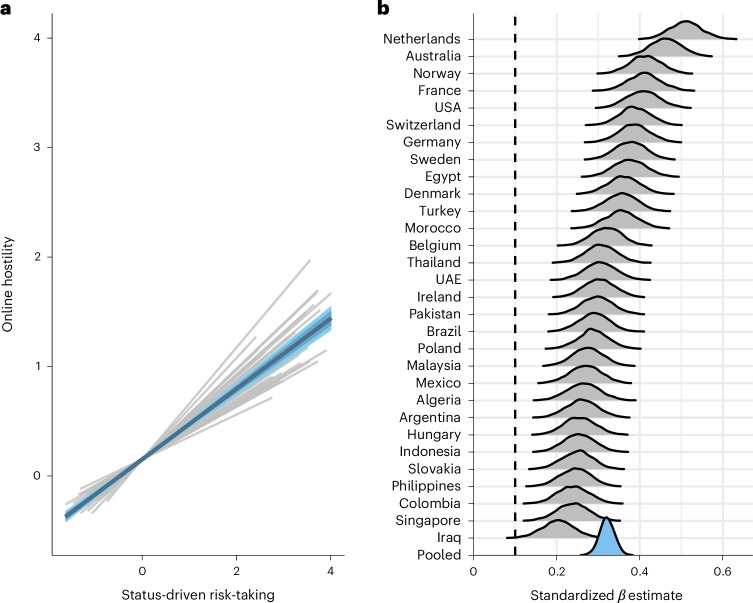
Status-driven risk-taking is a robust predictor of online political hostility in every country. **a**, Predicted level of online political hostility as a function of status-driven risk-taking. The blue line denotes the pooled estimate. The blue ribbons display 67% and 89% CIs. The grey lines indicate country-level estimates. **b**, Posterior distributions for the standardized *β* estimates for the entire dataset (blue) and each of the 30 countries (grey). Dashed vertical line denotes the pre-registered threshold for a medium-size relationship. *N* = 14,513. Details are provided in Supplementary Information section E.

Figure 3 shows that—supporting H5—higher status-driven risk-taking is strongly related to online political hostility (standardized *β* = 0.32; 89% CI, (0.29, 0.35)). Again, although there is some cross-country variation in the relationship between status-driven risk-taking and online hostility, the posterior distributions are all above the threshold of 0.1.

Moving past our preregistered analyses, we next ran exploratory models in an attempt to link our macro- and micro-level findings. Specifically, we explored whether status motivations are linked to the higher online hostility observed in more unequal and less democratic countries. One possibility is that economic and political inequalities amplify the association between status-seeking and online hostility such that this association is stronger in more unequal and less democratic countries. Another possibility is that there are more status-driven people in more unequal and less democratic countries; that is, political and economic inequalities encourage more people to adopt aggressive status-seeking strategies^17^. This is consistent with the notion that status-seeking is a stable trait, since stability refers to rank-order persistence rather than fixed mean levels^47^.

First, we tested whether there is a cross-level interaction between status drive and the level of democracy or the level of economic inequality. Do people living in less democratic and economically unequal countries fall victim to more hostility because status-driven people in their countries are more hostile than status-driven people living in (more) democratic and equal countries? In Supplementary Information section J, we demonstrate that our data do not support this possibility. Instead, there is some evidence of the reverse: individuals with high status-driven risk-taking are more hostile if they live in a more democratic or, to a lesser extent, in a less equal country. We estimated the hostility gap between two groups—one standard deviation apart in status drive—as a function of the level of democracy. Status-driven people are more hostile in all countries, but the gap is substantially larger in the most democratic than in the least democratic country (*β* = 0.14; 89% CI, (0.07, 0.20)). For the same comparison in the least and most equal countries, the estimate is negative but highly uncertain (*β* = −0.10; 89% CI, (−0.21, 0.00)).

We found partial empirical support for the second possibility that there are more status seekers in more unequal countries. Specifically, we found that people in less democratic countries on average score higher on status-driven risk-taking than people in democratic countries (*β* = −0.05; 89% CI, (−0.07, −0.03)). However, for poverty (*β* = 0.03; 89% CI, (0.01, 0.05)) and inequality (*β* = 0.01; 89% CI, (−0.01, 0.03)), the relationship is less robust to the simultaneous inclusion of all three variables (Supplementary Information section J).

**Fig. 4:**
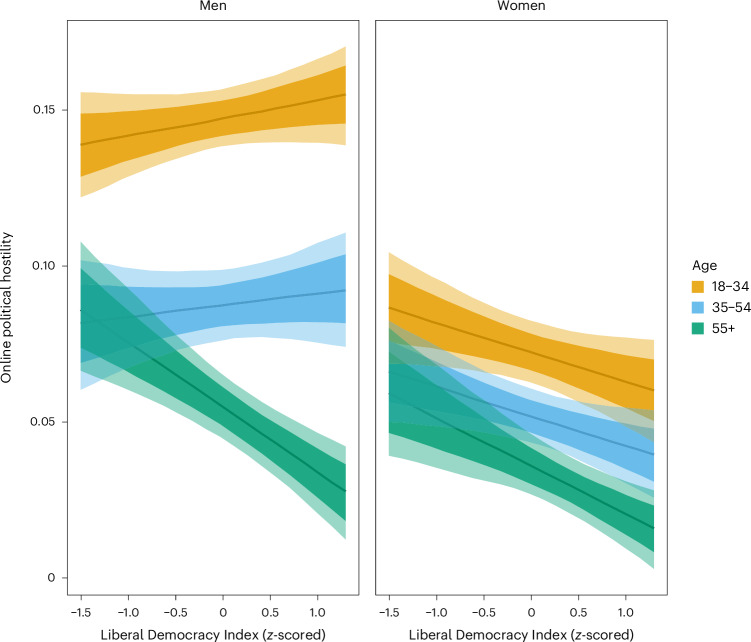
Young and middle-aged men are equally hostile in democratic and non-democratic countries. All other demographic groups report less hostility in more democratic countries. The figure displays the predicted level of online political hostility on the raw scale (from 0 to 1 and not *z*-scored as in previous plots) as a function of the Liberal Democracy Index (*z*-scored) across genders and different age categories. The ribbons denote 67% and 89% CIs. *N* = 14,642. Details are provided in Supplementary Information section K.

Exploring the cross-country variability in the hostility of demographic groups adds further insight to these results. Specifically, we were interested in the hostility of the most status-driven and aggressive demographic group—namely, young men—across different societies^18^. Our results, displayed in Fig. 4, reveal most importantly that young men are consistently the most hostile group in online political discussions (Supplementary Table K.1). This implies that countries with younger populations are expected to face more hostility on average. We demonstrate that this may contribute to the higher average hostility observed in less democratic countries in our sample by comparing each country’s predicted hostility under its observed age distribution to predictions under a common reference age distribution pooled across countries (Supplementary Information section K). To illustrate, we compare Sweden to the United Arab Emirates (UAE), the most and least democratic countries in our sample. Young adults (aged 18–34) constitute 28% of the population in Sweden but 50% in the UAE, the least democratic. The difference in age composition alone is predicted to reduce average hostility in Sweden by 0.36 standard deviations (89% CI, (−0.47, −0.25)), while increasing it in the UAE by 0.59 standard deviations (89% CI, (0.37, 0.77)), scaling to country-level variance in self-reported hostility.

We also found tentative evidence for an interaction between demography and democracy: most demographic groups in society are less hostile if they live in more democratic societies. However, the two most hostile demographic groups—namely, young (18–34) and middle-aged (35–54) men—are consistently hostile across different levels of democracy (the details are provided in Supplementary Information section K).

These findings suggest that the higher levels of online hostility in more unequal and less democratic countries may be related to the fact that there are more highly status-driven individuals in less democratic countries. Furthermore, the grievances experienced under less democratic regimes may fuel hostility in sociodemographic groups that are not habitually engaged in aggressive political tactics—that is, older and female respondents. In democracies and economically equal countries, in contrast, online hostility may be driven more by the usual suspects of aggression: young male status seekers^48,49^.

## Discussion

In this study, we found that more unequal and less democratic countries suffer more from online hostility than more equal and democratic countries, and that the same status-driven people are hostile in face-to-face and in online discussions. Exploratory analyses further revealed that status-driven individuals are more prevalent in less democratic countries. At the same time, the difference in hostility levels between those with high and low status-driven motivations is more pronounced in democratic countries.

Our findings must be interpreted in light of several limitations. First, we focused on making valid cross-country comparisons and hence opted for the well-honed tools of survey research. Although extensive tests support the validity and reliability of our self-reported measures, behavioural replications are an important next step, and, with recent advances in machine learning, cross-country comparisons of behavioural hostility may soon become feasible^42^.

Second, although motivated by theoretical accounts of the drivers of hostility, this study remains descriptive and does not establish causal relationships or mechanisms. Descriptive research is increasingly recognized as a crucial step in developing valid theories and improving the generalizability of existing causal theories, especially in the dynamic domain of digital media research^50^. That being said, what causes the observed differences in hostility across countries and individuals remains an open question. Our own theorizing favours a mechanism wherein offline differences in personality and socio-economic environment shape online behaviour. But our observational data cannot rule out or quantify the opposite mechanism. It remains conceivable that online hostility also shapes the offline world, influencing respondents’ (self-reported) personality and their broader social and political environments. Some of our correlations may be inflated by individuals who are radicalized online and subsequently turn hostile offline and report elevated status-driven motivations. It is plausible that hostile online political discussions undermine social forces pushing for more democracy and equality. Future research should examine longitudinal data on local inequalities or external shocks to individual status motivations to quantify their causal impact on online hostility.

While we are not able to establish the direction of causality on the basis of our observational data, exploratory analyses show that public perceptions of social media reflect both possibilities (Supplementary Information section L). Across countries, we found that people see social media as generating societal turmoil and enabling government oppression, but also as providing a means of liberation from government censorship (see also refs. ^51,52^). Agreement with the turmoil perspective is more pronounced in democratic countries, whereas the liberation narrative tends to resonate more in less democratic settings (see Supplementary Information section L for a longer reflection on these results; see also refs. ^53,54^ for similar observations). Paradoxically, this suggests that in countries with lower levels of online hostility, social media is more often seen as a source of turmoil, whereas in countries with higher online hostility, social media is perceived as a source of liberation. Importantly, these patterns indicate that public perceptions themselves mirror the unresolved causal tension: whether social media is primarily shaped by offline inequalities and political conditions, or whether it actively reshapes them. Rather than resolving this ambiguity, the public appears to intuitively acknowledge both directions of influence, reinforcing the view of social media as a complex force in society.

The nuanced views of the public, as well as differences in perceptions across countries, potentially indicate that a key consequence of large online platforms is that they increase what has been labelled ‘connectivity’^7^—that is, the multiplication of connections between individuals coupled with the increased speed at which information can reach people (see also ref. ^55^). As with other mass communication technologies, increased connectivity ensures faster social coordination within subgroups and at a larger scale, which can be used both to liberate and to oppress people^52^. However, the constant emergence of new societal issues, fuelled by online activists, is likely to maintain a sense of turmoil and instability^56^. In this regard, it is relevant to note that we found that the most hostile individuals are, paradoxically, located in the most equal countries. In an environment with high connectivity, the behaviour of this small core of disenfranchised activists can reach millions in a matter of hours^30^, facilitating a sense of turmoil, especially as the frustrations of these activists may have gone unnoticed prior to the advent of social media.

These complexities notwithstanding, the present findings suggest that efforts to reduce hostility online must consider the broader socio-economic and political contexts. While platform regulations may help address immediate harms, the results here point to the deeper societal tensions that are connected to online hostility. Addressing these conditions may therefore be essential to disrupting a mutually reinforcing cycle between societal discontent and digital aggression.

More broadly, our findings highlight the importance for social media researchers to move beyond the Western world to understand the role of social media in our lives (see also ref. ^57^). First, online political hostility is a larger problem in the less democratic and more economically unequal countries such as those in the Global South. In particular, scholars concerned with political hostility in the USA should note that, in our sample of 30 countries, the USA ranks near the middle in terms of reported victimhood. Granted, hostility in the USA is higher than in other liberal democracies, but it is average for a country with its level of economic inequality. If the macro correlations generalize outside our sample, it means that the many countries with less democratic or less economically equal societies face more significant problems.

Finally, as noted, people in less democratic or more unequal countries are also more likely to see online platforms as a source of liberation. An exclusive focus on the detrimental effects of social media may overlook the diversity of global experiences. In a world where democracies are again on the decline globally^58^, it is important that researchers and policymakers invest resources in understanding how to harness the enhanced connectivity offered by online platforms such that these hopes of liberation give rise to more democratization.

## Methods

### Preregistration and ethics

The materials, hypotheses and analyses were preregistered at https://osf.io/hfa2k/ on 7 April 2023. We report hypotheses, predictions, outcome variables and analyses as preregistered, except if otherwise indicated.

All participants (including those participating in the follow-up studies) provided informed consent and were reimbursed according to their standing agreements with YouGov. The study design was approved by Aarhus University’s Research Ethics Committee (BSS-2022-113). All materials, data and code have been deposited on the Open Science Framework at https://osf.io/9r4vs/.

### Data and generalizability

We collected online survey data (total *N* = 15,202) between 14 April and 7 July 2023 from 30 countries: Algeria, Argentina, Australia, Belgium, Brazil, Colombia, Denmark, Egypt, France, Germany, Hungary, Indonesia, Iraq, Ireland, Malaysia, Mexico, Morocco, the Netherlands, Norway, Pakistan, Philippines, Poland, Singapore, Slovakia, Sweden, Switzerland, Thailand, Turkey, the UAE and the USA. Our key consideration in selecting the countries was to balance data quality with diversity. We employed the leading polling firm, YouGov, and sampled only countries where YouGov maintains and controls the quality of their own panels (as opposed to relying on subcontractors). From the available YouGov panels, we chose those that maximized the variance on two macro predictors: liberal democracy and inequality. Our final sample covers diverse cultures from all six inhabited continents. Admittedly, our observational data only capture a snapshot of a specific period in a given country, and our estimates of hostility levels may be influenced by sociopolitical events that took place close to our data collection^56^. We reviewed notable sociopolitical events around our data collection in each country and concluded that these events were unlikely to undermine the validity of our estimates (Supplementary Information section D).

Our decision to collect observational data through online surveys for this cross-cultural project is based on several key considerations. First, while real-life social media data (shares, comments and so on) offer the advantage of capturing true, spontaneous online behaviour, data from the most popular social media platforms today are extremely difficult—if not impossible—to access. As a further complication, social media platforms delete some of the most hostile messages, making it impossible to study them ex post. Finally, even if behavioural data from multiple countries were accessible, we would still face the problem that there are no cross-culturally validated classifiers of online hostility. Machine learning algorithms developed to detect hostility at scale remain coarse, making exposure difficult to demonstrate^41^. They are also limited to the corpus of social media posts in a single language, most often English (although see ref. ^43^). To further assess the feasibility of behavioural data for cross-national comparisons, we analysed sentiment from a large-scale Twitter dataset^59^. Our findings reinforce the dataset authors’ caution that while such data may be useful for tracking temporal changes within countries, they are not well suited for robust cross-country comparisons (Supplementary Information section T).

Social media platforms, moreover, are distinct economic actors competing for users’ attention on the market of information. They offer unique features and user experiences that make it difficult to compare behaviours and perceptions across several platforms. For instance, X (formerly Twitter) is optimized for dynamic public conversations consisting of brief, text-based posts, whereas Instagram is designed for photo sharing, and its commenting features make it almost impossible to engage in a conversation with multiple people. Users also vary from platform to platform, if only because of the fragmentation of the Internet into networks of users with distinct hobbies, socio-economic characteristics and political inclinations. Given the diversity of online platforms and communities, we judged that relying on a standardized, platform-neutral survey would be the most appropriate way to probe respondents’ experiences with online hostility in general, whether it happens on 4Chan or Reddit, or among Argentinian or Turkish users. To design the survey, we drew on a rich literature of research on cross-cultural differences in, for example, antisocial punishment^60^, looseness and tightness^61^, honesty and rule violations^62^, parochialism^63^ or prejudice during the COVID-19 pandemic^64^.

We surveyed around 500 respondents in each country using YouGov’s native platform. Our preregistered design calculations indicated that this sample size would yield more than 80% likelihood of detecting the hypothesized individual-level associations (H4 and H5) in each country, assuming realistic effect sizes and using 89% CIs. The number of countries was chosen on the basis of financial constraints.

Questionnaires were translated into the dominant local language(s) in each country by professional translators. Each translation was proofread by two independent native speakers of the target language. YouGov quota-sampled respondents with the aim of approaching representativeness of each national online population. Quotas were defined using available census statistics in each country on age and gender as well as—where feasible—region (in all countries, except Algeria, Iraq and Singapore) and education (in Australia, Brazil, Denmark, France, Germany, Mexico, Sweden and the USA). Participants were screened out if they failed either of two attention checks positioned at the beginning of the survey or if they reported having no social media accounts. We report platform usage by country in Supplementary Information section A.

### Measures

#### Victims and perpetrators of online political hostility

We relied on self-reported measures of hostility, which have been shown to be a valid measure as they correlate highly with hostile behaviour on social media^43^. Our primary objective was to measure three aspects of political hostility: online victimhood, online hostility and offline hostility. Following best practices^65^, we defined, at the beginning of our survey, what we mean by political discussions to focus respondents’ attention on a shared range of experiences: “The following questions concern your experiences with discussions about societal issues. By societal issues, we mean any issue related to politics, social problems or disagreements between groups in society. These could be related to either local (for example crime, housing), national (for example economy, inequality), or international issues (for example war, pandemic).”

Next, we measured online political victimhood: “Please think about the past 30 days, specifically. How often did the following happen to you in discussions on societal issues that occurred on the Internet, such as on social media or in comments sections?” We built on prior research^7^ to develop a five-item battery assessing various forms of hostile behaviours ranging from relatively mild (“I saw content that ridiculed people like me”) to serious offenses (“I saw content that threatened or harassed someone like me”). Throughout the survey, we randomized the order of items in all multi-item batteries. To avoid potential bias, we offered a “prefer not to answer” option on all questions^66^.

A large benefit of building our macro comparisons on our self-reported measures is that it fully reflects the subjective nature of victimhood. The offending character of a remark, after all, is in large part defined by the negative emotions it elicits in the victim. Certain words can be experienced as hurtful even when the sender had no intention to hurt, or if most observers would not find them harmful. Words can offend thousands if they go viral, even if the sender intended it only for a single person. Conversely, even horrendous slurs that would offend most people could go virtually unnoticed—for example, if a discussion is abandoned or if the slur is removed promptly by a moderator. Unlike approaches relying on behavioural examples of hostility, our victim-focused self-reported measure avoids both of these errors.

Note that we used the phrase ‘[people] like me’ to specify the target of hostility. Most offensive content people encounter online does not target them personally^7^. A viable measure of victimhood must therefore take into account indirect hostility—that is, hostility directed at others the respondent feels connected to, such as those sharing their gender, ethnicity or political affiliation. As a validation of our approach, Supplementary Information section G demonstrates that our victimhood measure consistently correlates more with other forms of hostility and violence at the country level than self-reported hostility.

To measure who is hostile, we reused the same five hostility items, switching the perspective from victim to perpetrator (for example, “I ridiculed someone for their views or actions”). Specifically, we used these batteries in the first half of the survey and at the very end—that is, twice. We randomly varied whether the first or the second battery asked about perpetration of online or offline hostility. We defined the offline context as “discussions on societal issues that occurred in person, such as at meetings or public or private events” and the online context as discussions that “occurred on the Internet, such as on social media or in comments sections”.

Before our questions about hostility, we asked participants how often they participated in discussions about social issues. Respondents who never participated were not shown the perpetrator questions and were assumed never to be hostile (but could still be passive victims). We demonstrate in Supplementary Information section M that our conclusions do not change if we simply remove these participants from the analyses.

We report measurement invariance analyses for all of our multi-item measures in Supplementary Information section N. The results support scalar invariance for online and offline hostility, and partial scalar invariance for victimhood, which warrants caution in interpreting mean comparisons for the latter.

#### Psychological predictors of hostility

We now turn to our measurement of psychological predictors of hostility. To test our hypothesis that status-driven risk-takers are more likely to be hostile in online political discussions, we used a shortened, four-item version of the Status-Driven Risk Taking questionnaire. This measure is designed to identify people for whom risk-taking is “being undertaken solely as a means to an end, being motivated entirely by the prospect of gains in money, power, or prestige”^45^. We identified the four best items of the original 14-item measure using factor analysis with data from 19,000 observations. Sample items include “If I could become rich and famous by winning a major competition, I would put my life on the line to win it” and “I would enjoy being a famous and powerful person, even if it meant a high risk of assassination”. Note that YouGov, our survey provider, deemed the latter item too sensitive to be used in Egypt, Iraq and the UAE, and we employed a slightly modified version in these countries. Supplementary Information section O demonstrates the robustness of our analysis to excluding these countries or this item from our analyses. We measured the level of agreement with these items using a seven-point Likert scale ranging from “strongly disagree” to “strongly agree”.

We measured anomie or meaninglessness using an item from the World Values Survey, which asks respondents to indicate “how much freedom of choice and control [they] feel [they] have over the way [their] life turns out” on a ten-point scale from “no choice at all” to “a great deal of choice”.

Finally, we adapted the standard affective polarization measure from the American National Election Study to apply across countries^67^. We asked respondents about their feelings towards “political groups that *support / are critical of* the current government” on a seven-point Likert scale ranging from “strongly dislike” to “strongly like” and took the absolute difference between the two items as our measure of affective polarization.

#### Follow-up studies to ensure the robustness of our hostility measures

We conducted a series of follow-up studies on nationally representative US samples (total *N* = 4,294) to test the validity and reliability of our key measures of victimhood and hostility. We offer a brief summary of these studies here and report detailed descriptions of the designs and findings in Supplementary Information section C.

Follow-up Study 1 (*N* = 1,296) investigated whether our measure of victimhood varies depending on the target of hostility—whether it is directed at the respondent personally, their ingroup or anyone online. Our findings indicate that the measure specifically captures group-based victimhood, distinguishing it from both general observations of hostility and direct personal victimhood online.

Follow-up Study 2 (*N* = 849) examined whether respondents might under-report hostile behaviours due to social desirability concerns. To assess this potential bias, we implemented a virtual dice-rolling game—a method specifically designed to reduce social desirability in self-reports^68^. Our results revealed no evidence of social desirability bias in measures of victimhood or hostility.

Follow-up Study 3 (*N* = 864) explored whether repeated exposure to online hostility desensitizes respondents, potentially biasing victimhood estimates. Following an established procedure to gauge the effects of desensitization to hostility^69^, participants were exposed to a series of hostile messages, after which we assessed their perceived victimhood. We found no evidence that repeated exposure diminished perceptions of victimhood, nor did it significantly influence individuals’ likelihood of reporting that they engage in hostile behaviour themselves.

Follow-up Study 4 (*N* = 1,288) tested whether national narratives on online hostility shape individuals’ experiences of victimhood. We found that low-prevalence narratives had no effect, while high-prevalence narratives increased self-reported victimhood. This suggests that individuals in the USA generally assume lower levels of online hostility, adjusting their perceptions only when confronted with widespread hostility narratives. It also indicates that systematic differences in national discourse around the prevalence of online hostility may influence our victimhood estimates. However, if anything, such narratives probably lead to an underestimation of victimhood. As demonstrated in Supplementary Information section L, individuals in more democratic countries, where self-reported victimhood is lower, tend to portray social media more as a source of turmoil, while individuals in less democratic countries, despite reporting higher victimhood, express less concern about turmoil. This suggests that cross-country differences in victimhood may be even greater than our estimates indicate.

#### Country-level predictors of political hostility

As our measures of country-level characteristics, we relied on the V-Dem Institute’s Liberal Democracy Index^44^ and the World Bank’s estimates of income inequality (Gini coefficient) and poverty (defined as the share of the population who lived on an income below US$5.50 a day in 2011 international (purchasing power parity) prices). The World Bank does not publish inequality or poverty estimates for Singapore, so we relied on the CIA World Factbook’s Gini estimate.

#### Respondents’ demographics

Three demographic covariates—age, gender and education—were obtained from YouGov. We dichotomized each to distinguish between respondents older or younger (that is, not older) than the country sample median, respondents identifying as male versus female and respondents who completed higher education versus those who did not.

### Modelling

All regression estimates are from Bayesian multilevel models using a Gaussian likelihood function. All our models assume weakly informative priors, which do not meaningfully influence parameter estimates (compared with flat priors) but help models converge (the details are provided in Supplementary Information section P). All our models included survey weights provided by YouGov and were implemented in R (v.4.2.1) using Stan (v.2.18) and the brms (v.2.18.0) package^70,71^. Following McElreath^72^, we quantified uncertainty in our estimates by reporting both 67% and 89% CIs (as preregistered).

For models testing country-level predictions, we regressed political victimhood on one of our three main predictors and a varying intercept for countries. For models testing psychological predictors of hostile behaviour, we regressed our self-reported measure of political hostility on the focal psychological predictor (offline hostility, status-driven risk-taking or anomie) as well as three demographic covariates: age, gender and education. We transformed all continuous variables into *z*-scores (using country means and standard deviations); hence, our main regression estimates can be interpreted as standardized *β* coefficients.

### Robustness tests

We offer a number of robustness tests for our main analyses. First, in Supplementary Information section G, we demonstrate that our country-level conclusions do not hinge on the specific indicators of democracy and economic inequality employed. Second, as an additional test of H1, we exploited the study design’s randomization of online and offline hostility batteries and conducted both within- and between-participants equivalence tests on the average levels of self-reported hostility across discussion environments. The results reported in Supplementary Information section Q demonstrate that levels of hostility are virtually identical, even when relying on only the first battery in the questionnaire (thereby guarding against common source bias). Third, we addressed concerns about presumed differences in normatively permissible behaviour online versus offline by estimating the correlation of hostile behaviour across discussion environments for each of the five items in our index. Our logic is that even if such norm asymmetries existed (although ref. ^7^ found no evidence), they would probably concern milder forms of hostility. People may disagree about where the line is when it comes to ridicule offline versus online, but it is unlikely that the same ambiguity exists about harassment, for example. The results in Supplementary Information section R demonstrate that the correlation between offline and online hostility is equally strong across all forms of hostility. Fourth, we further examined the robustness of our findings by conducting a within-country test of the inequality–hostility relationship using multilevel regression and poststratification in the USA. This analysis demonstrates that income inequality predicts higher levels of online hostility even when national-level institutional and cultural factors are held constant. The full methodological details and results are reported in Supplementary Information section S.

### Reporting summary

Further information on research design is available in the Nature Portfolio Reporting Summary linked to this article.

## Supplementary information

Supplementary Information Supplementary Information sections A–T.

Reporting Summary

Peer Review File

## Data Availability

All materials and data are available via the Open Science Framework at https://osf.io/9r4vs/.

## Code availability

All code is available via the Open Science Framework at https://osf.io/9r4vs/.
