# Supplementary material for: Social media users experience more political hostility in less economically equal and less democratic societies
Source: Nat Hum Behav. 2026 Apr 3;10(6):1083–93. doi: 10.1038/s41562-026-02432-5 (PMC13290475; doi:10.1038/s41562-026-02432-5)
Supplement: Supplementary file 1 — Supplementary Information sections A–T. [file 41562_2026_2432_MOESM1_ESM.pdf]

nature human behaviour

Supplementary information

https://doi.org/10.1038/s41562-026-02432-5

Social media users experience more political hostility in less economically equal and less democratic societies

In the format provided by the authors and unedited

Contents

A Descriptive Statistics, Sample Demographics, Scale Reliability **2**

B Overview of Hypotheses **9**

C Follow-up studies assessing the robustness of our hostility measures **10**

D Notable socio-political events during data collection **19**

E Supplementary results about the macro-level estimates and correlations of online hostility **21**

F Platform differences in online political victimhood across countries **27**

G Exploratory analyses of country-level correlates of online political hostility **29**

H Feelings of meaninglessness do not predict online political hostility **36**

I Affective polarization does not predict online political hostility **38**

J The role of status-driven risk taking across different countries **40**

K Young male syndrome in equal and unequal societies **42**

L How do people across the world perceive the impact of social media on politics? **47**

M Excluding respondents who never participate in political discussions **54**

N Assessing measurement invariance **56**

O Diverging measurement of status-driven risk taking and the impact of social media on politics **58**

P Weakly informative priors in our models **61**

Q Equivalence of political hostility online and offline **62**

R Online versus offline hostility by items **66**

S Hostility and inequality across US States **67**

T Analyzing Negativity on Twitter across Countries **71**

A Descriptive Statistics, Sample Demographics, Scale Reliability

Table 1 displays the main demographics of our sample, using survey weights. Figure 1 displays the distribution of talking about social issues in each country. Figure 2 quantifies platform usage across countries. Figure 3 displays the alpha reliability of all our additive indices. Figure 4 displays the correlation matrix of individual level variables. Finally, Figure 5 benchmarks our data using our estimated popularity of Facebook against external data from the Meta Ad Manage, accessed via https://datareportal.com. Note that calculating the number of Facebook users per country is not an easy task, as discussed in detailed on DataReportal's Methods page, https://datareportal.com/notes-on-data. Hence these numbers should be taken with a grain of salt. Furthermore, even apart from the noisiness of the FB benchmark, the absolute levels between the benchmark and our data are not comparable, because national FB user numbers are calculated as a proportion of eligible population (above the age of 14). Meanwhile, our samples include only adults (18+) and only people who use at least one social media platform. Nonetheless, apart from a few countries (e.g. Pakistan), the countries where Meta reports Facebook to be more popular also include more Facebook users in our sample.

Supplementary Table 1: Sample demographics by country

Country	N	Med Age	Women	Highr Ed.	Income-Lwr	–Mid	–Hgh	–NA
Algeria	502	33-37	0.58	0.77	0.7	0.1	0.08	0.13
Argentina	509	43-47	0.52	0.26	0.16	0.23	0.27	0.34
Australia	504	43-47	0.56	0.32	0.3	0.26	0.28	0.16
Belgium	507	48-52	0.53	0.32	0.55	0.14	0.01	0.3
Brazil	508	38-42	0.53	0.18	0.75	0.06	0.05	0.15
Colombia	505	38-42	0.53	0.47	0.5	0.33	0.05	0.11
Denmark	507	48-52	0.51	0.33	0.24	0.34	0.21	0.21
Egypt	507	33-37	0.5	0.85	0.21	0.44	0.2	0.14
France	505	48-52	0.54	0.18	0.4	0.43	0.04	0.13
Germany	515	48-52	0.53	0.21	0.38	0.41	0.02	0.19
Hungary	507	48-52	0.55	0.23	0.59	0.22	0.03	0.17
Indonesia	507	28-32	0.48	0.41	0.38	0.43	0.13	0.06
Iraq	504	28-32	0.56	0.86	0.27	0.04	0.04	0.64
Ireland	505	43-47	0.5	0.53	0.26	0.38	0.23	0.12
Malaysia	516	28-32	0.59	0.54	0.4	0.32	0.13	0.16
Mexico	508	38-42	0.54	0.22	0.65	0.11	0.05	0.19
Morocco	503	33-37	0.58	0.5	0.7	0.07	0.02	0.21
Netherlands	507	48-52	0.53	0.34	0.41	0.27	0.09	0.23
Norway	505	48-52	0.51	0.3	0.15	0.45	0.2	0.2
Pakistan	502	33-37	0.57	0.77	0.21	0.32	0.41	0.06
Philippines	507	33-37	0.53	0.64	0.41	0.35	0.14	0.1
Poland	507	43-47	0.53	0.44	0.28	0.45	0.16	0.11
Singapore	510	43-47	0.53	0.58	0.3	0.38	0.21	0.11
Slovakia	506	43-47	0.53	0.37	0.61	0.17	0.02	0.2
Sweden	509	48-52	0.52	0.43	0.14	0.37	0.35	0.14
Switzerland	505	48-52	0.52	0.27	0.58	0.2	0.02	0.19
Thailand	504	43-47	0.52	0.57	0.33	0.38	0.3	0
Turkey	506	38-42	0.54	0.52	0.38	0.29	0.21	0.12
UAE	507	33-37	0.33	0.76	0.59	0.2	0.11	0.11
USA	508	43-47	0.53	0.57	0.4	0.39	0.11	0.1

Supplementary Fig. 1: Distribution of talking about social issues in each country. Respondents who never talk about social issues are assumed to be never hostile themselves – but they can still be victims of online political hostility.

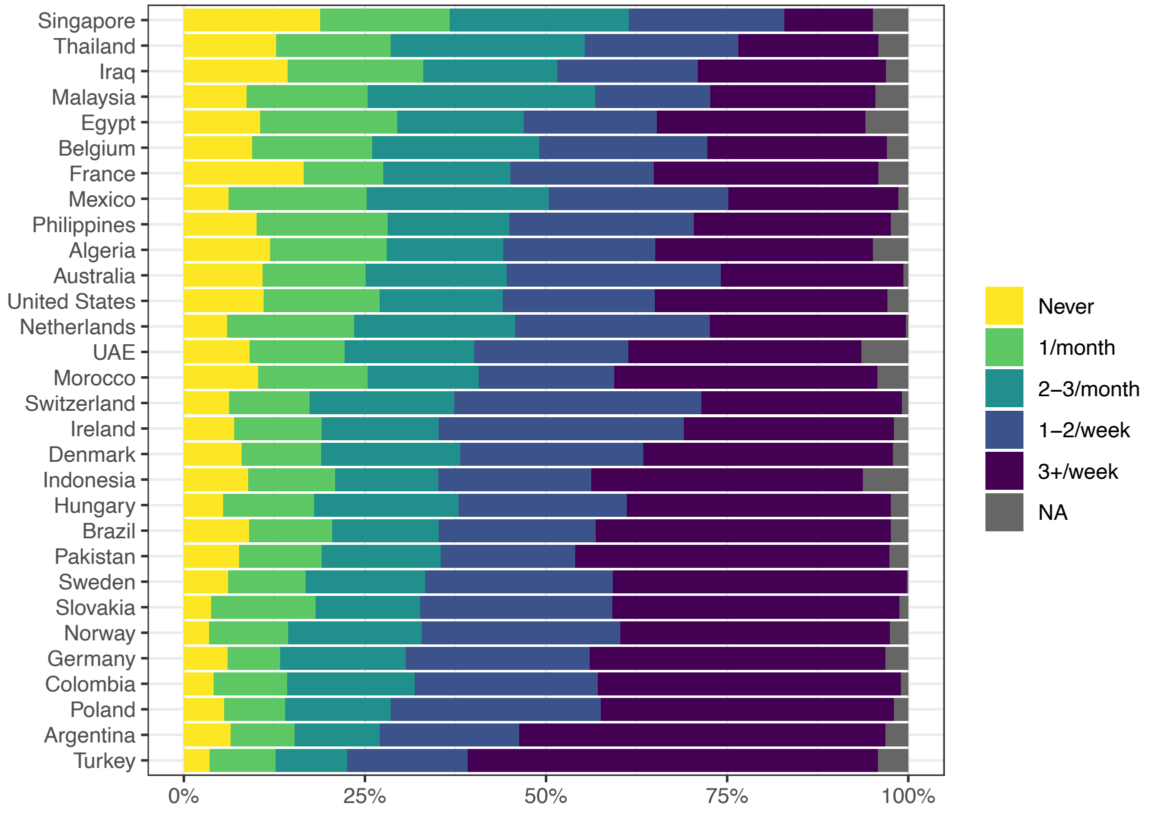

Note: We used survey weights to calculate the distribution of responses.

Supplementary Fig. 2: Distribution of social media platform use across countries. Lighter colors denote a higher share of society using a given platform.

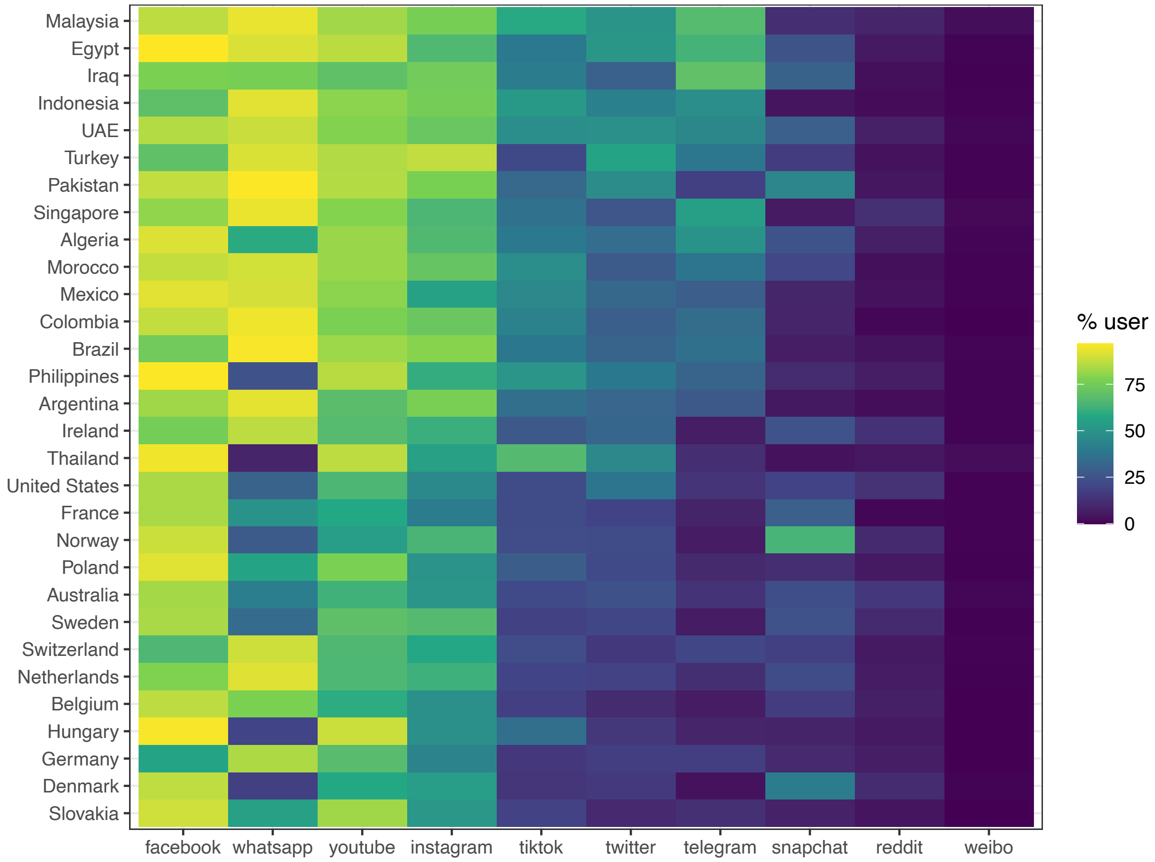

Note: Our questionnaire asked respondents "What type of social media accounts do you use?" Respondents could choose as many platforms as they wished. We employed survey weights in calculating the proportions in the figures.

Supplementary Fig. 3: Alpha reliability estimates for each of our seven individual level variables in each country. Our indices of hostility (online and offline as perpetrator, and as online victim) and status-driven risk taking (SDRT) show very high reliability. The three variables on the perceived impact of social media on politics (Liberation, Oppression, and Turmoil) reliability is lower.

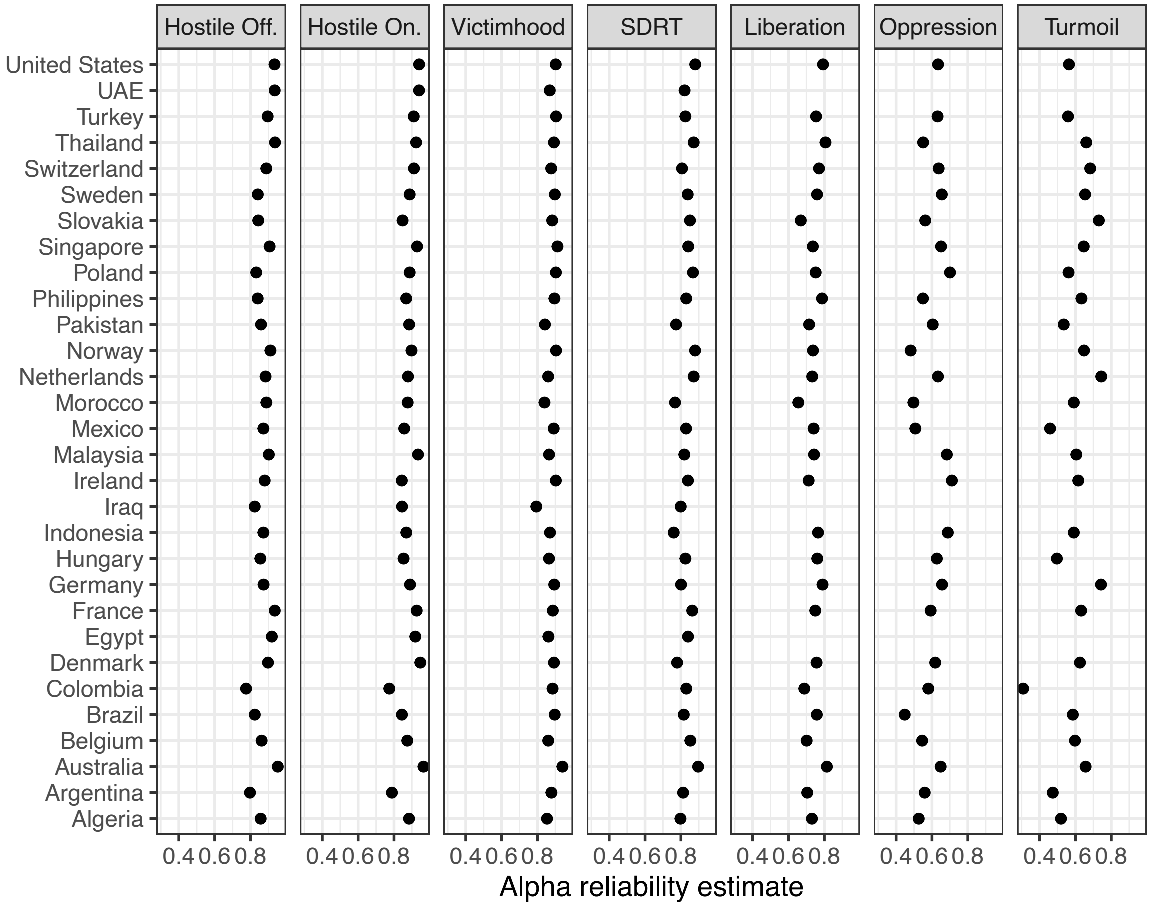

Note: The three variables capturing the perceived impact of social media on politics (Liberation, Oppression, and Turmoil) are omitted from these analyses in the three countries: UAE, Iraq, and Egypt.

Supplementary Fig. 4: Correlation matrix of individual level variables. Estimates are Pearson's correlation coefficients. All variables are country-mean centered.

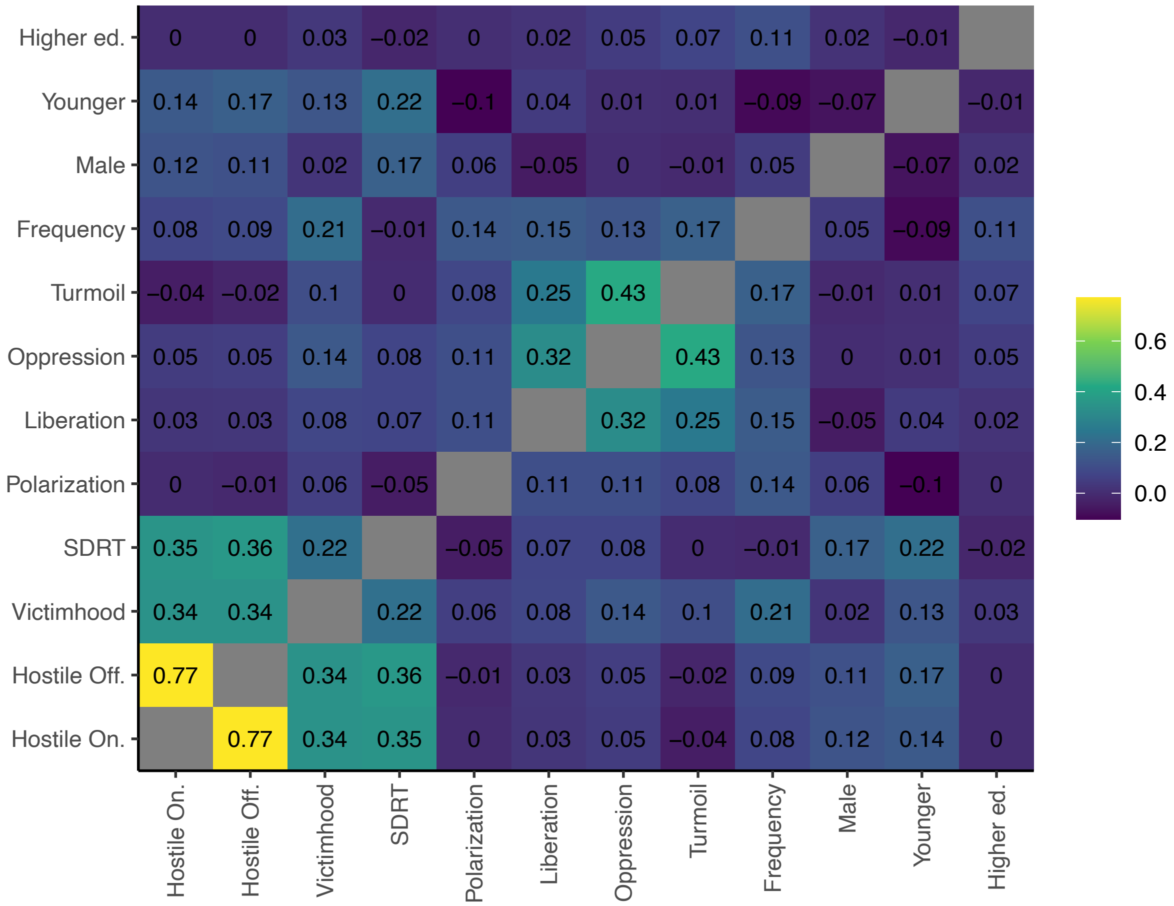

Supplementary Fig. 5: The popularity of Facebook according to the Meta Ad Manager and our samples. Pearson's r = 0.48 (95%CI: 0.14-0.72).

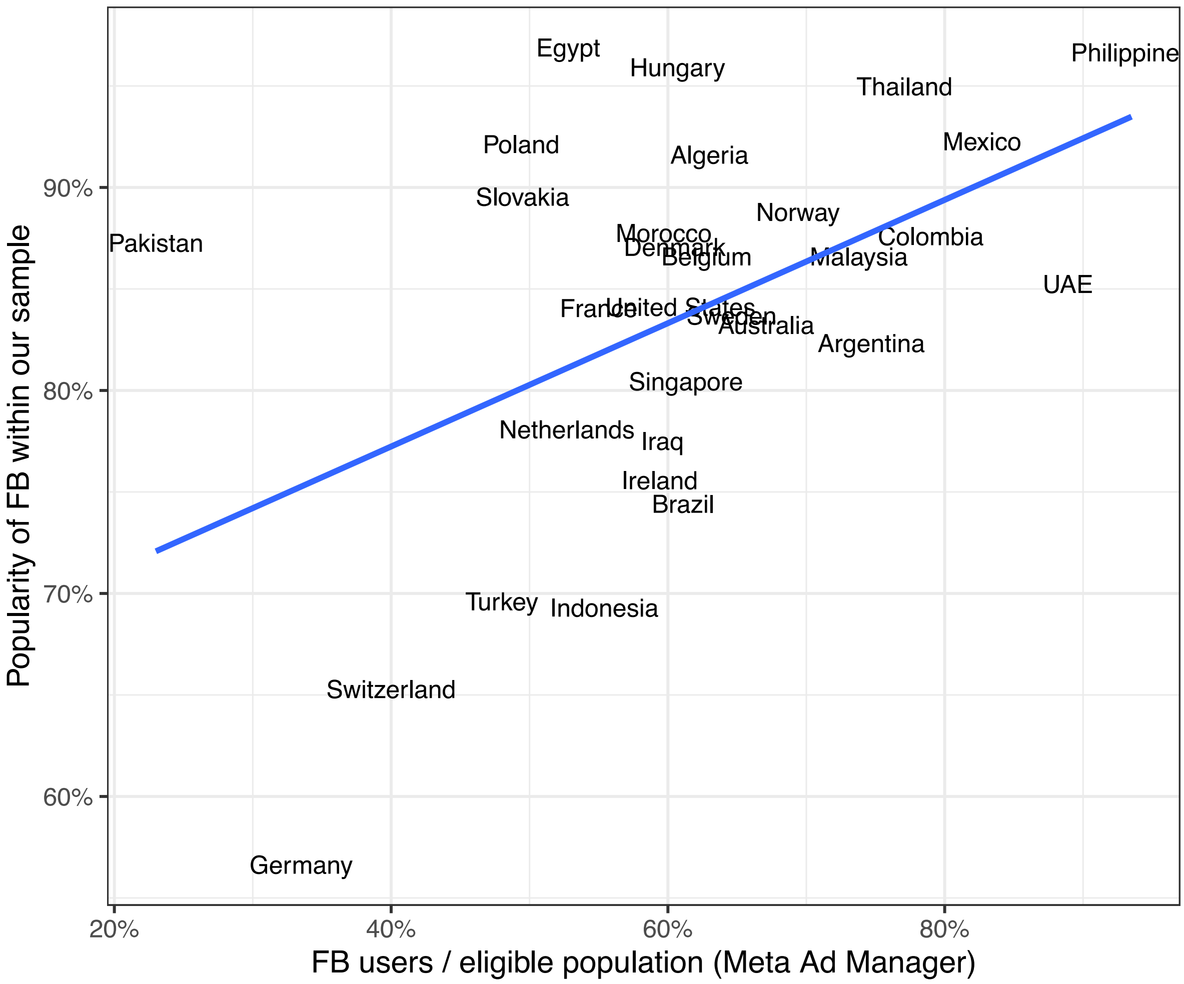

Note: We access data on Facebook user base via https://datareportal.com/ relying on their 2023 country reports (x-axis). We calculate the proportion of respondents in each country sample who says they use Facebook at least occasionally (y-axis).

B Overview of Hypotheses

Supplementary Table 2: Overview of our seven hypotheses. Note that we changed the order and numbering, but not the content of our hypotheses compared to the preregistration.

Original Number	Updated Number	Descriptive Prediction	Concl.
H1	H4	People who report more (vs less) offline hostility also report more online hostility. Specifically, we predict that the standardized beta will be larger than 0.5.	Accept
H2	H5	People higher (vs lower) on status-driven risk taking are more hostile online. Specifically, we predict that the standardized beta will be larger than 0.1.	Accept
H3	H6	People who feel more (vs less) meaninglessness are more hostile online. Specifically, we predict that the standardized beta will be larger than 0.1.	Reject
H4	H7	The association between online hostility and status-driven risk taking is larger among individuals who feel more (vs less) meaninglessness. Specifically, we predict that the standardized beta (of the interaction term) will be larger than 0.	Reject
H5	H2	Countries with more (vs less) economic inequality have more online hostility.	Accept
H6	H3	Countries with more (vs less) poverty have more online hostility.	Accept
H7	H1	Countries with less (vs more) liberal democracy have more online hostility.	Accept

C Follow-up studies assessing the robustness of our hostility measures

We ran a series of follow-up studies on a U.S. nationally representative sample (N = 4294) meant to alleviate concerns about the robustness of our key measures of hostility victimization and perpetration. Table 3 reports the basic demographic details from each study.

Supplementary Table 3: Demographics from our four followup studies

Study	N	Median Age	Share of Women	Share of Higher Ed.
1	1293	44	0.54	0.39
2	849	43	0.5	0.41
3	864	45	0.44	0.41
4	1288	46	0.52	0.38

Follow-up Study 1: Is our measure of online hostility victimization sensitive to who is targeted?

Follow-up Study 1(N = 1293) was intended to assess the extent to which our measure of hostility victimization is sensitive to whether the attacks target the respondent's ingroup vs. them personally vs. anyone online. It proceeded by experimentally manipulating the formulation of the battery of items used to measure hostility victimization in the main cross-cultural study: hostility targeting the agent (ego) vs. the groups the agent identifies with (ingroup) vs. anyone online.

Procedure and materials

After giving informed consent, respondents were randomly allocated to a page asking them to answer the following items. The wording of items varied by condition, presented in a between-subject design to about 33% of respondents.

Please think about the past 30 days, specifically. How often did the following happen to you in discussions about societal issues that occur **on the Internet, such as on social media or in comments sections**?

[Condition in which the hostility target is the ingroup]

1. I saw content that ridiculed people like me.
2. People cursed someone like me out in a discussion.
3. I saw a comment that someone like me would find hurtful.
4. I saw content which was humiliating people like me.
5. I saw content which was threatening or harassing someone like me.

[Condition in which the hostility target is the ego]

1. I saw content that ridiculed me.
2. People cursed me out in a discussion.
3. I saw a comment that I found personally hurtful.
4. I saw content which was humiliating to me.
5. I saw content which was threatening or harassing me.

[Condition in which the hostility target is anyone online]

1. I saw content that ridiculed someone.
2. People cursed someone out in a discussion.
3. I saw a comment hurtful to someone.
4. I saw content humiliating someone.
5. I saw content threatening or harassing someone.

Response options were the same in all conditions and the same as in the battery used in the main study: 1. Never 2. Once a month 3. 2-3 times a month 4. Once or twice a week 5. Multiple times a week

Results

Our findings, presented in Table 4, show that using the personal victimhood measure reduces average group-based victimhood by half a standard deviation, while the general victimhood measure increases it by nearly the same amount (38% of a standard deviation). Regression estimates are reported as Cohen's d-scores. This suggests that our measure primarily captures group-based victimization, clearly distinguishing it from both general perceptions of online hostility and direct personal victimization.

Supplementary Table 4: Group-based victimhood is distinct from both personal and general victimhood, N = 1293

	estimate	95% CI	statistic	p.value
(Intercept)	0.034	-0.054; 0.122	0.754	0.451
General	0.382	0.258; 0.506	6.033	<0.001
Personal	-0.503	-0.628; -0.378	-7.880	<0.001

Follow-up Study 2: Is our measure of hostility perpetration subject to desirability bias?

Follow-up Study 2 (N = 849) gauged the extent to which our measure of hostility perpetration was potentially biased by respondents under-reporting hostile behaviors out of social desirability concerns. We also aimed to see if the correlation between status-driven risk taking and online hostility would be modulated by hypothetical demand bias.

Procedure and materials

After offering informed consent, respondents answered questions measuring their proneness to status-driven risk taking. Then, they were randomly exposed to one of two conditions in a between-subjects design, inspired from Blair and colleagues[1], who developed a method for reducing social desirability in responses. The treatment condition invited respondents to play a digital dice rolling game before asking them about the frequency at which they had engaged in hostile online behavior, and been the targets of online hostility. The control condition just asked respondents how often they had behaved in a hostile way online, and how often they had been victims of hostility, over the last 30 days, without any mention of a digital dice. Contrary to the main study, to reduce study length, hostility perpetration and victimization were captured with 1 item each (presented in a random order).

In the relevant condition, the rolling dice game begins with the following instructions:

For the next questions, I want you to use the widget below to generate a random number between 1 and 6.

- If you get 1, please answer "Never or rarely".
- If you get 6, please answer "Occasionally or often".

[1]Graeme Blair, Kosuke Imai and Yang-Yang Zhou. Design and analysis of the randomized response technique. Journal of the American Statistical Association, 110(511):1304–1319, 2015.

• But if you get another number, like 2 or 3 or 4 or 5 give your honest answer to the question we will ask you.

Now you can try the widget a few times if you want. Remember, this is a true random number generator operated by a trusted 3rd party service (random.org). The number is visible only to you. There is no deception in this survey.

Now please generate a new random number! Remember,

• If you get 1, please answer "Never or rarely".

• If you get 6, please answer "Occasionally or often".

• But if you get another number, like 2 or 3 or 4 or 5, give your honest answer to the question below.

[a button is displayed which activates the random number generator from random.org]

Please think about the past 30 days, specifically. How often did the following happen to you in discussions about societal issues that occur **on the Internet, such as on social media or in comments sections?**

• I saw content which was threatening or harassing someone like me.

Response options: 1. Never or rarely 2. Occasionally or often

Now please generate a new random number! Remember,

• If you get 1, please answer "Never or rarely".

• If you get 6, please answer "Occasionally or often".

• But if you get another number, like 2 or 3 or 4 or 5, give your honest answer to the question below.

[a button is displayed which activates the random number generator from random.org]

• I made a comment that others may find hurtful.

Response options: 1. Never or rarely 2. Occasionally or often

How desirability bias is estimated

Since participants know they may be forced to answer "Never or rarely" or "Occasionally or often" based on the die roll, they can honestly answer without fear of judgment. This randomness means the participant doesn't have to feel judged for their response because, even if they give a socially undesirable answer, they can always say it was just due to the dice roll.

As a result, participants are more likely to answer honestly when the dice directs them to do so (i.e., when they roll 2-5), because their specific answer isn't tied directly to their true opinion. No one knows if their "Yes" was based on truth or on a roll of "1."

How is desirability bias quantified? We know that, on average, one-sixth of the participants will be forced to say "Never or rarely" (roll of 1), and one-sixth will be forced to say "Occasionally or often" (roll of 6), purely due to the dice. The other two-thirds of the responses will be truthful. We can, therefore, think of the observed proportion of "Occasionally or often" responses, as a weighted average of forced and honest answers. Algebraically, this is $p = \frac{1}{3} \times 0.5 + \frac{2}{3}\pi$, where p is the observed proportion of respondents answering "occasionally or often", and π is the proportion of honest answers. Solving for π, we can recover the proportion of honest answers by $\pi = \frac{3}{2}p - \frac{1}{4}$

Results

We compare mean self-reported hostility perpetration and victimization between the forced-response and the standard direct-question approach. Victimization rates do not differ significantly between the direct-question condition (M = 0.36, SD = 0.02) and the forced-response condition (M = 0.34, SD = 0.03), χ^2 (1, N = 705) = 0.120, p = 0.729. Similarly, hostility perpetration show no significant difference between the direct-question condition (M = 0.16, SD = 0.03) and the forced-response condition (M = 0.15, SD = 0.02), χ^2 (1, N = 705) = 0.001, p = 0.969, using Pearson's Chi-squared test with Yates' continuity correction. These results indicate that social desirability bias does not meaningfully affect self-reported hostility perpetration or victimization.

Follow-up Study 3: Are perceptions of hostility victimization subject to desensitization over time?

Follow-up study 3 (N = 864) examined the possibility that experiences of repeated exposure to online hostility might desensitize respondents after a while, thus potentially biasing our measure of hostility victimization.

Procedure and materials

This experiment uses an established design for studying the causal effects of desensitization to hate speech.[2] After giving informed consent, respondents indicated their agreement to the battery of items measuring status-driven risk taking. On the next survey page, they encountered a message inviting them to rate the political leaning of 24 tweets.

> On the next pages, we will present you with a number of posts from social media. We would like to ask you to evaluate the partisan leaning of each post. By "partisan leaning," we mean whether the post seems to favor the Democratic Party, the Republican Party, or neither. In total, we will show you 24 of such posts. Please proceed to the next page to begin.

Respondents where then randomly assigned to one of two conditions in a between-subjects design. In the "civil posts" condition, they saw 24 non-hostile tweets on distinct pages; in the "hostile posts" condition, they read 24 tweets which were explicitly hostile in content on distinct pages. They were asked to rate the perceived partisan leaning of each tweet on a page with the following layout:

How would you rate the partisan leaning of this post?

[Tweet displayed]

Response options:

1. Strongly favors the Democratic Party
2. Somewhat favors the Democratic Party
3. Neutral or no clear partisan leaning
4. Somewhat favors the Republican Party
5. Strongly favors the Republican Party

The goal of the questions about the posts' partisan slant was to get participants to process the tweets' meaning carefully. We used exposure to the 24 hostile vs. civil posts as a manipulation of the level of online hostility participants got exposed to. On the page following exposure to the 24th post, we asked respondents how often they had been the victims of online political hostility over the last 30 days, as per our measure in the main study. The analysis of Follow-up

[2] Soral, W., Bilewicz, M., & Winiewski, M. (2018). Exposure to hate speech increases prejudice through desensitization. *Aggressive behavior*, *44*(2), 136-146.

Study 3 tested whether exposure to the hostile tweets (vs. exposure to the civil tweets) affected respondents' perceptions of the amount of ambient hostility online.

Results

Table 5 presents differences in self-reported victimhood and hostility perpetration comparing the hostile to the civil condition, with regression estimates reported as Cohen's d-scores. Our analysis finds no significant differences between the two conditions. Exposure to a series of hostile messages does not reduce individuals' perceptions of victimhood. If anything, it leads to a slight, but statistically insignificant, increase. Similarly, participants do not become more or less likely to report their own hostile behavior after being exposed to multiple hostile messages. These findings suggest that repeated exposure to hostility does not systematically change our measures of victimhood or hostility perpetration.

Supplementary Table 5: No evidence of desensitization in self-reported hostility or victimhood. N = 864.

DV	term	estimate	95% CI	statistic	p.value
Victim	(Intercept)	-0.058	-0.153; 0.037	-1.204	0.229
Victim	Hostile	0.074	-0.059; 0.206	1.087	0.277
Perpetrator	(Intercept)	-0.039	-0.136; 0.058	-0.783	0.434
Perpetrator	Hostile	0.097	-0.039; 0.233	1.394	0.164

Follow-up Study 4: Are the estimates of the frequency of hostility victimization reliable?

Follow-up Study 4 (N = 1288) consisted in examining the possibility that respondents might have difficulty recollecting the frequency with which they had been the victims of online political hostility, thereby giving superficial and unstable estimates. It used experimentally manipulated anchors (i.e., primes or suggestions) about the amount of hostility encountered online by U.S. citizens, to see if the anchors would affect respondents' own estimates compared to a control condition with no anchor.

Procedure and materials

After giving informed consent, respondents encountered one of three versions of the following vignette and battery of items (each version was exposed to about 33% of participants, in a between-subjects design):

There is currently a lot of worry about hostile content on social media. We would like to know about your experiences. [Low frequency condition: However, a recent survey found that **about 50% of American social media users "never" see hostile content on social media**] [High frequency condition: **Indeed, the majority of American social media users see hostile content on social media every month.**] [Control condition: no information] We would like to know how that compares to your experiences.

Please think about the past 30 days, specifically. How often did the following happen to you in discussions about societal issues that occur **on the Internet, such as on social media or in comments sections?**

1. I saw content that ridiculed people like me. 2. People cursed someone like me out in a discussion. 3. I saw a comment that I found personally hurtful. 4. I saw content which was humiliating people like me. 5. I saw content which was threatening or harassing someone like me.

Response options: 1. Never 2. Once a month 3. 2-3 times a month 4. Once or twice a week 5. Multiple times a week

The analysis of Follow-up Study 4 consisted in testing whether the anchors, compared to their absence, significantly affected estimates of the frequency of exposure to online hostility.

Results

Table 6 presents regression estimates as Cohen's d-scores, comparing the low and high anchor conditions to the control condition. Our results show that introducing the low anchor has no significant or meaningful effect on self-reported victimization. In contrast, the high anchor increases victimization by a quarter of a standard deviation.

These findings have at least two key implications. First, they suggest that individuals in the U.S. generally assume lower levels of online hostility unless prompted with information indicating a high prevalence. Second, they indicate that perceptions of online victimization

are influenced by external narratives, but primarily by those emphasizing high levels of online hostility. This implies that our measurement may be shaped by national discourse if discussions about the prevalence of online hostility systematically vary across countries. While our data do not allow us to precisely estimate the resulting bias, they do indicate its likely direction. Specifically, as shown in Online Appendix L, more democratic countries—where self-reported victimization is lower—tend to have *stronger* narratives portraying social media as a source of turmoil. Insofar as turmoil narratives inflate self-reports, perceptions in more democratic countries are likely upwardly biased. In contrast, less democratic countries—despite reporting higher levels of victimization—exhibit weaker turmoil narratives, suggesting their self-reports are less inflated. We would expect more bias in countries with little hostility, and less bias in countries with more hostility. Following this logic, our estimates of cross-country differences in victimization are likely conservative; the true disparities may be even larger than observed.

Supplementary Table 6: Estimating anchoring in self-reported online victimhood. N = 1,288

term	estimate	95% CI	statistic	p.value
(Intercept)	-0.093	-0.187; 0.001	-1.947	0.052
Low	0.007	-0.127; 0.141	0.103	0.918
High	0.243	0.111; 0.376	3.593	<0.001

D Notable socio-political events during data collection

Considering that our survey-based observational data only represents a snapshot of a specific country in a specific time period, our country-level estimates need to be contextualized. Certain socio-political events in a particular country during the data collection period may have influenced country-level estimates.[3] Table 7 thus provides an overview of the period of data collection in each country, including notable socio-political events that occurred during, 30 days before or shortly after data collection (as we ask participants to think of the last 30 days when reporting experiences or perpetration of hostility, we focus primarily on the period between the beginning of the first month and the end of the data collection period. However, notable events immediately after data collection are also included as these may also have influenced the measures and perceptions prior to the actual event happening e.g. election campaigning during an election that took place shortly after data collection). Considering the notable political events in Brazil and France at the start of 2023, which might still be influencing estimates during data collection, we included these events even though they fall outside the specified time period. This decision was undertaken to acknowledge the potentially substantial impact of these very significant events on the overall political situation and therefore also people's perceptions in these countries. To identify socio-political events in the given time-periods, we have consulted Wikipedia pages for each given country on which events are compiled for each year, month, and day. Please find links to the Wikipedia pages for the individual countries in the under the hyperlinks below (last accessed 2024-03-06).

We believe events like these do not threaten the validity of our estimates of macro-level correlations, for two reasons. First, these events should be at least partly endogenous to the more stable macro indicators of liberal democracy and economic inequality. For example, it is plausible that we found Turkey to be the most hostile country in our sample in part because we happened to collect data during the controversial Presidential election. But the fact that this election may have triggered a lot of hostility is party because Turkey is an undemocratic, economically unequal country. Second, random events may also bias our estimates of the level of hostility, but as with measurement error in general, these would bias our estimated associations downwards, making our estimates more conservative.

[3] Stig Hebbelstrup Rye Rasmussen and Michael Bang Petersen. The event-driven nature of online political hostility: How offline political events make online interactions more hostile. PNAS nexus, 2(11):pgad382, 2023.

Supplementary Table 7: Data collection period by country and notable socio-political events. All dates refer to events in 2023.

Country	Start	End	Notable events
Algeria	05-25	06-20	no major political events
Argentina	05-10	05-21	no major political events
Australia	04-14	05-03	May 7: Anti-crime rally in Queensland descending into chaos
Belgium	05-22	05-28	no major political events
Brazil	05-10	05-17	no major political events
Colombia	05-17	05-24	no major political events
Denmark	04-17	04-20	no major political events
Egypt	05-25	06-21	no major political events
France	05-10	05-24	no major political events
Germany	04-17	04-20	no major political events
Hungary	04-25	05-06	April 24: protests against new education law, May 3: student protests
Indonesia	05-25	06-01	no major political events
Iraq	05-25	07-07	no major political events
Ireland	04-14	04-19	April 10: 25th anniversary of Good Friday Agreement, April 10-14: official visit by Joe Biden
Malaysia	05-22	05-30	no major political events
Mexico	05-10	05-24	no major political events
Morocco	05-25	06-15	no major political events
Netherlands	05-17	05-30	May 30: Dutch Senate Election, July 7: Dutch cabinet collapses because of disagreement over migration law
Norway	04-17	04-21	April 17-20: strikes due to trade union negotiations
Pakistan	06-02	06-20	no major political events
Philippines	05-22	05-30	no major political events
Poland	05-10	05-16	June 4: Anti-government protests in Warsaw
Singapore	04-14	04-20	no major political events
Slovakia	05-10	05-16	May 7: President Čaputová appoints technocratic cabinet due to long-term political crisis
Sweden	04-17	04-20	March 22: Swedish Parliament approves Sweden's application for NATO membership, Quran burnings throughout 2023
Switzerland	05-16	05-23	no major political events
Thailand	05-26	06-13	May 14: Thai general election
Turkey	05-10	05-24	May 14: Parliamentary election and first round of presidential election
UAE	05-25	06-12	no major political events
USA	04-14	04-20	March 30: First indictment of Donald Trump by Manhattan grand jury

E Supplementary results about the macro-level estimates and correlations of online hostility

In this section, we report supplementary details and evidence for results mentioned in the main text (and if not otherwise noted, planned in the preregistration). Specifically, Tables 8, 9, and 10 report the country level estimates visualized in Figures 1, 2, and 3, respectively. Figure 6 demonstrates the robustness of macro correlations when including other focal macro-predictors. Figure 7 serves as an aid to interpret strengths of associations relying on a commonly used metric: Spearman's rank-order correlation coefficient. Given that Spearman's rho is based on ranks, its sampling distribution is non-normal, especially in small samples, and hence estimating confidence intervals around them is non-trivial. Readers interested in the uncertainty of our estimates should refer to our original Bayesian estimates.

Country-level estimates of online political victimhood from our main model as visualized in Figure 1

Supplementary Table 8: Average level of political victimhood by country (and 89% CI)

country	Estimate	CI.lwr	CI.upr
Algeria	0.3	0.28	0.32
Argentina	0.37	0.35	0.39
Australia	0.23	0.21	0.24
Belgium	0.24	0.22	0.26
Brazil	0.37	0.35	0.39
Colombia	0.34	0.32	0.36
Denmark	0.17	0.15	0.19
Egypt	0.33	0.31	0.35
France	0.21	0.19	0.23
Germany	0.21	0.19	0.23
Hungary	0.29	0.27	0.31
Indonesia	0.31	0.29	0.33
Iraq	0.31	0.29	0.33
Ireland	0.25	0.23	0.27
Malaysia	0.34	0.32	0.36
Mexico	0.34	0.32	0.36
Morocco	0.33	0.31	0.35
Netherlands	0.23	0.21	0.25
Norway	0.21	0.19	0.23
Pakistan	0.38	0.36	0.4
Philippines	0.36	0.34	0.38
Poland	0.3	0.28	0.32
Singapore	0.19	0.17	0.21
Slovakia	0.3	0.28	0.32
Sweden	0.2	0.18	0.22
Switzerland	0.22	0.2	0.24
Thailand	0.33	0.31	0.35
Turkey	0.46	0.44	0.48
United Arab Emirates	0.37	0.35	0.39
United States	0.33	0.3	0.34

Country-level estimates of the relationship between online and offline hostility in political discussions as reported in Figure 2

Supplementary Table 9: Average association (and 89% CI) between online and offline political hostility by country

country	Estimate	CI.lwr	CI.upr
Brazil	0.67	0.63	0.72
Colombia	0.68	0.64	0.72
Argentina	0.68	0.64	0.72
Hungary	0.69	0.64	0.73
Slovakia	0.69	0.65	0.73
Mexico	0.71	0.66	0.75
Singapore	0.72	0.67	0.76
Algeria	0.73	0.68	0.78
Denmark	0.73	0.69	0.77
Thailand	0.73	0.69	0.78
Philippines	0.73	0.69	0.78
Morocco	0.73	0.69	0.78
Ireland	0.73	0.69	0.77
Indonesia	0.75	0.71	0.8
Malaysia	0.75	0.71	0.8
Pakistan	0.77	0.73	0.81
Germany	0.78	0.73	0.82
Norway	0.78	0.73	0.82
Iraq	0.78	0.73	0.82
Poland	0.78	0.73	0.82
Belgium	0.78	0.74	0.82
Turkey	0.79	0.74	0.83
Egypt	0.81	0.77	0.85
France	0.83	0.78	0.87
Sweden	0.83	0.78	0.87
Switzerland	0.84	0.79	0.88
United.Arab.Emirates	0.85	0.81	0.9
United.States	0.86	0.81	0.9
Netherlands	0.89	0.85	0.93
Australia	0.9	0.85	0.94

Country-level estimates of the relationship between online political hostility and status-driven risk taking as reported in Figure 3

Supplementary Table 10: Average association (and 89% CI) between online political hostility and status-driven risk taking by country

country	Estimate	CI.lwr	CI.upr
Iraq	0.2	0.14	0.27
Singapore	0.24	0.18	0.3
Colombia	0.24	0.18	0.3
Philippines	0.24	0.19	0.31
Slovakia	0.25	0.19	0.31
Indonesia	0.25	0.19	0.31
Hungary	0.26	0.2	0.32
Argentina	0.26	0.2	0.32
Algeria	0.26	0.2	0.33
Mexico	0.27	0.21	0.33
Malaysia	0.27	0.22	0.33
Poland	0.29	0.23	0.34
Brazil	0.29	0.23	0.35
Pakistan	0.3	0.23	0.36
Ireland	0.3	0.25	0.37
United.Arab.Emirates	0.31	0.24	0.37
Thailand	0.31	0.25	0.37
Belgium	0.32	0.26	0.37
Morocco	0.36	0.3	0.42
Turkey	0.36	0.3	0.42
Denmark	0.36	0.3	0.42
Egypt	0.38	0.31	0.44
Sweden	0.38	0.32	0.44
Germany	0.39	0.33	0.45
Switzerland	0.39	0.33	0.45
United.States	0.41	0.35	0.47
France	0.41	0.35	0.48
Norway	0.41	0.35	0.48
Australia	0.46	0.4	0.52
Netherlands	0.51	0.45	0.57

Coefficients from regressing victimhood on democracy, inequality, and poverty

Supplementary Fig. 6: Democracy, and income inequality are robust predictors of country-level average online political victimhood. Poorer countries also have more victimhood, but this correlation disappears once we add both democracy and inequality to the regressions.

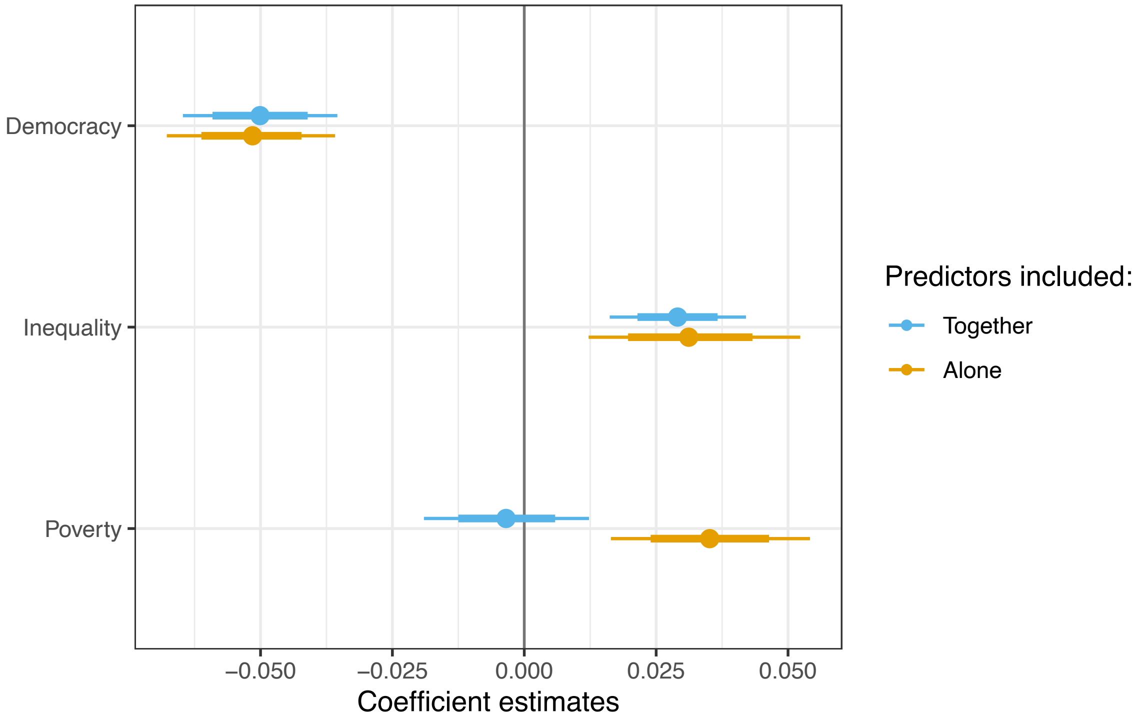

Note: Estimates are based on multilevel bayesian regression models with weakly informative priors. Coefficients correspond to the predicted difference in average online political victimhood for a one-standard-deviation difference in democracy, inequality, or poverty. Errorbars denote 67% and 89% credible intervals.

Polarization

In our preregistration, we also planned to explore if country level polarization of society (as measured by V-Dem institute's expert ratings "v2cacamps") correlates positively with victimhood of political hostility. Running a regression model identical to those reported above, we find that indeed, in countries with higher political polarization, respondents fall victim to online political hostility more often, $\beta = 0.04$. 89% CI [0.03; 0.06].

Supplementary Fig. 7: Correlation matrix of country-level variables and estimates. Estimates are rank-order correlation coefficients (Spearman's rho).

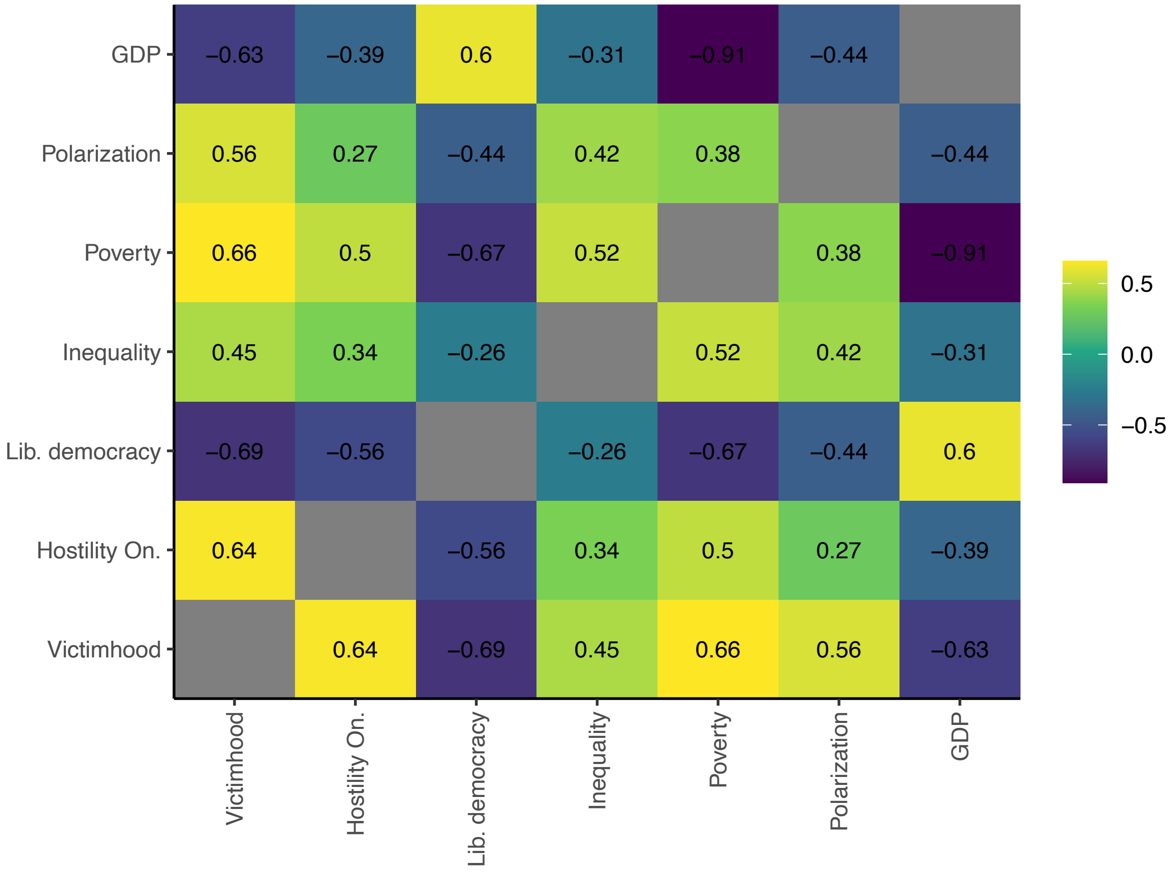

F Platform differences in online political victimhood across countries

It is of obvious importance to ask how often users are victimized across various platforms. Unfortunately, our data is not very well suited to answer this question, because we have not collected data on users' platform-specific experiences or behaviors. We did collect data on which platforms respondents use, and how often they are victimized in conversations about social issues. We can therefore regress our victimhood-index on a series of dummy variables for each of the major social media platforms: Twitter, Tiktok, Telegram, Snapchat, Instagram, Youtube, Facebook, and Whatsapp. Our model includes intercepts and effects varying by country. The interpretation of our resulting coefficients is the difference between the average level of victimization among users who are using versus not using a given platform, assuming they use all other platforms similarly. We display our estimates in Figure 8 below.

Supplementary Fig. 8: Victimhood across platforms. The black points and errorbars denote the pooled estimates and 89% credible intervals. Gray points denote country-level estimates.

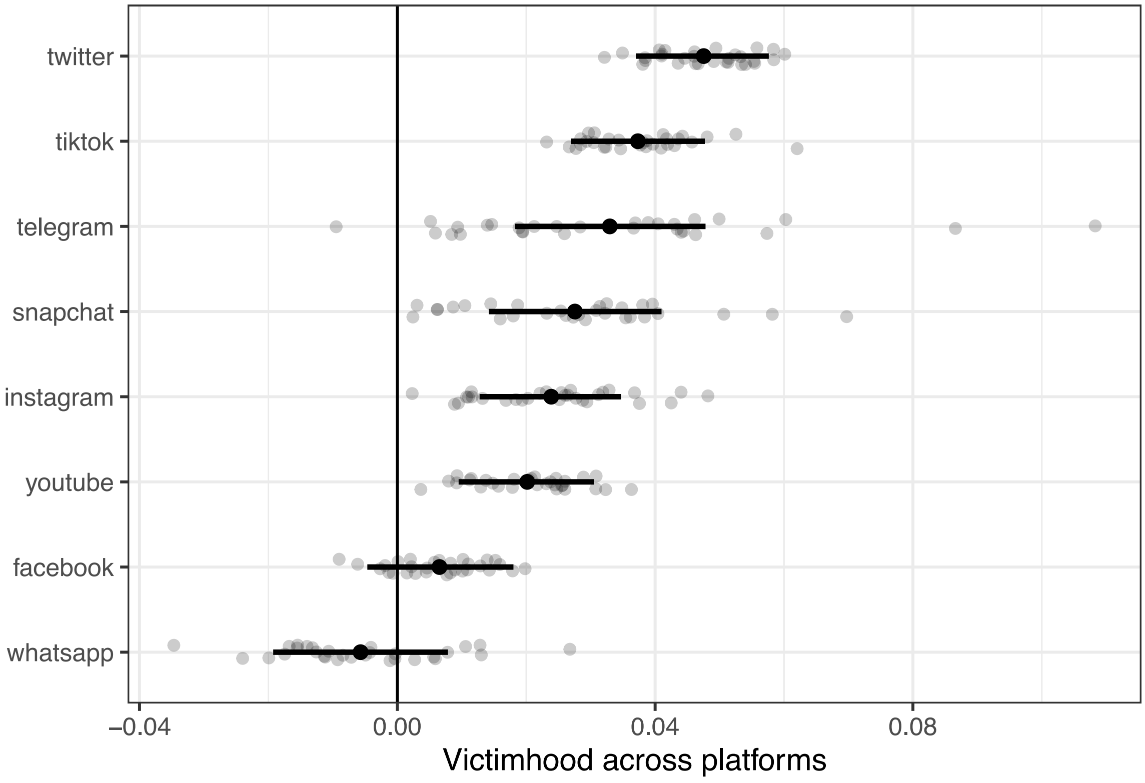

Results show that the differences are generally small. Even the largest coefficient – for Twitter – shows only a difference of 0.05 (or 5 percentage points), amounting to 16% of a standard deviation in respondent-level victimhood. We also see that the estimates are never negative: using any social media platform entails risks of exposure.[4]

With regards to platform differences, Facebook and Whatsapp users are not experiencing any more victimhood than non-users, although we should note that it is unclear how much of this is because these are the two most popular platforms, and thus there is little variance to work with. Next, Instagram and YouTube users are slightly more exposed to hostility as non-users (around 0.02). Snapchat and Telegram users are victimized on average slightly more than non-users, and it is notable that for these two platforms country differences are much larger than for all other platforms. Finally, users of TikTok and Twitter are the most likely to fall victim to online hostility, compared to non-users. However, there are two caveats: 1) as we said above, this difference remains small, and 2) we did not collect data on some of the most notoriously hostile corners of the internet, such as 4chan.

[4]Indeed, we find that victimhood can be modeled well as a single linear function of the number of platforms a respondent uses. Each additional social media platform a respondent uses increases the average victimhood measures by $\beta = 0.03$, 89% CI [0.02; 0.03].

G Exploratory analyses of country-level correlates of online political hostility

Country-level Hostility and Violence

In the main analysis, we have shown that online hostility is predicted by offline hostility. Here we validate these results using country-level indicators of hostility and violence. We used several indicators of violence and hostility from the V-Dem: *Physical Violence*, which is measured by indicators of freedom from political killings and torture by the government.[5] *Political violence* reflects the extent to which non-state actors used political violence against individuals to achieve political goals. *Electoral violence* measures the extent to which violence between civilians occurred in the context of elections. *Harassment of journalists* reflects whether journalists are harassed by state or non-state actors in their journalistic work. *Hate speech by political parties* measures how often major political parties use hate speech. Variables are coded from 0-1, with higher values indicating higher levels of violence or hostility. We use Spearmans' rank correlations (ρ) to measure the strength and direction of association.

Figure 9 shows that, on average, self-reported online victimization is higher in countries with more physical violence (ρ = 0.76, p < 0.01), political violence (ρ = 0.51, p < 0.01), electoral violence (ρ= 0.47, p < 0.01), harassment of journalists (ρ = 0.61, p < 0.01), and in countries where political parties use more hate speech (ρ = 0.5, p < 0.01). Self-reported online hostility is on average higher in countries with more physical violence (ρ = 0.52, p < 0.01), electoral violence (ρ = 0.42, p < 0.05), and more harassment of journalists (ρ = 0.48, p < 0.01). The trend is also present in countries with more political violence (ρ = 0.25, p = 0.18) and more hate speech by political parties (ρ = 0.2, p = 0.3), but not statistically significant.

Types of Democracy

We have shown that more liberal democratic countries (as measured by V-Dem's Liberal Democracy Index, version 14) have lower levels of online political hostility than less democratic countries. Is this relationship stable for other types of democracy? Here we offer insights into the relationships between online political hostility and four other conceptualizations of democracy

[5] Seraphine Maerz, Amanda Edgell, Sebastian Hellmeier, and Nina Ilchenko. Vdemdata - an R package to load, explore and work with the most recent V-Dem (Varieties of Democracy) and V-Party datasets, 2021.

Supplementary Fig. 9: Online Political Hostility and Country-level Violence and Hostility. People living in countries with higher levels of violence and hostility fall victim to and perpetrate more online political hostility on average.

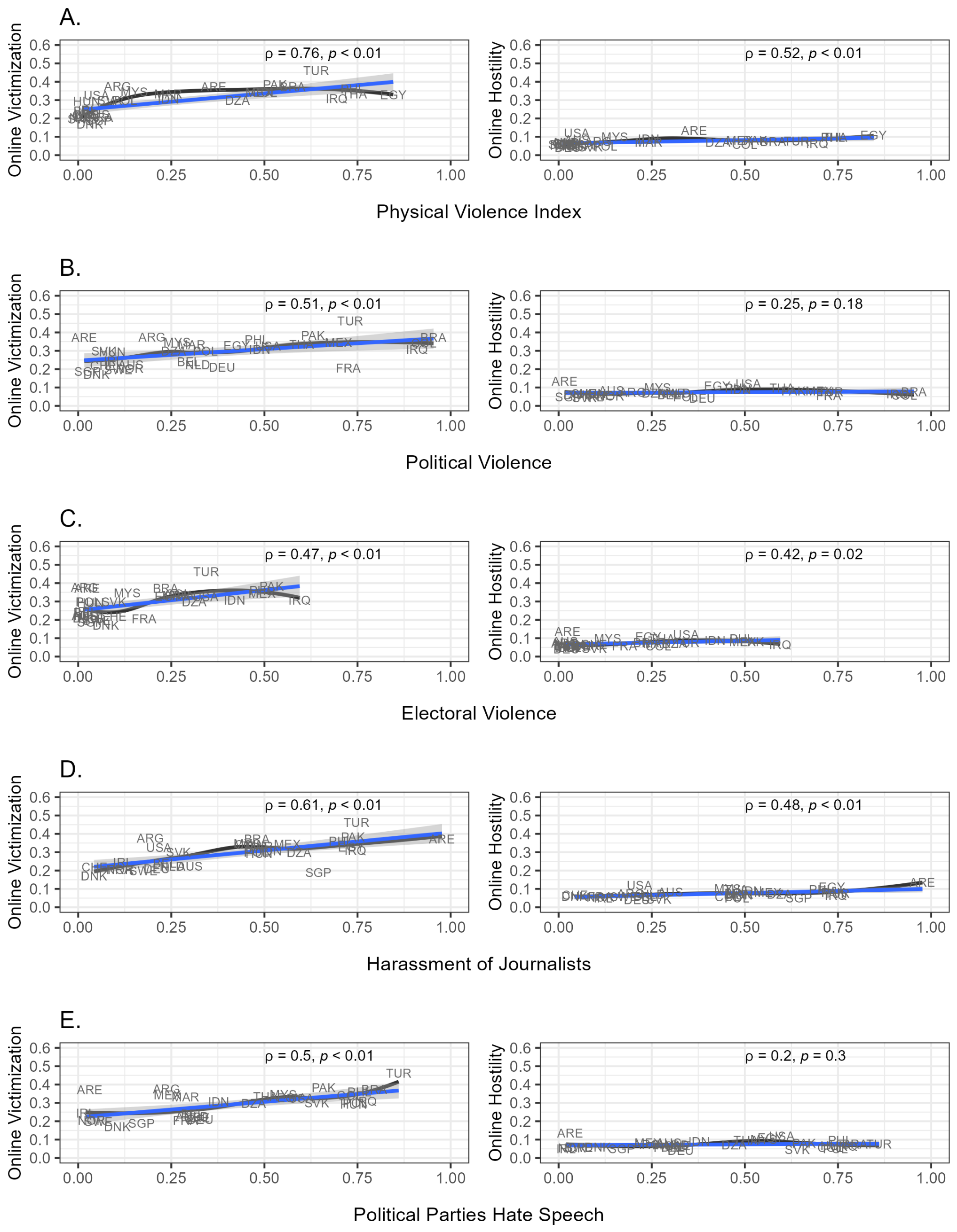

Note: Correlations of online victimization and online hostility with country-level indicators of physical violence (Panel A), political violence (Panel B), electoral violence (Panel C), harassment of journalists (Panel D) and hate speech by political parties (Panel E). Scatterplots show Spearman's rank correlations (ρ) with p-values in the upper right corner, linear trend lines in blue with 95% confidence intervals, and Lowess curves in black

provided by V-Dem: (1) *Electoral Democracy Index*, reflecting the extent to which freedom of association, free and fair elections, freedom of expression, elected officials, and voting rights are present (2) *Participatory Democracy Index*, measuring the engagement of civil society in politics and the presence of direct democracy as well as subnational elected bodies, (3) *Deliberative Democracy Index*, indicating whether political decisions are made through respectful dialog and public argumentation by informed and competent participants who are open to persuasion (4) *Egalitarian Democracy Index*, reflecting whether societal groups have equal rights and freedoms, as well as equal access to resources and political power. The latter three conceptualizations of democracy are all based on the index of electoral democracy and contain additional components that indicate the participatory, deliberative or egalitarian character of democracy respectively. The indices are coded from 0-1, with higher values indicating a higher degree of democracy. We use Spearman's rank correlations (ρ) to measure the strength and direction of the relationships between online political hostility and different types of democracy.

Figure 10 shows that there is a lower average risk of becoming a victim of political hostility online and becoming a perpetrator in more democratic countries than in less democratic countries, regardless of the specification of democracy. More specifically, less online victimization and less online hostility are reported in more electoral democracies (*online victimization:* ρ = -0.63, p $<$ 0.01; *online hostility*: Spearman's ρ = -0.56, p $<$ 0.01), more participatory democracies (*online victimization:* ρ = -0.58, p $<$ 0.01; *online hostility*: ρ = -0.52, p $<$ 0.01), more deliberative democracies (*online victimization:* ρ = -0.66, p $<$ 0.01; *online hostility*: ρ = -0.52, p $<$ 0.01), and more egalitarian democracies (*online victimization:* ρ = -0.71, p $<$ 0.01; *online hostility*: ρ = -0.63, p $<$ 0.01).

Types of Inequality

Consistently with our expectations, we found that people living in countries with greater economic inequality (as measured by the Gini coefficient) are more likely to be victims and perpetrators of online political hostility than people living in economically egalitarian societies. To test the robustness of these findings, we examine the extent to which people of different socio-economic status (SES) are treated equally and how this relates to online political hostility. Instead of measuring economic inequality using income indicators, we measure the extent to

Supplementary Fig. 10: Online Political Hostility and Types of Democracy. People living in countries with higher levels of democracy fall less victim to and perpetrate less online political hostility on average.

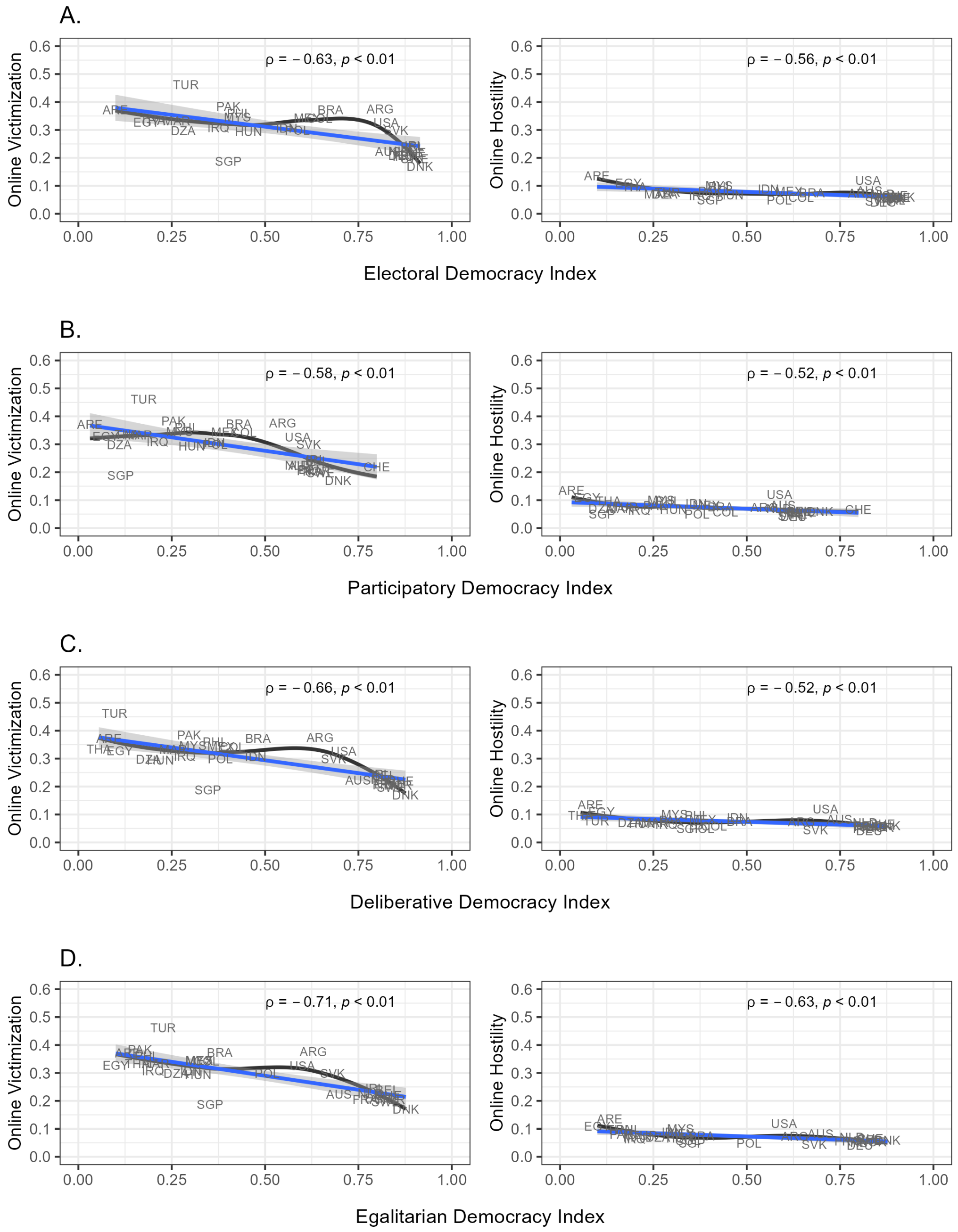

Note: Correlations of online victimization and online hostility with different types and levels of Democracy: Electoral Democracy (Panel A), Participatory Democracy (Panel B), Deliberative Democracy (Panel C), and Egalitarian Democracy (Panel D). Scatterplots show Spearman's rank correlations (ρ) with p-values in the upper right corner, linear trend lines in blue with 95% confidence intervals in grey, and Lowess curves in black

Supplementary Fig. 11: Online Political Hostility and Equality by Socio-economic Status. In countries where people of different socio-economic statuses are treated more equally in politics, people on average report falling less victim to and perpetrating less online hostility.

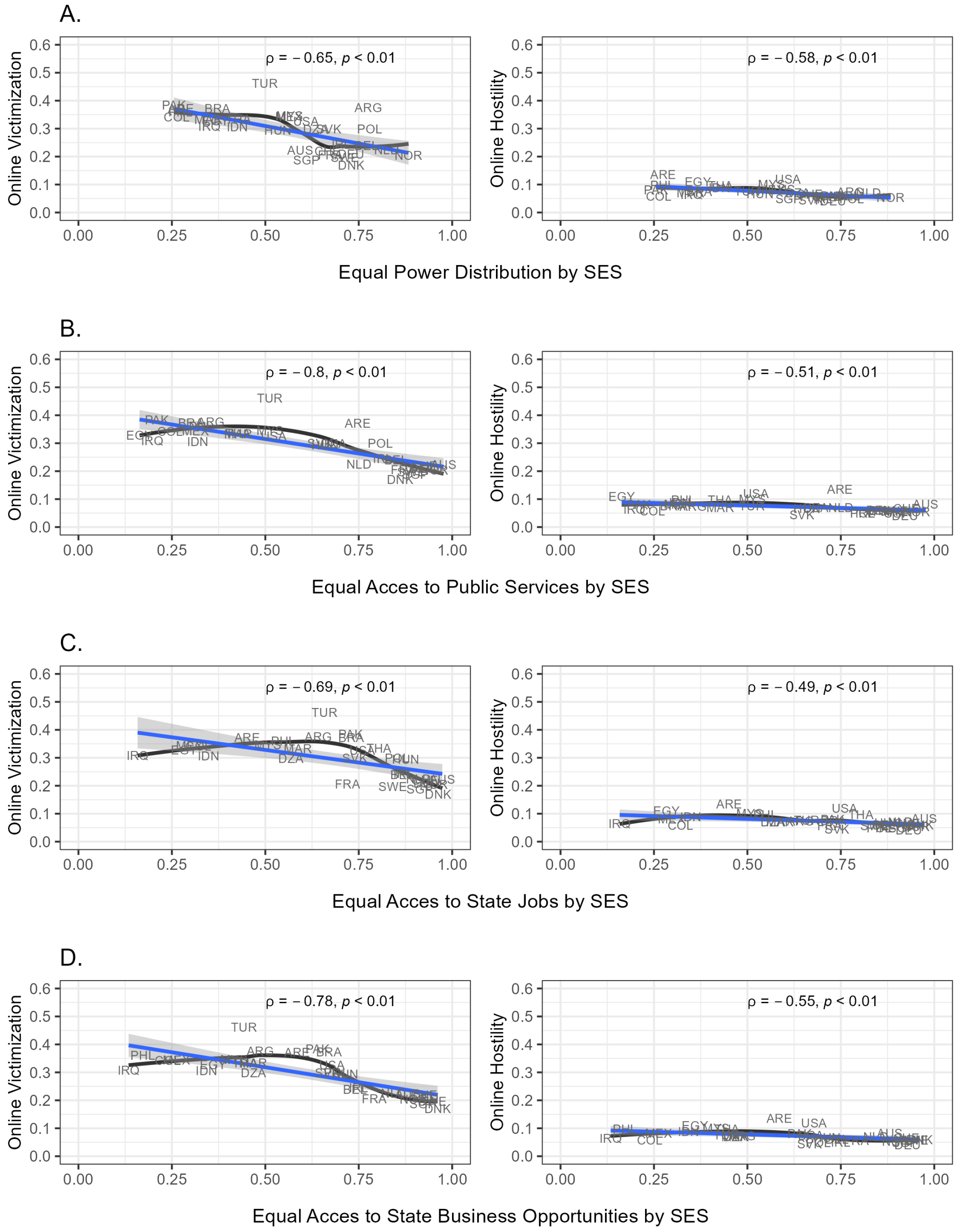

Note: Correlations of online victimization and online hostility with different indicators of country-level equality based on socio-economic status: Equal Power Distribution (Panel A), Equal Access to Public Services (Panel B), Equal Access to State Jobs (Panel C), and Equal Access to State Business Opportunities (Panel D). Scatterplots show Spearman's rank correlations (ρ) with p-values in the upper right corner, linear trend lines in blue with 95% confidence intervals in grey, and Lowess curves in black.

which equality exists between people of different SES defined by characteristics such as wealth, occupation or property ownership. To this end, we use four measures of V-Dem: (1) Equal distribution of political power by SES, (2) Equal access to public services by SES, (3) Equal access to government jobs by SES, (4) Equal access to government business opportunities by SES. Measures are coded from 0-1, with higher scores indicating higher levels of equality. We use Spearman rank correlations (ρ) to measure the strength and direction of relationships between online political hostility and equality across different SES.

Figure 11 shows that more equality between individuals of different SES is associated with less victimhood and less perpetration of online political hostility. More specifically, lower online victimization and lower online hostility are associated with more equal political power distribution by SES (*online victimization:* ρ = -0.65, p < 0.01; *online hostility*: ρ = -0.58, p < 0.01), more equal access to public services (*online victimization:* ρ = -0.8, p < 0.01; *online hostility*: ρ = -0.51, p < 0.01), state jobs (*online victimization:* ρ = -0.69, p < 0.01; *online hostility*: ρ = -0.49, p < 0.01), and state business opportunities (*online victimization:* ρ = -0.78, p < 0.01; *online hostility*: ρ = -0.55, p < 0.01) by SES.

Defamation Protection and Falling Victim Across Liberal Democracies and Economically Unequal Countries

The political and economic conditions of a country may shape the effectiveness and extent of content moderation practices, thereby influencing the likelihood of falling victim to online hostility. To provide some suggestive insights into a possible mechanism like this, we provide exploratory correlative evidence using V-Dem indicators to examine the relationship between levels of liberal democracy, inequality and defamation protection (defined by V-Dem as the degree to which a country's legal framework provides protection against defamatory online content or hate speech). We find a positive correlation (Spearman's rho) between liberal democracy and defamation protection ($\rho = 0.31$, p = 0.09), and a negative correlation between inequality and defamation protection ($\rho = -0.47$, p ¡ 0.01), suggesting that democracies tend to have stronger legal frameworks for addressing harmful content, while more economically unequal countries have less legal frameworks for addressing such harmful content. Going a bit further, we find that controlling for defamation protection reduces the association between both liberal

democracy (by 18%) and inequality (by 52%), see details in Table 11.

Supplementary Table 11: OLS Regression Coefficients on Falling Victim Across Liberal Democracies and Economically Unequal Countries, controlling for Defamation Protection

	Dependent variable:			
	Victimhood			
	(1)	(2)	(3)	(4)
Liberal Democracy Index	−0.18	−0.15		
	(−0.25, −0.11)	(−0.22, −0.08)		
Defamation Protection		−0.16		−0.22
		(−0.29, −0.04)		(−0.39, −0.05)
Inequality (Gini)			0.39	0.19
			(0.07, 0.71)	(−0.15, 0.52)
Constant	0.39	0.49	0.16	0.38
	(0.35, 0.43)	(0.40, 0.57)	(0.04, 0.27)	(0.18, 0.59)
Observations	30	30	30	30
R^2	0.51	0.61	0.19	0.35
Adjusted R^2	0.49	0.58	0.16	0.30

Note: 95% CI in parentheses

H Feelings of meaninglessness do not predict online political hostility

Figure 12 demonstrates that our data and model indicate that feelings of meaninglessness (i.e. anomie) and being hostile in online political conversations are uncorrelated β = -0.01, 89% CI [-0.03; 0.01]. In other words, we find no evidence for the preregistered H3. These associations are substantially smaller than the estimates for status-driven risk taking. Moreover, leave-one-out cross validation demonstrates that the explanatory power of the model with meaninglessness is much weaker than the model with status-driven risk taking (ELPD (SE) of -850.2 (69.9)).

Furthermore, we also find no evidence that there is a meaningful interaction between status-driven risk-taking and meaninglessness (H4 in the preregistration): β = -0.03, 89% CI [-0.05; 0]"

Supplementary Fig. 12: Feelings of meaninglessness are consistently unrelated to being hostile in online political conversations. The figure displays the country-level posterior distribution of standardized beta estimates. The vertical line at 0.1 denotes the effect size we preregistered to test our hypothesis against. We juxtapose meaninglessness estimates to identical estimates for status-driven risk taking (SDRT), highlighting the substantial difference between the two.

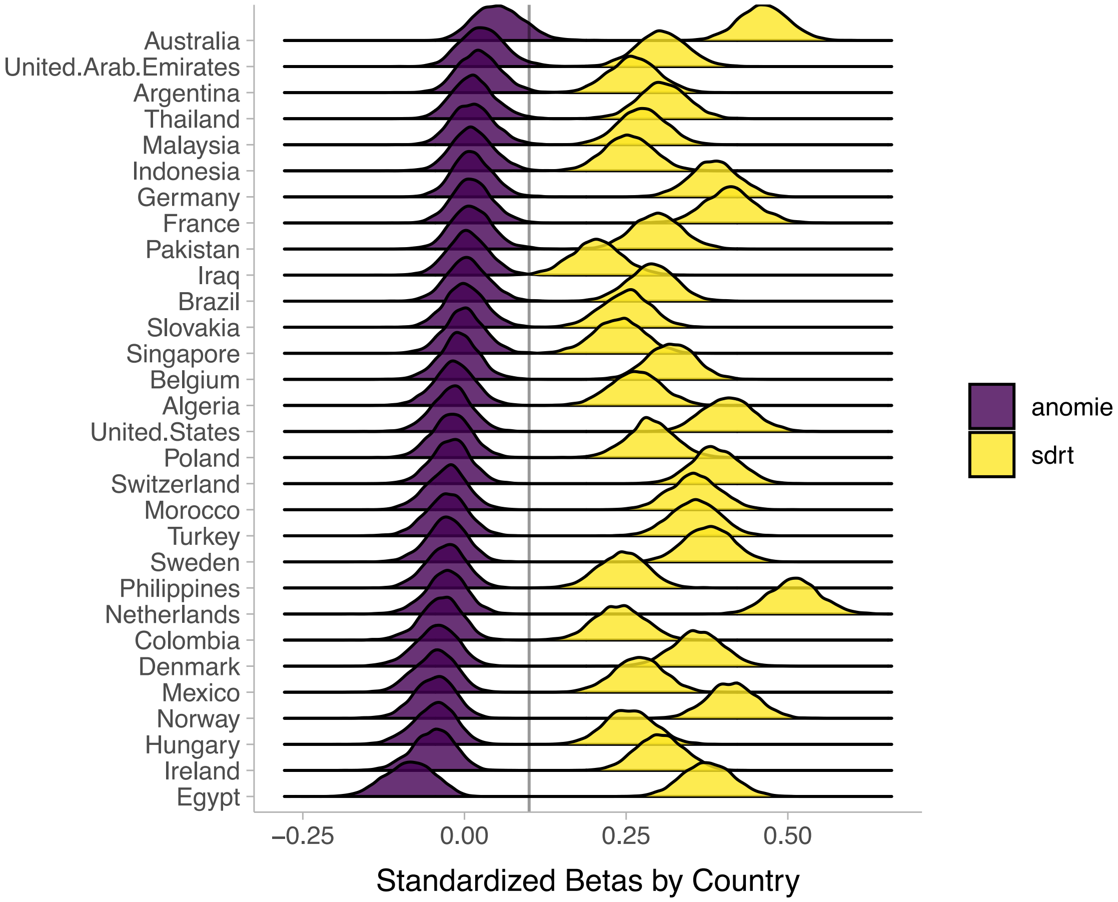

Note: Estimates are based on Bayesian multilevel regressions with weakly informative priors.

I Affective polarization does not predict online political hostility

In addition to status-seeking as a cause of online hostility, we also measured an alternative psychological predictor: affective polarization. We operationalized affective polarization as the absolute difference in positive feelings towards political groups supporting the current government and positive feelings towards groups opposing the government.[6] In-group versus out-group distinctions are central to how individuals treat others, especially in times of conflict. However, the extent to which affective polarization translates into online and offline hostile behavior has until now received no systematic cross-cultural examination.[7]

We predicted that *people who score higher on affective polarization also report more online political hostility* (Hypothesis 8 in the preregistration). Against our prediction, we found no association between affective polarization and being more hostile at the individual level (β = 0.01, 89%CI [-0.01; 0.02]). Again, these associations are substantially smaller than the estimates for status-driven risk taking. Moreover, leave-one-out cross validation demonstrates that the explanatory power of the model with affective polarization is much weaker than the model with status-driven risk taking (ELPD (SE) of -728.9 (64.4)).

This result contradicts the country-level result described in OA Section E that countries that experts deem more polarized have more online political hostility. It is plausible that online hostility is one of the factors which experts pick up on when judging the polarization of a country.

[6] Iyengar, S., & Westwood, S. J. (2015). Fear and loathing across party lines: New evidence on group polarization. American journal of political science, 59(3), 690-707.

[7] Boxell, L., Gentzkow, M., & Shapiro, J. M. (2017). Greater Internet use is not associated with faster growth in political polarization among US demographic groups. Proceedings of the National Academy of Sciences, 114(40), 10612-10617. Tappin, B. M., & McKay, R. T. (2019). Moral polarization and out-party hostility in the US political context. Journal of Social and Political Psychology, 7(1), 213-245.

Supplementary Fig. 13: Affective polarization is consistently unrelated to being hostile in online political conversations. The figure displays the country-level posterior distribution of standardized beta estimates. The vertical line at 0.1 denotes the effect size we preregistered to test our hypothesis against. We juxtapose polarization estimates to identical estimates for status-driven risk taking (SDRT), highlighting the substantial difference between the two.

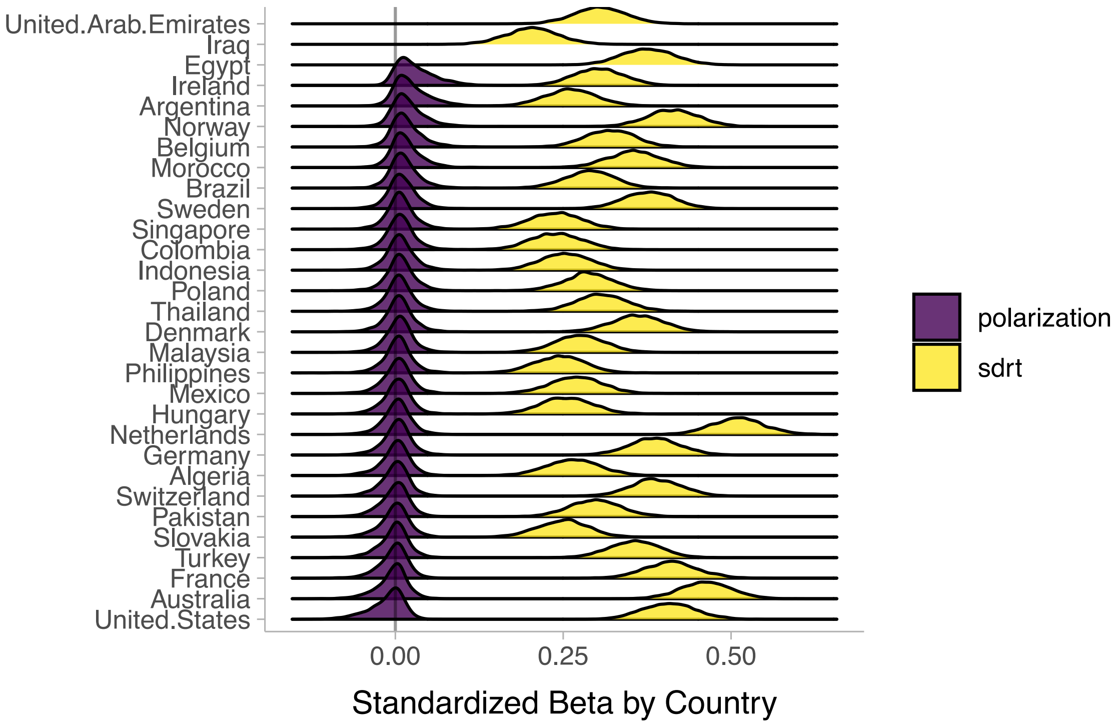

Note: Estimates are based on Bayesian multilevel regressions with weakly informative priors. Measures of affective polarization are omitted from these analyses in the three countries: UAE, Iraq, and Egypt.

J The role of status-driven risk taking across different countries

We regressed status-driven risk taking on our 3 macro variables, while also adding varying intercepts by country. Figure 14 reports the coefficient estimates reported in the main text.

Supplementary Fig. 14: In more democratic countries there are consistently fewer status-driven respondents. Meanwhile, we find little differences in levels of status-driven risk taking across inequality and poverty.

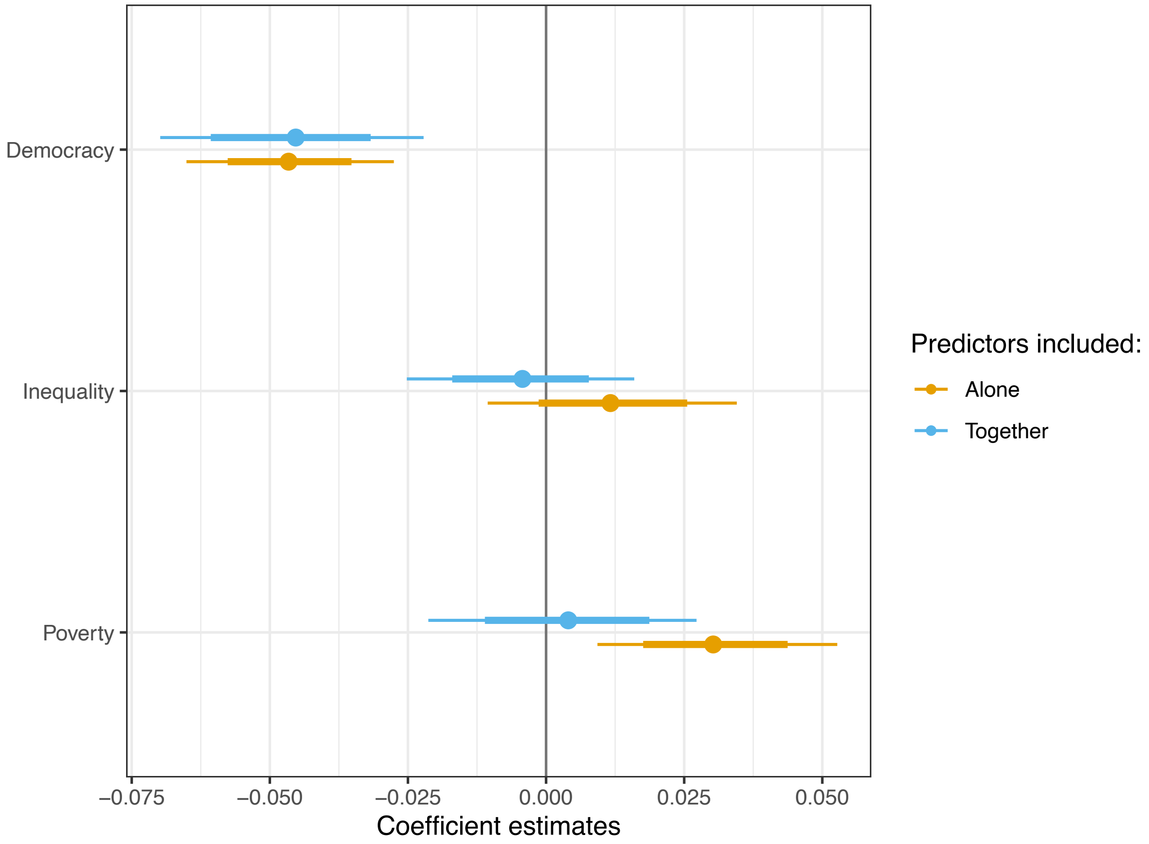

Note: Estimates are based on Bayesian multilevel regressions with weakly informative priors.

We rerun our main multilevel models testing H2 – regressing self-reported online political hostility (as a perpetrator) on status-driven risk taking – while adding a cross-level interaction with democracy and inequality, respectively. These models test if the association between hostility and status-drive depend on the level of democracy or inequality in the respondent's country. As reported in the main text, we find a larger interaction between status-drive and liberal democracy than between status-drive and inequality (see Figure 15).

Supplementary Fig. 15: In more democratic and less equal countries the relationship between status-driven risk taking and online political hostility is stronger.

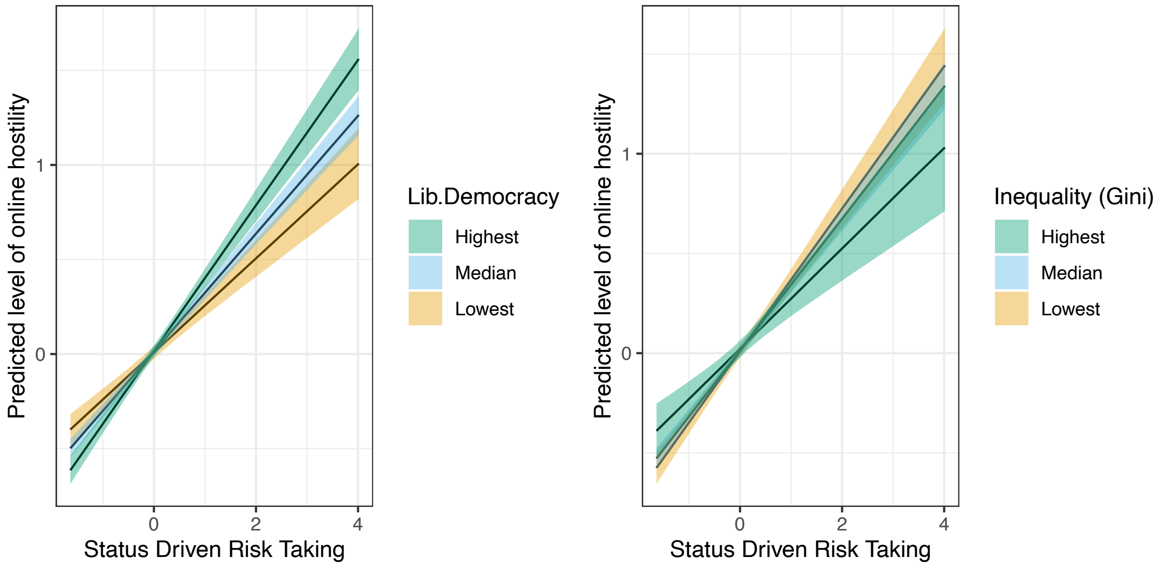

Note: Estimates are based on Bayesian multilevel regressions with weakly informative priors.

K Young male syndrome in equal and unequal societies

First, we are interested in the role of age. We first assess the extent to which cross-national differences in hostility reflect demographic composition, we estimated a Bayesian multilevel model with individual hostility as the outcome, age categories and liberal democracy as predictors, and country-specific random intercepts and age slopes. Using posterior predictions from this model, we computed for each country the expected mean level of hostility under (i) its observed age distribution and (ii) a common reference age distribution pooled across countries. The difference between these two quantities captures the compositional contribution of age structure to country-level hostility. We summarize this difference using posterior medians and 89% credible intervals. For ease of interpretation, we express these differences standardized by the estimated between-country standard deviation of the random intercepts.

We illustrate the well known fact that there are more older people in more democratic societies in Figure 16. Figure 17 demonstrates how much a given country's age distribution shifts predicted hostility. Figure 18 depicts the relationship between the compositional effect of age and VDem's liberal democracy scores. There is a strong negative correlation between the two (Pearson's r = -0.74).

Supplementary Fig. 16: In less democratic countries, there are more young respondents.

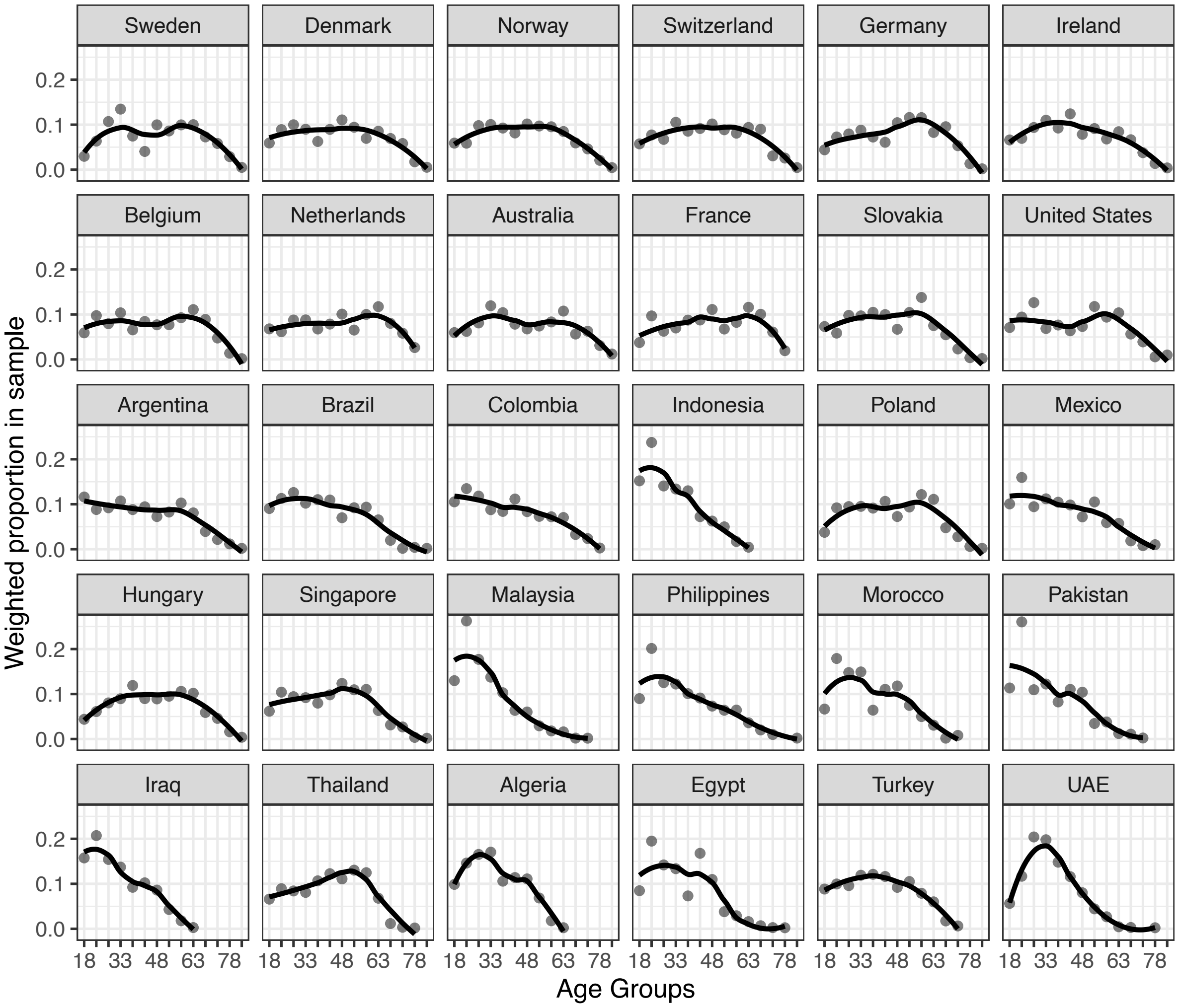

Note: Countries are ordered according to V-Dem's liberal democracy scores from most to least democratic in our sample. Points denote weighted proportion of respondents in a given age group. The black lines are loess curves highlighting the distribution of age within the country.

Supplementary Fig. 17: How much countries' age distributions shift predicted hostility

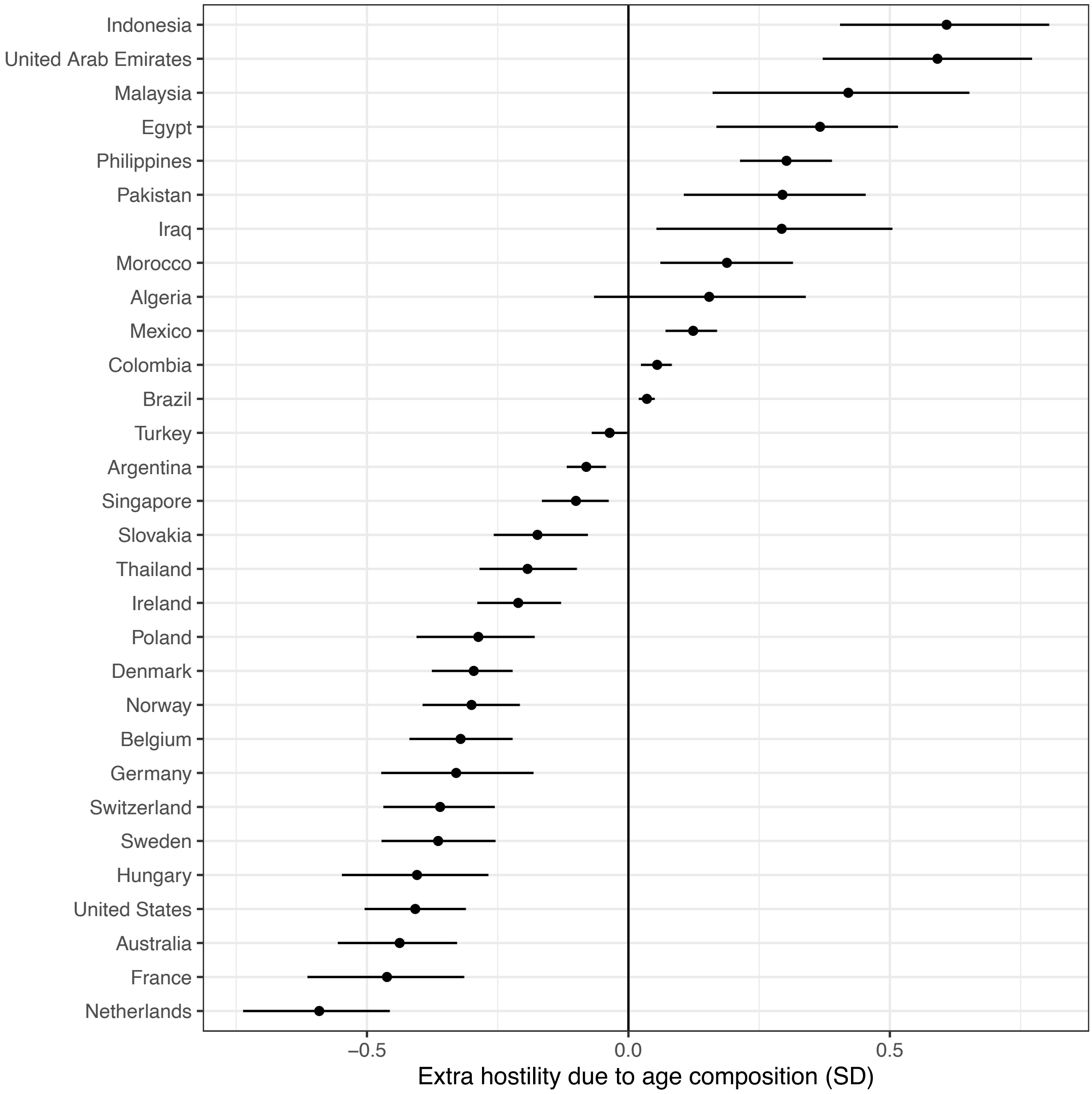

Note: Errorbars are 89% credible intervals.

Supplementary Fig. 18: Less democratic countries on average experience more hostility due to the age distribution in their populations

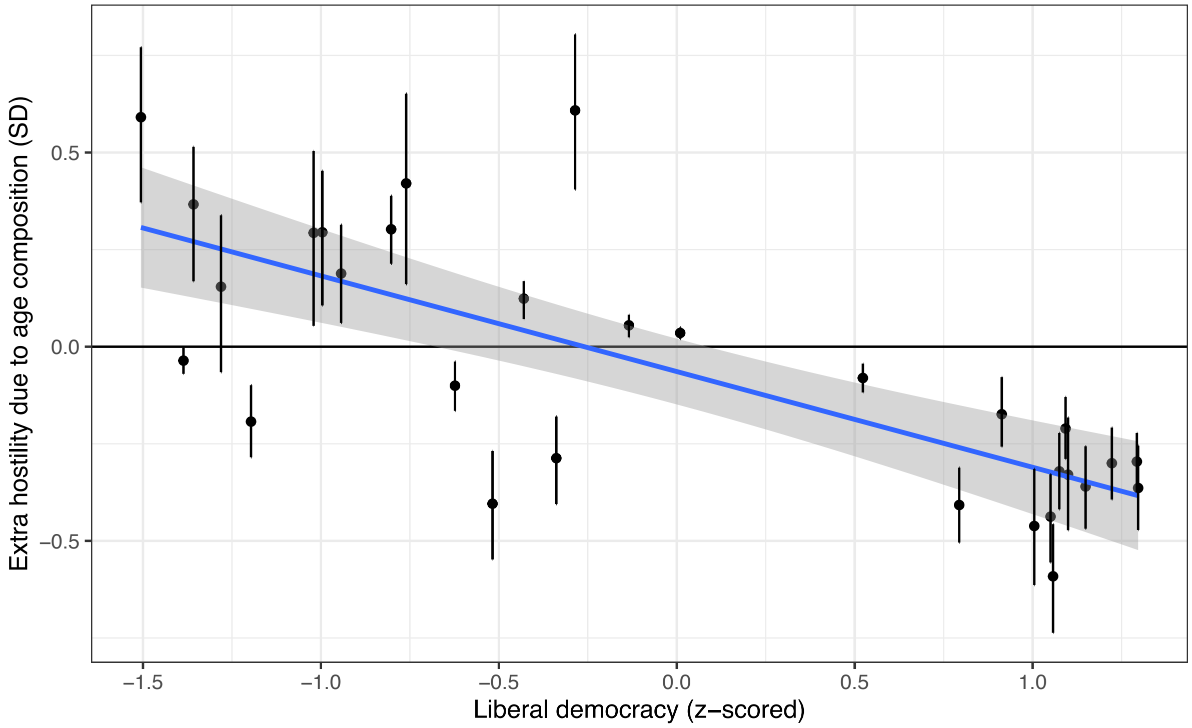

Note: Errorbars are 89% credible intervals.

To test the young male syndrome – that most hostility is committed by young men – we divide our sample by gender, and by age (using demographics groups defined by the survey data provider, while acknowledging that alternative splits could also be justified: 18–34, 35–54, and 55+). Next, we model online political hostility as a function of these six demographic groups, liberal democracy, and an interaction between the two. We find strong evidence that young men are most hostile than other demographic groups. We quantify this for average level of liberal democracy in Table 12.

Supplementary Table 12: Quantifying how much more hostile young men are compared to other age groups at average levels of democracy

Group compared to	Estimate	CI.Lower	CI.Upper	Post.Prob
Women 18-34	0.08	0.09	0.06	1
Men 35-54	0.06	0.07	0.05	1
Women 35-54	0.10	0.11	0.09	1
Men 55+	0.09	0.10	0.08	1
Women 55+	0.11	0.12	0.10	1

Finally, we turn to the interaction between demographic groups and levels of democracy. We find that women across all age groups, as well as men above 55 are less hostile if they live in more democratic countries ($\beta_{women18-34}$= -0.01, 89% CI [-0.02; 0]; $\beta_{women35-54}$= -0.01, 89% CI [-0.02; 0]; $\beta_{women55+}$= -0.02, 89% CI [-0.02; -0.01]; β_{men55+}= -0.02, 89% CI [-0.03; -0.01]), but not men between 18–34 ($\beta_{men18-34}$= 0.01, 89% CI [-0.01; 0.02]) or 35–54 ($\beta_{men35-54}$= 0.0, 89% CI [-0.01; 0.01]). Applying contrasts, our data and model show meaningful differences in the democracy-hostility relationship between the men aged 18–34 and aged 35–54 on the one hand, and men 55+, women 18–34, and women 55+ on the other, but not women aged 35–54 with respect to their hostility across levels of liberal democracy.

L How do people across the world perceive the impact of social media on politics?

The perceived impact of social media on politics

Our main investigation has focused on *political hostility* as a key aspect of citizens' experiences with social media. We showed that online hostility is worse in less democratic and unequal countries and that it strongly correlates with offline hostility and status-driven risk-taking. Given the widespread concerns about the impact of social media, including in highly democratic countries[8], we also wanted to explore how people across societies perceive the broader role of social media: Do they see it primarily as a negative force that exacerbates societal turmoil, or do they also view it as offering political opportunities that might outweigh its downsides?

To explore this, we measure the endorsement of three dominant narratives in public and scholarly discourse about the political effects of social media, as outlined by Tucker and colleagues.[9] These narratives offer a structured framework for understanding how different societies conceptualize the role of social media in political life. The *turmoil* narrative sees social media as amplifying extremism and misinformation, distorting public opinion, and undermining democracy. The *liberation* narrative emphasizes their role in democratizing discourse and fostering political engagement. Finally, the *oppression* narrative highlights how illiberal states exploit them for manipulation and repression.

We designed a seven-item survey measure to capture the three dominant narratives of social media's impact on politics: liberation (three items, e.g. "Social media helps people to take political action more effectively"), turmoil (two items, e.g. "Social media makes it too easy for people with extreme views to dominate public debates."), and oppression (two items, e.g. "Social media helps governments intimidate those who are critical of the government."). Note that one liberation item, one turmoil item, and both oppression items were deemed overly sensitive to be asked in Egypt, Iraq, and the UAE. We use multiple imputation to calculate the liberation and turmoil indices where at least one item was available but exclude these countries from the analyses of oppression (see Online Appendix O for details). We measure the level of

[8] Jonathan Haidt. After babel: How social media dissolved the mortar of society and made America stupid. The Atlantic, 329(4):54–66, 2022

[9] Joshua A Tucker, Yannis Theocharis, Margaret E Roberts, and Pablo Barber´a. From liberation to turmoil: Social media and democracy. Journal of Democracy, 28:46, 2017.

Supplementary Fig. 19: The perceived impact of social media on politics.

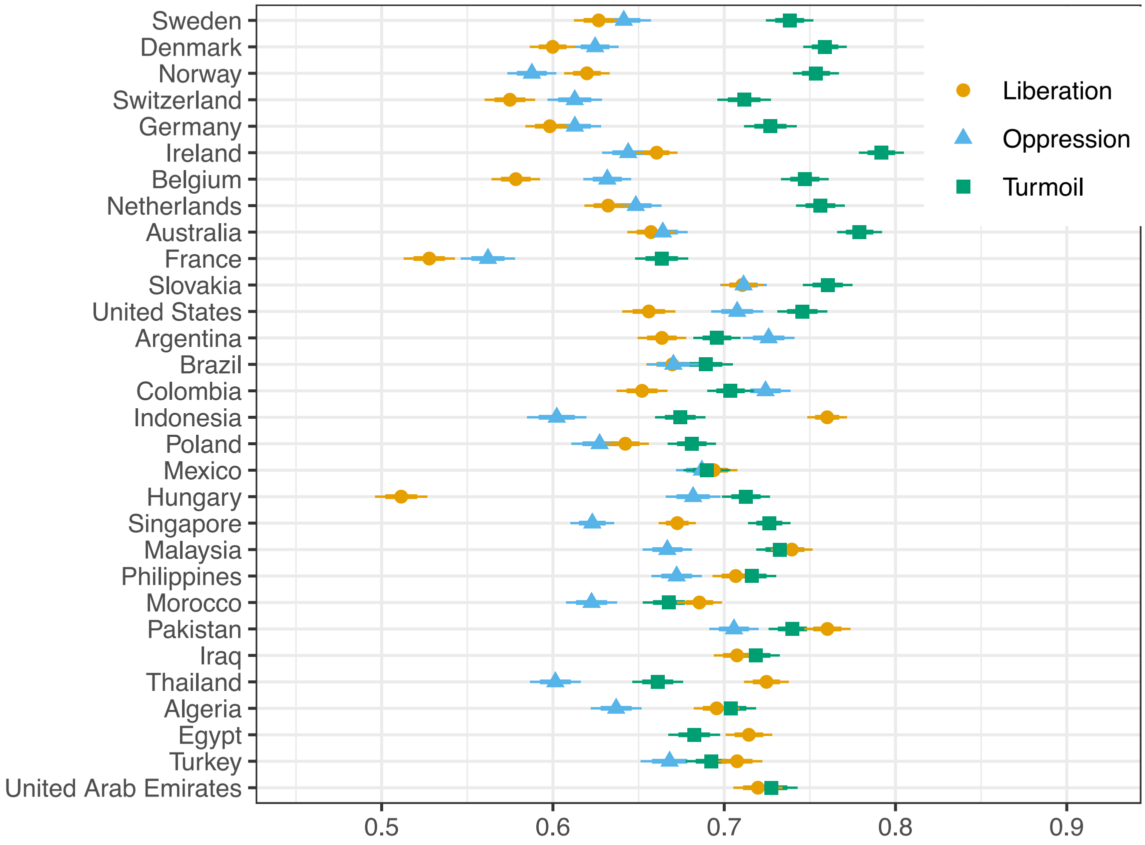

Note: Countries are ordered according their V-Dem liberal democracy score. Points denote simple unweighted country means on a 0-1 scale. Errorbars denote 67% and 89% confidence intervals.

agreement with these items using a seven-point Likert scale ranging from "strongly disagree" to "strongly agree".

Importantly, the results in Figure 19 demonstrate that people across a diverse global sample do not see the three narratives as mutually exclusive. All three narratives received more agreement than disagreement on average in every country. Global publics have not abandoned the emancipation-related optimism of the early days of online technology but have a nuanced view that social media brings about multiple potentials and pitfalls. However, one narrative, turmoil, received higher average support in the global sample than liberation ($\Delta M = 6\%$ points, 89% CI = [0.05, 0.06]) and oppression ($\Delta M = 7\%$ points, 89% CI = [0.07, 0.07]).

The perceived impact of social media on politics across (un)democratic and economically (un)equal countries

To examine how differences in democracy and economic inequalities relate to these perceptions, we correlate cross-national endorsements of the three narratives about social media with our measures of democracy and economic inequality. To do so, we regress the endorsements of the three narratives about social media (liberation, oppression, and turmoil, one-by-one) on z-scored measures of liberal democracy, economic inequality, and poverty, while also adding varying intercepts by country. Because we used multiple imputation to estimate the values of banned items on the liberation and turmoil scales (CF OA Section O), our regressions are fitted to each imputed dataframe, and the posteriors are averaged to account for the added uncertainty. Figure 20 displays the coefficient estimates.

Supplementary Fig. 20: Predicted difference in impressions of social media causing liberation, oppression, and turmoil associated with 1 standard deviation difference in liberal democracy, inequality and poverty.

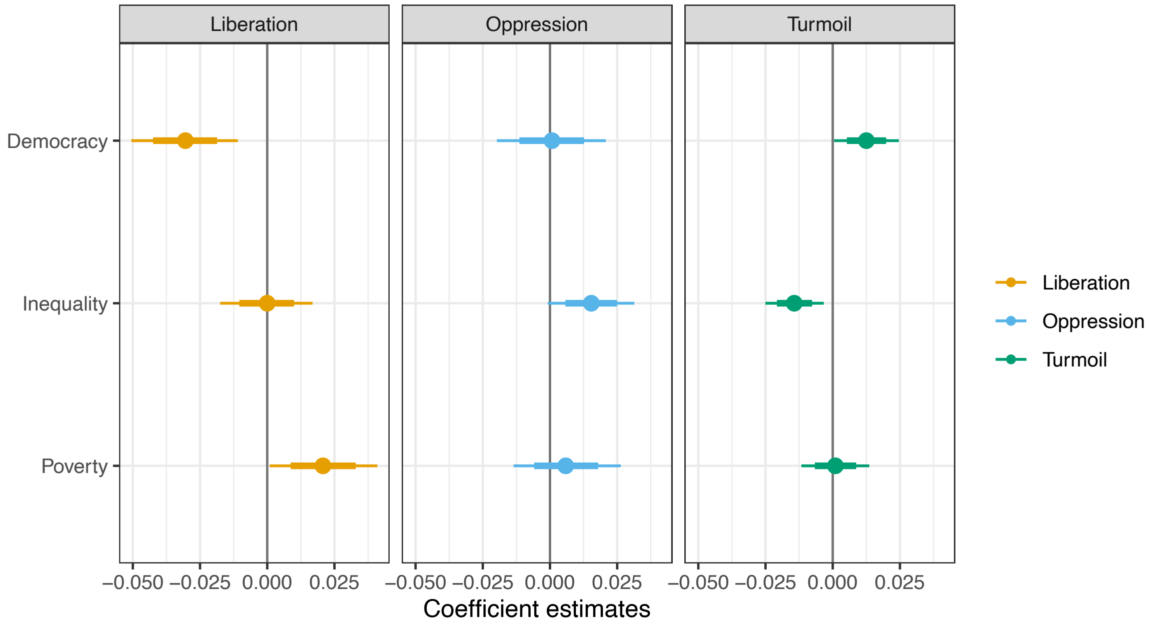

Note: Points denote coefficient estimates. Errorbars denote 67% and 89% credible intervals.

At first sight, the findings seem to be in conflict with our main findings on the cross-national variation in online hostility. People in more equal and more democratic countries associate social media with turmoil slightly more despite reporting that they fall victim to

hostility less often ($\beta_{democracy}$ = 0.01, 89%CI [0.00; 0.02], $\beta_{inequality}$ = -0.01, 89% CI [-0.03; -0.00]). Our data also show that people who live in less democratic countries consistently value social media more as a "liberation technology", i.e., as a platform for debate, discussion, and self-organization, even though these individuals are on the receiving end of substantially more online hostility ($\beta_{democracy}$ = -0.03, 89%CI [-0.05; -0.01]). Finally, we find little evidence that these macro-variables correlate with perceptions of social media as oppressive (the largest difference is between equal and unequal countries, yet we cannot rule out that this difference is due to chance $\beta_{inequality}$ = -0.01, 89% CI [-0.03; 0.00]). Overall, these results lead to the pattern in Figure 19, which orders countries on the y-axis by their level of liberal democracy. We see that in democratic countries, social media are viewed overwhelmingly as a source of turmoil. In less democratic countries, the assessment is more mixed, and the liberation narrative often outweighs perceptions of turmoil and oppression.

These results are robust across different types and levels of democracy, as well as across different conceptualizations of equality across socio-economic status (SES). We use Spearman's rank correlations (ρ) to measure the strength and direction of the relationships between perceptions of social media and different types of democracy as well as degree of equality across SES.

Figure 21 shows that social media is on average perceived as less liberating in more democratic countries compared to less democratic countries, regardless of the conceptualization of democracy (*Electoral Democracy Index:* ρ = -0.73, $p < 0.01$; *Participatory Democracy Index:* ρ = -0.73, $p < 0.01$; *Deliberative Democracy Index*: ρ = -0.69, $p < 0.01$; *Egalitarian Democracy Index:* ρ = -0.78, $p < 0.01$). In contrast, people in more democratic countries are on average more likely to perceive social media as turmoil than people in less democratic countries, regardless of the conceptualization of democracy (*Electoral Democracy Index:* ρ = 0.41, $p < 0.05$; *Participatory Democracy Index:* ρ = 0.37, $p < 0.05$; *Deliberative Democracy Index*: ρ = 0.38, $p < 0.05$; *Egalitarian Democracy Index:* ρ = 0.38, $p < 0.05$). The results for seeing social media as oppression are less clear. While the data suggests a general trend for people in more democratic countries to on average perceive social media as less oppressive than people in less democratic countries, regardless of the specification of democracy, the associations are not statistically significant (*Electoral Democracy Index:* ρ = -0.18, $p = 0.37$; *Participatory Democracy*

Index: ρ = -0.18, p = 0.36; *Deliberative Democracy Index*: ρ = -0.3, p = 0.12; *Egalitarian Democracy Index:* ρ = -0.29, p = 0.14).

Figure 22 shows that — parallel to the findings for different levels and types of democracy — in countries with more equality across socioeconomic status, people are on average less likely to see social media as liberation (*Equal Power Distribution by SES:* ρ = -0.63, $p < 0.01$; *Equal Access to Public Services by SES:* ρ = -0.66, $p < 0.01$; *Equal Access to State Jobs by SES:* ρ = -0.67, $p < 0.01$; *Equal Access to State Business Opportunities by SES:* ρ = -0.71, $p < 0.01$), and slightly more likely to see it as turmoil (*Equal Power Distribution by SES:* ρ = 0.26, p = 0.17; *Equal Access to Public Services by SES:* ρ = 0.32, p = 0.08; *Equal Access to State Jobs by SES:* ρ = 0.38, $p < 0.05$; *Equal Access to State Business Opportunities by SES:* ρ = 0.36, p = 0.05) than people in countries with less equality in terms of SES, however, the latter relationships vary on statistical significance. Other than for democracy indicators, however, the results for the perception of social media as oppression are a bit stronger when it comes to equality. People in countries with more equality across SES are on average less likely to see social media as oppression than people in countries with less equality across SES (*Equal Power Distribution by SES:* ρ = -0.31, p = 0.12; *Equal Access to Public Services by SES:* ρ = -0.56, $p < 0.01$; *Equal Access to State Jobs by SES:* ρ = -0.38, $p < 0.05$; *Equal Access to State Business Opportunities by SES:* ρ = -0.44, $p < 0.05$).

Supplementary Fig. 21: The Perceived Impact of Social Media on Politics Across Levels and Types of Democracy.

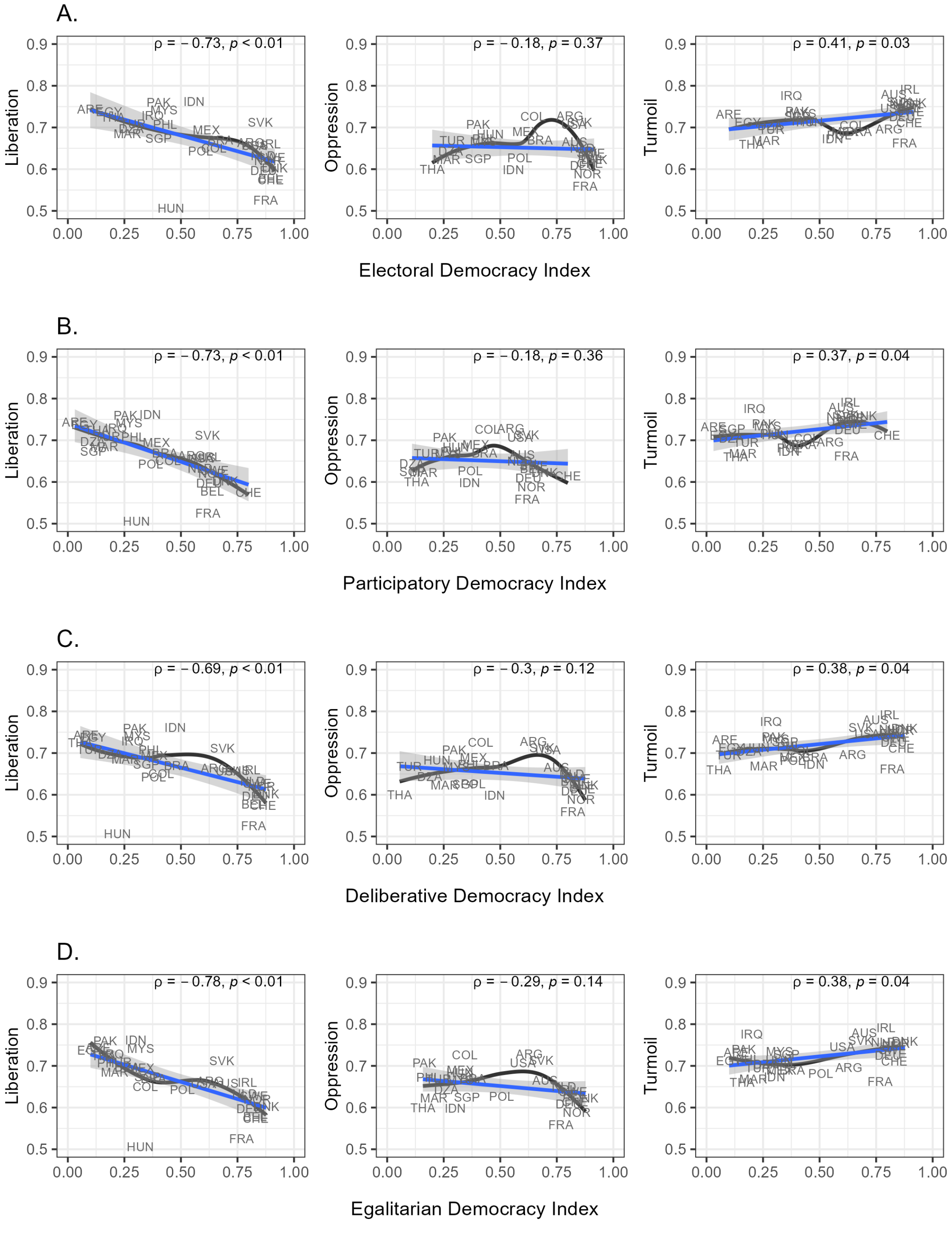

Note: Correlations of perceptions of the impact of social media on politics across different types and levels of democracy: Electoral Democracy (Panel A), Participatory Democracy (Panel B), Deliberative Democracy (Panel C), and Egalitarian Democracy (Panel D). Scatterplots show Spearman's rank correlations (ρ) with p-values in the upper right corner, linear trend lines in blue with 95% confidence intervals in grey, and Lowess curves in black.

Supplementary Fig. 22: Correlations between the perceived impact of social media on politics and equality by socio-economic status.

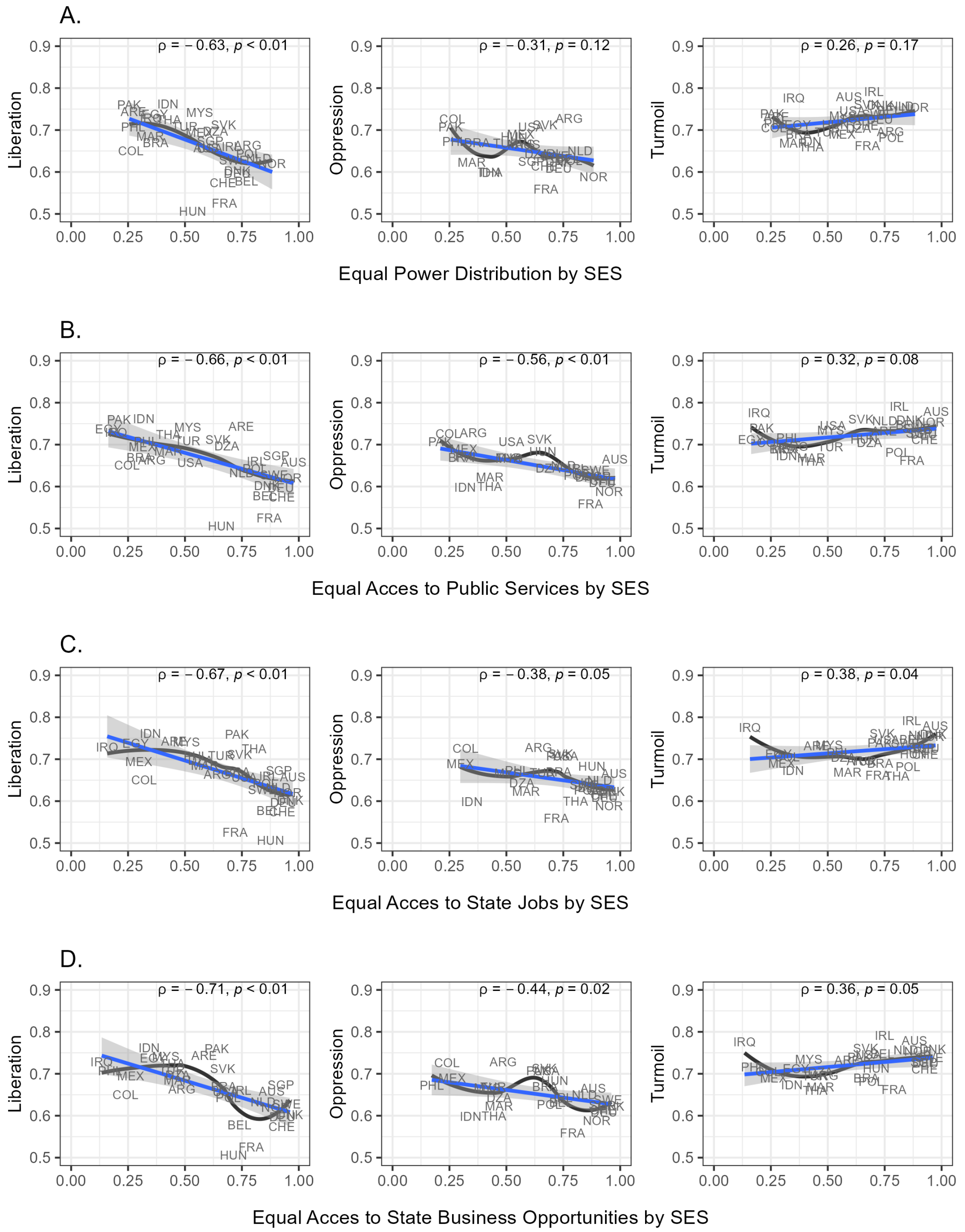

Note: Correlations of perceptions of the impact of social media on politics across different levels of equality by socio-economic status: Equal Power Distribution (Panel A), Equal Access to Public Services (Panel B), Equal Access to State Jobs (Panel C), and Equal Access to State Business Opportunities (Panel D). Scatterplots show Spearman's rank correlations (ρ) with p-values in the upper right corner, linear trend lines in blue with 95% confidence intervals in grey, and Lowess curves in black.

M Excluding respondents who never participate in political discussions

Supplementary Fig. 23: Excluding from the analyses those respondents who never participate in political discussions does not change our main conclusion regarding H2.

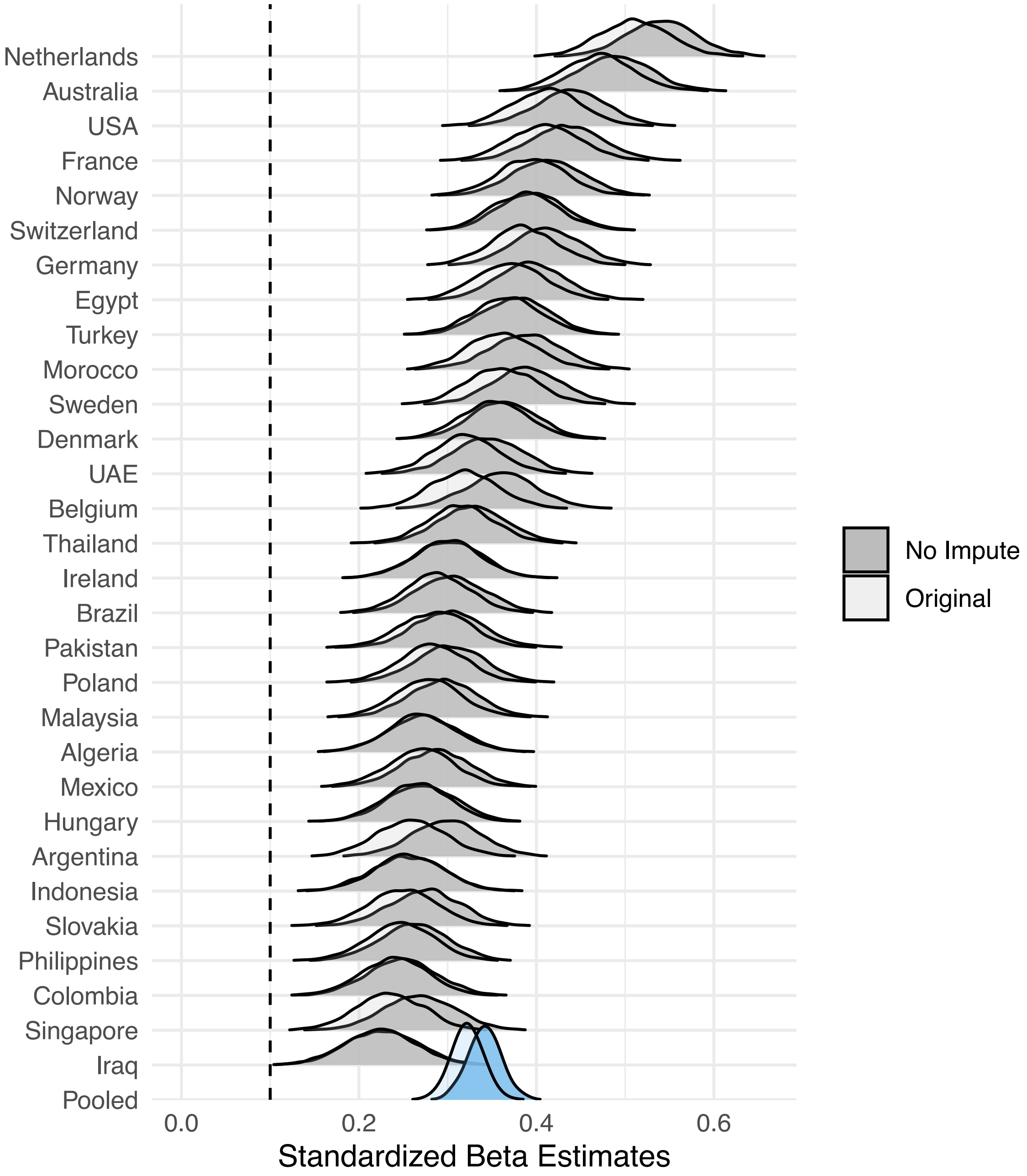

Note: Estimates are based on Bayesian multilevel regressions with weakly informative priors. The opaque estimates in the background display the original estimates. The darked estimates in the foreground are the robustness test without the imputation.

N Assessing measurement invariance

To ensure valid cross-national comparisons of political hostility and related constructs, we conducted multi-group measurement invariance tests across 30 countries for Online Hostility, Offline Hostility, Victimhood, and Status-Driven Risk Taking. By verifying configural, metric, and scalar invariance, we aimed to confirm that any observed differences across nations reflect genuine variations rather than measurement artifacts.

We report our results in Table 13. For Online and Offline Hostility, strong evidence emerged for configural, metric, and scalar invariance based on high CFI and TLI values (>0.94). Although the RMSEA occasionally exceeded 0.10 (e.g., 0.14 for the Online Hostility configural model), we retained the models because (a) CFI and TLI remained robust, and (b) changes in these indices (ΔCFI >-0.02, Δ MSEA <0.03) satisfied recommended cutoffs. Victimhood showed configural and metric invariance, but the full scalar model was rejected (ΔCFI = -0.03); partial scalar invariance was achieved by freeing the intercept of the third item ("I saw a comment that I found personally hurtful"), indicating that cultural differences in perceiving hurtful comments might affect baseline response levels. Status-Driven Risk Taking similarly satisfied configural and metric invariance (CFI and TLI $\geq$ 0.95), though the scalar model's RMSEA (0.11) exceeded the 0.10 criterion, warranting caution. Overall, these results support at least partial scalar invariance across all four constructs, permitting meaningful cross-national comparisons while acknowledging minor intercept adjustments for Victimhood.

Supplementary Table 13: Assessing measurement invariance

DV	Model	Chisq(df)	CFI	RMSEA	TLI	Comp.	ΔCFI	ΔRMSEA	Decision
Online Hostility	On 1: Configural	1531 (150)	0.97	0.14	0.94				Accept
	On 2: Metric	2442 (266)	0.95	0.13	0.95	On 1	-0.02	-0.01	Accept
	On 3: Scalar	3165 (382)	0.94	0.12	0.95	On 2	-0.01	-0.01	Accept
Offline Hostility	Off 1: Configural	1717 (150)	0.96	0.15	0.93				Accept
	Off 2: Metric	2364 (266)	0.95	0.13	0.95	Off 1	-0.01	-0.02	Accept
	Off 3: Scalar	3136 (382)	0.94	0.12	0.95	Off 2	-0.01	-0.01	Accept
Victimhood	Victim 1: Configural	389 (150)	0.99	0.06	0.99				Accept
	Victim 2: Metric	788 (266)	0.99	0.07	0.98	Victim 1	-0.01	0.01	Accept
	Victim 3: Scalar	1926 (382)	0.96	0.09	0.97	Victim 2	-0.03	0.03	Reject
	Victim 4: Partial Scalar	1339 (353)	0.97	0.08	0.98	Victim 2	-0.01	0.01	Accept
Status-driven risk taking	SDRT 1: Configural	240 (60)	0.99	0.08	0.98				Accept
	SDRT 2: Metric	424 (147)	0.99	0.06	0.99	SDRT 1	0	-0.02	Accept
	SDRT 3: Scalar	1687 (234)	0.94	0.11	0.95	SDRT 2	-0.05	0.05	Reject

O Diverging measurement of status-driven risk taking and the impact of social media on politics

In three countries, namely, Egypt, Iraq, and the United Arab Emirates, we were asked to change the original status-driven risk taking (SDRT) item from "I would enjoy being a famous and powerful person, even if it **meant a high risk of assassination**" to "I would enjoy being a famous and powerful person, even if it **put me at risk**". Figure 24 demonstrates that neither excluding these 3 countries, nor using only the 3 other SDRT items, changes our main conclusion about the relationship between online hostility and status drive (H2).

In the same 3 countries, we had to drop multiple items related to the perceived impact of social media on politics. Specifically, we were not allowed to ask any of our questions on perceived oppression, and had to drop one item each from the subscales on perceptions of liberation and turmoil too. Figure 25 demonstrates that compared to a naive estimate of liberation and turmoil impression where we simply ignore missing-data issue, our estimates relying on multiple imputations are slightly lower when it comes to the three affected countries.

Supplementary Fig. 24: Robustness tests the average relationship between SDRT and online hostility. The estimates are similar across all three model specifications.

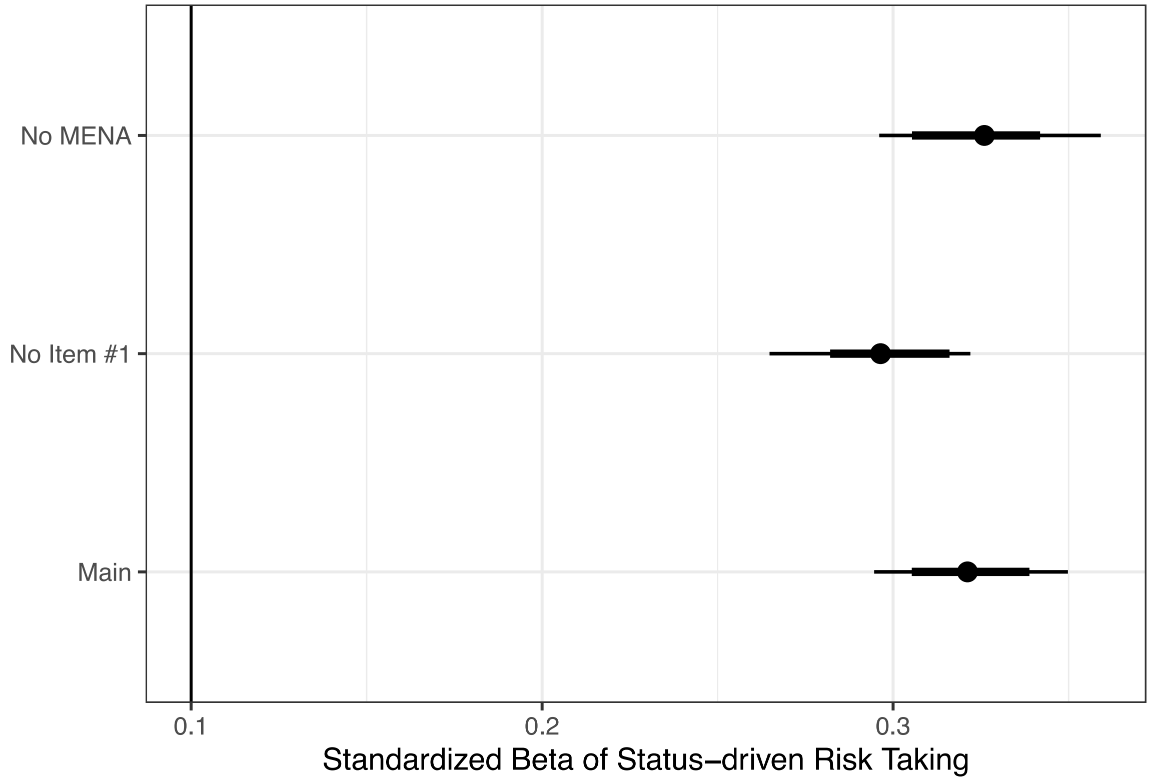

Note: Estimates are based on Bayesian multilevel regressions with weakly informative priors. Errorbars denote 67% and 89% credible intervals.

Supplementary Fig. 25: Imputed versus raw estimates of impressions of social media's political effects across all countries. Estimates are identical except for the three countries affected by omitted variables (Iraq, Egypt, and UAE).

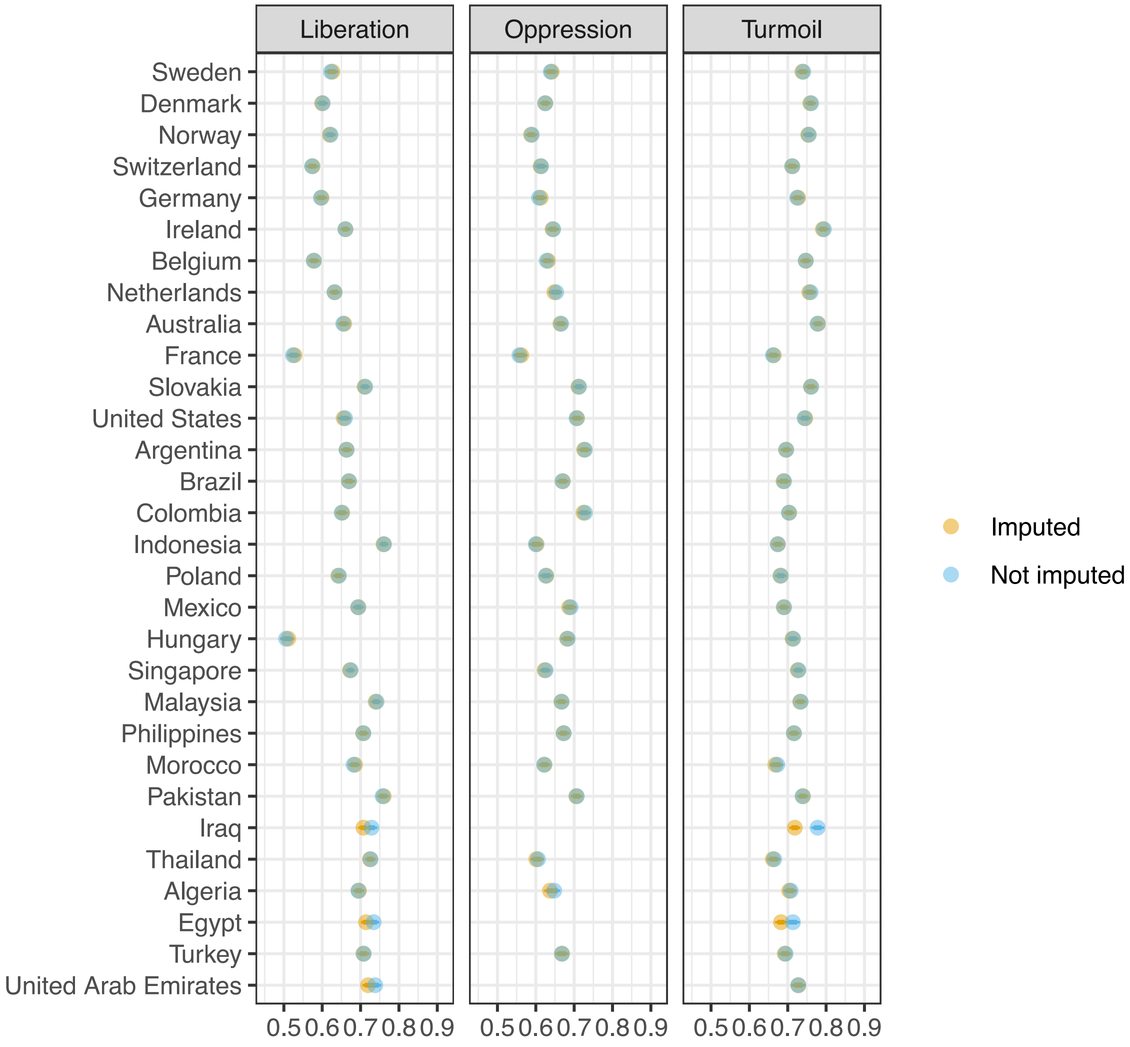

P Weakly informative priors in our models

Our Bayesian multilevel regressions testing Hypotheses 1-2-3 used the weakly informative priors described in Table 14. Note that all variables were transformed to z-scores before model fitting, so these priors operate on a standardized scale. All these priors seek to constrain coefficients to plausible ranges on standardized scale. For example, the fixed effects priors say that the standardized beta estimates (in both country- and individual-level associations) should not be larger than +2 or smaller than -2. Given that standardized beta estimates are generally within the +1/-1 range, this is a very weak assumption indeed. The priors on the intercept may seem more strict, yet because all coefficients are z-scored, intercepts are always very close to 0 (countries with average democracy in the sample have average hostility, per definition; similarly, people with average levels of status-driven risk taking have display average levels of hostility). Finally, the LKJ(4) prior mildly favors smaller correlations among random effects but still permits a wide range of covariance structures.

Supplementary Table 14: Specified priors for the Bayesian multilevel model

**Parameter class**	**Prior**
Fixed effects (`b`)	Normal(0, 1)
Intercepts	Normal(0, 0.5)
Group-level correlations (`cor`)	LKJ(4)

Q Equivalence of political hostility online and offline

Within-respondent comparisons

Our main analyses show that there is a very high correlation between online and offline political hostility: the people who are more hostile online, are also more hostile offline. A different way to approach the same problem is to ask, are there differences in the observed levels of hostility? As our theory indicates that these differences are likely small, we turn to equivalence tests now.

First, we use the pooled and unstandardized data to ask if we can reject the null hypothesis that within respondents, the average difference between self-reported online and offline hostility (as a perpetrator) is larger than -.1 standard deviation and smaller than .1 standard deviation. Put differently, using two one-sided t-tests, we ask if our data would be surprisingly small given assuming a meaningful effect size in the population. If we can reject the null, we can conclude that the effect is equivalent to 0.

As we demonstrate below, the average within respondent difference is statistically equivalent to 0, (ΔM = 0.002, Cohen's d = 0.02, $p < 0.001$, N = 14,585).

One may worry that us imputed all those 0s for people who never participate in discussions about politics may bias this comparison downwards but we can show that results are equivalent if we rely only on respondents who at least occasionally discuss politics (ΔM = 0.002, Cohen's d = 0.02, $p < 0.001$, N = 13,628).

Next, we repeat the same analysis but for each country separately. Figure 26 above reports country-level differences between online and offline hostility, benchmarked to the country-level standard deviation in online hostility. Larger values indicate more hostility online. We find two countries – Philippines, Iraq – where respondents report being slightly yet significantly more hostile online than offline. In 14 countries the difference in hostility is equivalent to 0. Conversely, there are 5 countries, where people report slightly, yet significantly more hostility from offline than from online conversations – Sweden, Germany, Switzerland, Argentina, Morocco. The main conclusion from these analyses is that in all countries, people report very similar levels of online and offline hostility on average. Even the largest difference we observe (in Morocco) amounts to only to 2% of the 0-1 scale we use to measure political hostility.

Supplementary Fig. 26: Country-level differences between online and offline hostility within respondents

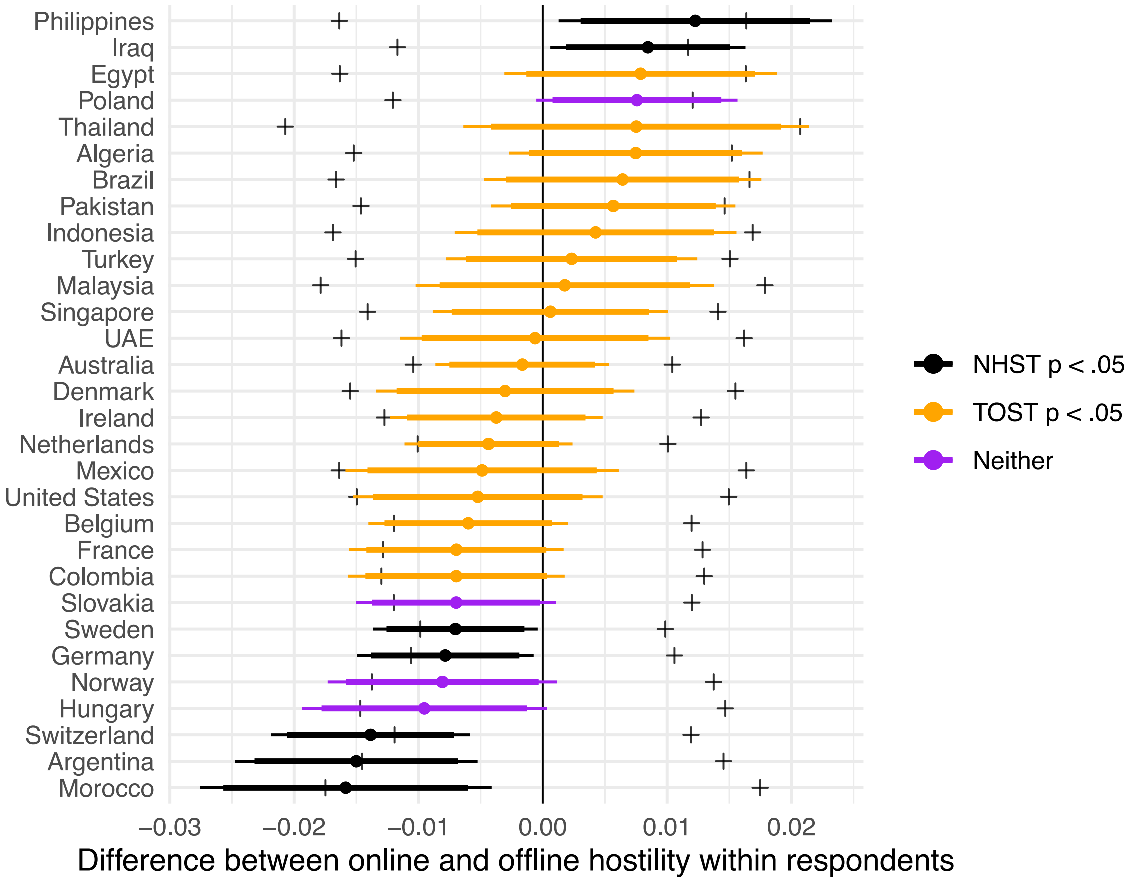

Note: The plus signs denote the equivalence bounds. Errorbars denote the 95% confidence intervals for the null-hypothesis test and the TOST equivalence test. Estimates are colored according to the conclusion of the equivalence test: significantly different from zero (black), equivalent to zero (yellow), or inconclusive (purple).

Between-group comparisons of online versus offline hostility

Next, we exploit the design feature in our study that the online and offline hostility (as perpetrator) batteries were randomized to be asked towards the beginning of our survey versus at the very end. Our main analyses use all available data and therefore perform within-respondent comparisons. Yet one may worry that having asked a similar question twice biases answers, as respondents may be motivated to make their answers more consistent (i.e. common source bias) or more nuanced (i.e. "opportunity to revise").[10] Here, we restrict our data to whichever battery was asked first, and perform between-participant comparisons. This could be reconceptualized as estimating the effect of environment (online vs offline) on self-reported political hostility. Because we expect to find no effect, we perform equivalence tests, with the preregistered smallest effect size of interest, Cohen's d = 0.1.

The pooled analysis shows that the difference between online and offline political hostility is equivalent to 0 (ΔM = 0.00, Cohen's d = 0.00, $p < 0.001$, N = 13732).

We find only a single country (Hungary) where people who report their online behavior first report marginally more hostility (ΔM = 0.03, $p = 0.03$) than those who report about their offline behavior first. In 24 countries the difference between these two groups is equivalent to 0 (ps < 0.05), whereas in 5 countries (Argentina, Switzerland, Slovakia, Poland, and Thailand) the difference is close to 0, but the estimates are too noisy to reject the null hypothesis of the smallest effect size of interest. Again, notwithstanding the level of statistical significance, our difference estimates are all substantively small, amounting to 3% points on our 0-1 scale.

[10] McDonald J, Hanmer MJ. Evaluating methods for examining the relative persuasiveness of policy arguments. Political Science Research and Methods. Published online 2023:1-8. doi:10.1017/psrm.2023.54

Supplementary Fig. 27: Country-level differences between online and offline hostility between respondents

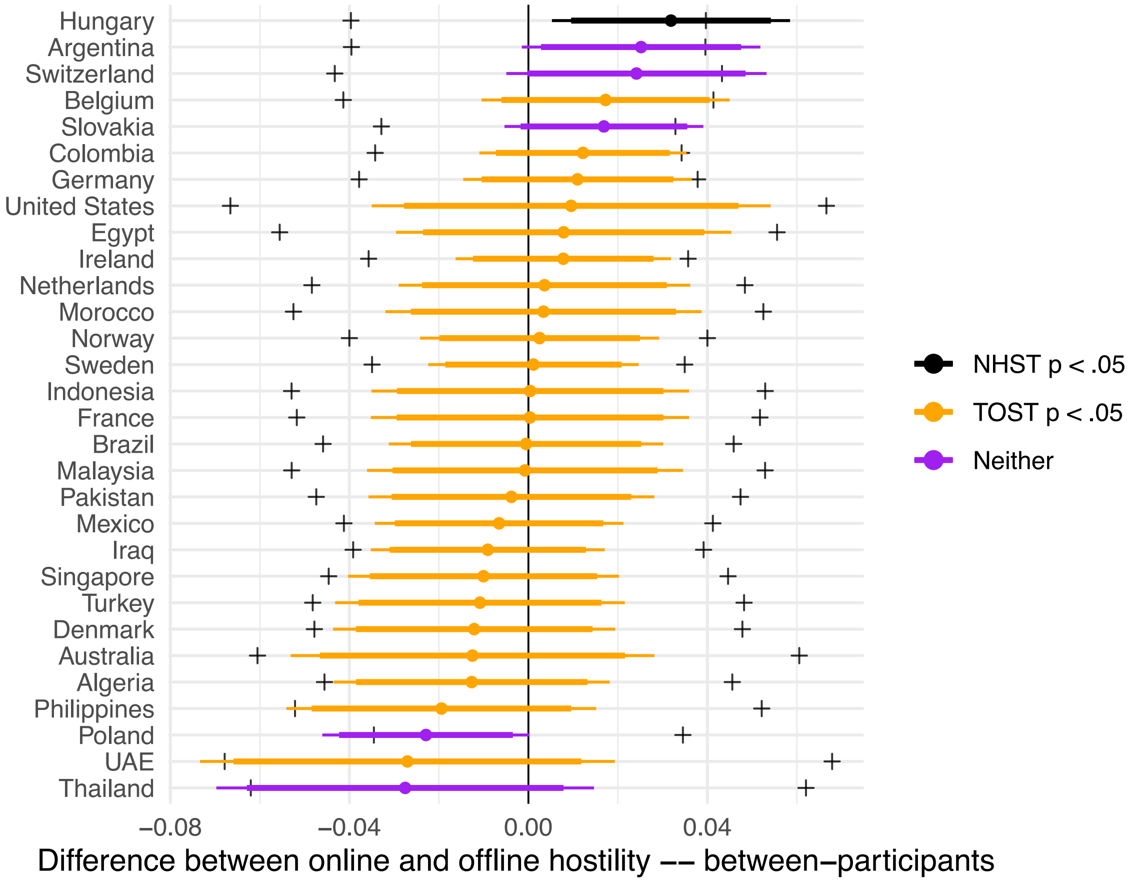

Note: The plus signs denote the equivalence bounds. Errorbars denote the 95% confidence intervals for the null-hypothesis test and the TOST equivalence test. Estimates are colored according to the conclusion of the equivalence test: significantly different from zero (black), equivalent to zero (yellow), or inconclusive (purple).

R Online versus offline hostility by items

Supplementary Fig. 28: Robustness tests the average relationship between SDRT and online hostility. The estimates are similar across all three model specifications.

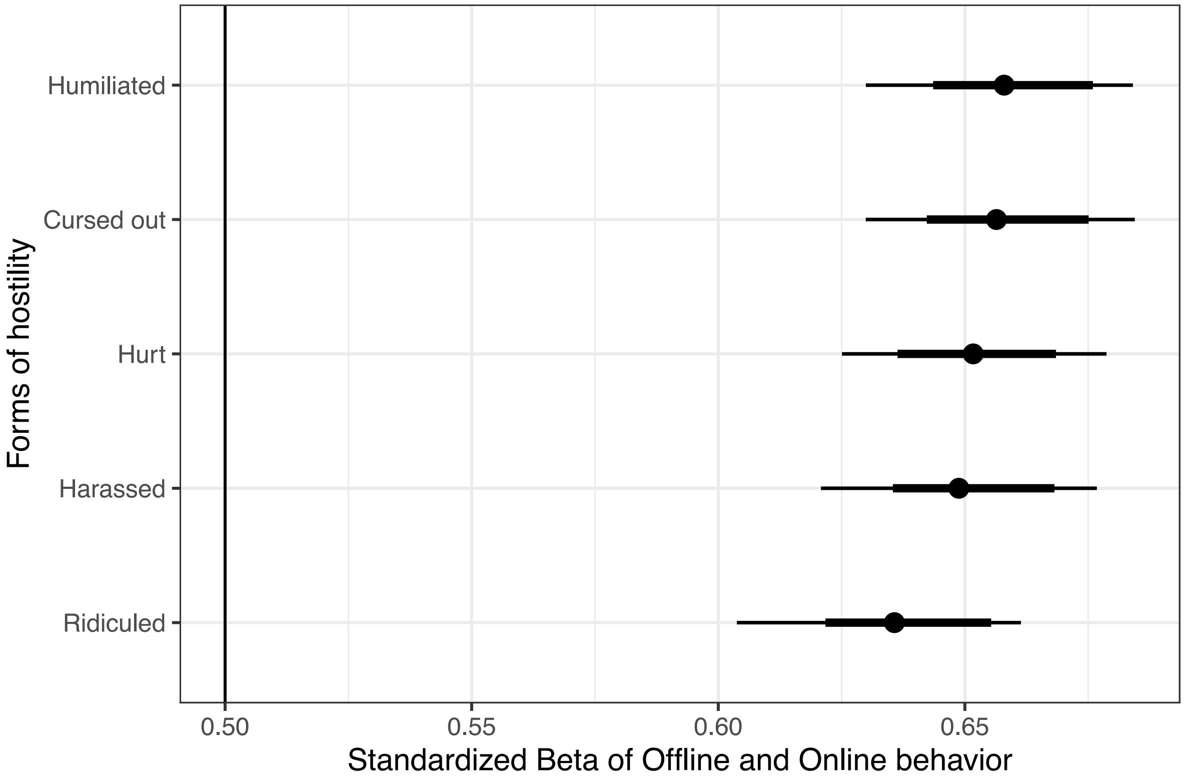

Note: Estimates are based on Bayesian multilevel regressions with weakly informative priors. Errorbars denote 67% and 89% credible intervals.

S Hostility and inequality across US States

Our main analysis demonstrated that less democratic and unequal countries experience greater political hostility on social media. While this cross-national approach provided strong evidence of a global pattern, it remained vulnerable to potential confounds, such as cultural, linguistic, or technological differences across countries. To further probe the robustness of our findings, we extend our analysis to the subnational level within the United States. By leveraging variation across U.S. states, we isolate the role of inequality while holding constant national-level institutional and cultural factors. This within-country approach allows us to examine whether the relationship between economic inequality and online hostility persists when democratic institutions, media landscapes, and regulatory environments are held constant. If similar patterns emerge across U.S. states as have benn observed across countries, this corroborates our argument that economic inequality—rather than national-level confounds—is a key driver of online hostility.

To examine the relationship between economic inequality and online hostility at the subnational level, we employ multilevel regression and poststratification (MRP), a widely used technique for generating reliable state-level estimates from nationally representative surveys. [11] Our survey was designed to approximate national demographic distributions but is not representative at the state level due to sample size constraints and uneven coverage. MRP addresses this limitation by modeling individual responses using a multilevel Bayesian regression, which borrows strength across observations, allowing states with smaller samples to benefit from patterns observed in states with larger samples. The poststratification step then ensures that estimates align with actual state-level demographic distributions.

Our data were collected via Lucid Theorem in July and early August 2023 as part of an omnibus study, yielding 4,246 respondents quota-sampled to approximate the U.S. population in terms of age, gender, education, race, and region. We measured self-reported online hostility using the same five-item scale as in our main cross-national analysis. To generate state-level estimates, we fit a Bayesian multilevel model predicting online hostility as a function of respondents' age, gender, education, race, state of residence, and Census region. Each of these

[11] See Gelman, A., & Little, R. J. A. (1997). Poststratification into many categories using hierarchical logistic regression. Survey Methodology, 23, 127–135. See also Park, D. K., Gelman, A., & Bafumi, J. (2004). Bayesian multilevel estimation with poststratification: State-level estimates from national polls. Political Analysis, 12(4), 375–385.

variables is entered as a random intercept into the model to prevent over-fitting. The model was estimated using the `brms` package, with weakly informative priors. As a next step, we estimate the online hostility for census cells, IE each unique combination of the demographic variables in our model (with the exception of region that is always matched to the corresponding state). In essence, we predict the hostility of 6120[12] types of US American citizens. We then take the weighted average of these census cell estimates using population weights from the 2022 American Community Survey, accessed via IPUMS USA.[13]. This combination of hierarchical modeling and demographic adjustment allows us to obtain precise and generalizable estimates of online hostility across U.S. states, providing a within-country test of the relationship between inequality and online hostility while holding national-level institutional and cultural factors constant.

To examine the relationship between economic inequality and online hostility across U.S. states, we regress our MRP-derived hostility estimates on state-level income inequality, measured using the Gini index. We obtain Gini estimates from the U.S. Census Bureau's data API via the `tidycensus` R package, ensuring consistency with official economic data sources.

Supplementary Table 15: Income inequality correlates with state-level online hostility

	Dependent variable: Online Hostility (z-scored)	
	between respondents	between states
	(1)	(2)
Gini (z-scored)	0.03	0.76
	(0.02, 0.04)	(0.56, 0.97)
Constant	−0.01	0.06
	(−0.01, 0.001)	(−0.12, 0.23)
Observations	51	51
R^2	0.43	0.43
Residual Std. Error (df = 49)	0.03	0.77
Note:	We report 89% confidence intervals in brackets.	

We report estimates from a simple ordinary least squares (OLS) regression, with two spec-

[12] 4 (age groups) × 2 (gender) × 5 (education) × 3 (race) × 51 (states) = 6120

[13] Ruggles, S., Flood, S., Sobek, M., Brockman, D., Cooper, G., Richards, S., & Schouwiler, M. (2023). IPUMS USA: Version 13.0. Minneapolis, MN: IPUMS. Available at https://usa.ipums.org.

Supplementary Fig. 29: People who live in US States with more income inequality are more hostile in online political discussions. State-level online hostility estimates are from MRP models. Both measures are standardized to variation across states.

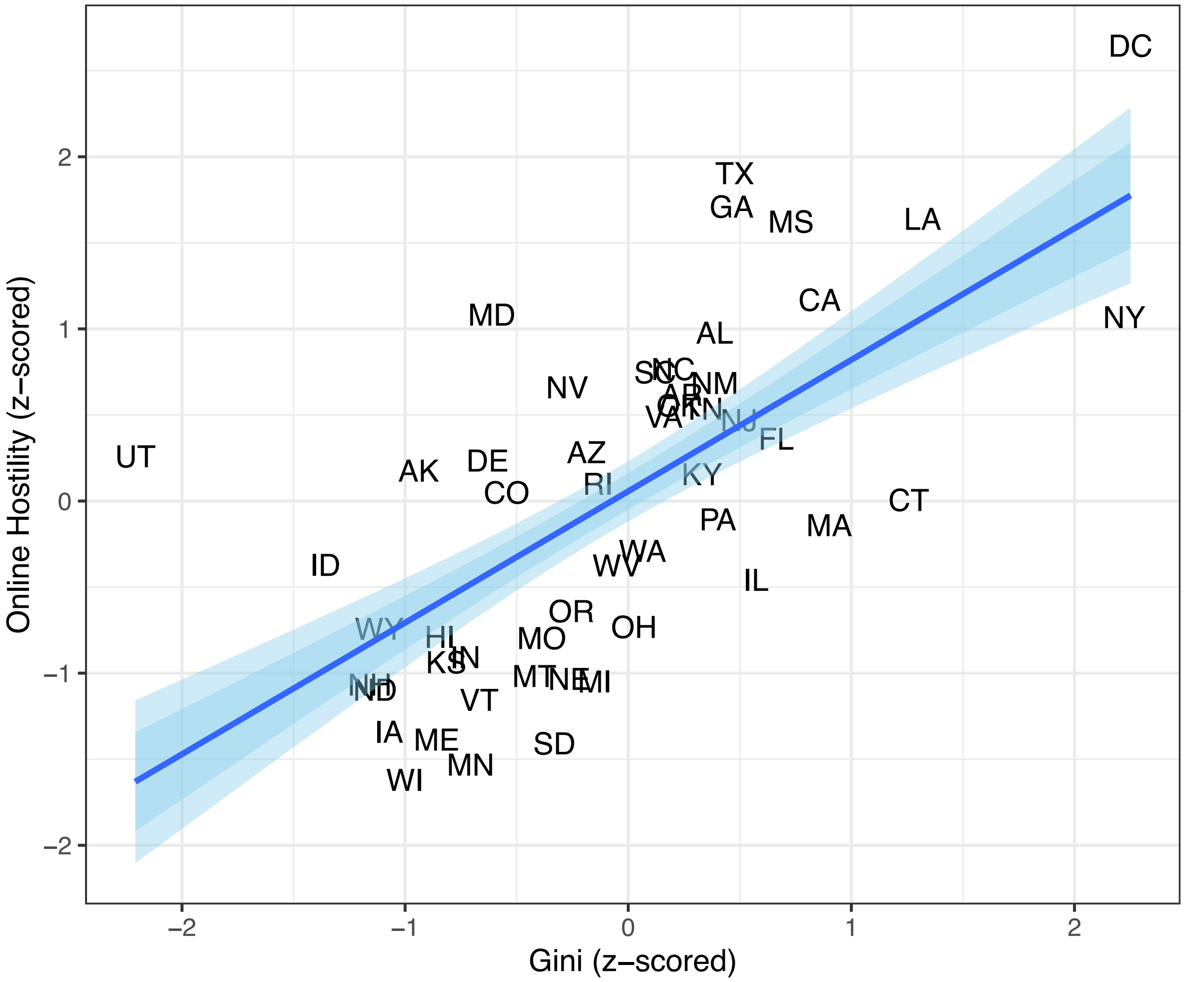

Note: Dark blue line is OLS regression line. Blue ribbons denote 67% and 89% confidence intervals.

ifications. The first model standardizes online hostility to the between-individual standard deviation. The second model standardizes hostility based on variation across states, allowing us to assess the relative differences in hostility across U.S. states. To facilitate interpretation, we also standardize the Gini index to reflect state-level variation.

As shown in Figure 29, states with higher income inequality exhibit greater levels of online hostility. The regression results in Table 15 confirm this pattern, with both specifications indicating a positive and statistically significant association between Gini and online hostility. It is notable that income inequality alone explains 43% of the variation in online hostility at the state level. These findings are consistent with our main cross-national analysis, further supporting the argument that economic inequality fosters a more hostile online environment.

T Analyzing Negativity on Twitter across Countries

Our primary analyses rely on self-reported measures of online political hostility, which offer a crucial advantage: they directly capture individuals' subjective experiences of hostility across diverse cultural contexts. Given that online hostility is, at its core, a social and psychological phenomenon, self-reports provide valuable insight into how people interpret and respond to political discussions. This approach also allows us to systematically examine predictors of hostility, including psychological dispositions and broader sociopolitical factors. While self-reports are not without limitations—such as potential recall biases or social desirability effects—we directly assess these concerns in Online Appendix C and find reassuring evidence that they do not undermine our conclusions.

To complement this approach, we also examine behavioral data from social media, which, in principle, provide an external benchmark for assessing cross-national patterns in online hostility. Unlike surveys, behavioral data offer large-scale, unobtrusive measures of public discourse. However, whether these data can serve as a meaningful cross-national benchmark remains an open question. Differences in platform usage, moderation policies, and linguistic contexts complicate direct comparisons, raising concerns about validity and representativeness.

To complement our survey-based analyses, we leverage the Twitter Sentiment Geographical Index (TSGI) dataset, a large-scale resource that provides sentiment estimates for over 1.5 billion tweets across 237 countries and territories[14]. This dataset offers several key advantages. First, it allows for a truly global comparison of sentiment expressed on social media, covering a broad range of political and economic contexts. Second, it relies on machine learning-based sentiment analysis, which provides an automated, large-scale assessment of online discourse that is free from self-report biases. While this dataset does not measure hostility per se, negative sentiment is a closely related construct that captures patterns of frustration, conflict, and antagonism in online discussions. Given the current limitations of multilingual hostility detection, sentiment analysis provides the best available alternative for cross-national comparisons of online negativity.

At the same time, using this dataset for comparative research comes with important challenges, particularly regarding cross-national validity and representativeness. As the original

[14] Luo, J., Lyu, H., Luo, H., Tang, Z., Kong, D., & Wen, X. (2023). Global sentiment tracking on Twitter during the COVID-19 pandemic. *Scientific Data, 10*, 744. https://doi.org/10.1038/s41597-023-02572-7

authors note:

> "This dataset is constructed based on the geotagged posts only, which accounts for approximately 1–2% of the total traffic at Twitter ... a relatively small proportion. Researchers face the inevitable trade-off between the comprehensive representativeness of tweets and the need for location information (Li et al.) ... A recent paper has found that geotagged posts are subjected to a bias of being happier compared to non-geotagged posts since people like to attach their tweets to a specific location to record the joyous and special events ... It is recommended that users pay more attention to the local sentiment variations, instead of the absolute sentiment value."

These concerns highlight the need for caution in interpreting the results. Geotagged tweets may not be fully representative of national online discourse, and cultural or platform-specific factors could introduce systematic biases. In the following sections, we explore the extent to which this dataset aligns with our survey-based measures of hostility and whether it can serve as a meaningful benchmark for cross-national comparisons.

The Twitter Sentiment Geographical Index dataset is structured as panel data, consisting of country-date observations that track daily sentiment levels across different nations. For our analyses, we focus on the most recent full year available in the dataset, 2022, to ensure consistency in temporal coverage across countries. Given that sentiment estimates are derived from geotagged tweets, the number of observations per country and day varies significantly. To mitigate extreme noise, we follow a standard filtering procedure and exclude country-date observations with fewer than 10 tweets. This ensures that our results are not driven by outliers or sparsely populated data points.

Temporal Validation: Sentiment Patterns on Weekends and Holidays

Before turning to cross-national comparisons, we conduct two temporal validation analyses as a face validity check. Prior research has consistently found that people report higher well-being on weekends and public holidays, a pattern reflected in various sentiment datasets[15]. Similarly, public holidays have been shown to correspond with temporary increases in positive

[15]Ryan, R. M., Bernstein, J. H., & Brown, K. W. (2010). Weekends, work, and well-being: Psychological need satisfactions and day of the week effects on mood, vitality, and physical symptoms. *Journal of Social and Clinical Psychology, 29*(1), 95–122. https://doi.org/10.1521/jscp.2010.29.1.95

sentiment on social media[16]. If our sentiment data capture meaningful fluctuations in public mood, we should observe systematically higher sentiment scores on weekends and holidays compared to regular weekdays. These patterns serve as a useful sanity check before proceeding to more complex cross-national analyses.

To assess the face validity of the sentiment estimates, we estimate two simple linear regression models predicting daily sentiment scores, using day of the week and national holidays as predictors. These models test whether the dataset captures well-established temporal fluctuations in public mood. All variables are standardized within each country to z-scores, meaning that the coefficients reflect changes in sentiment in standard deviation units relative to a country's usual variation over time.

1. **Day-of-the-Week Model** – We regress daily sentiment scores on a factor variable for the day of the week, with Monday as the reference category, and include country fixed effects to account for national differences in overall sentiment levels. This approach allows us to estimate the sentiment level for each day relative to Monday. Prior research suggests that sentiment should be higher on weekends compared to weekdays, reflecting work-life balance and leisure time.

2. **Holiday Effect Model** – We follow the same approach but include a binary variable for national public holidays, again with country fixed effects. Given that national holidays often bring celebrations, social gatherings, and time off from work, we expect sentiment to be higher on holidays than on regular weekdays.

The results, summarized in Table 16 and Table 17, align with expectations and reinforce the validity of the sentiment estimates.

- **Day-of-the-Week Effect:** People tweet more positively on weekends compared to weekdays, though the differences are not large. The most negative day (Wednesday) and the most positive day (Saturday) differ by just under a quarter of a standard deviation of within-country daily sentiment variability. This pattern is consistently with prior research showing that well-being tends to be higher on weekends.

[16] Golder, S. A., & Macy, M. W. (2011). Diurnal and seasonal mood vary with work, sleep, and daylength across diverse cultures. *Science, 333*(6051), 1878–1881. https://doi.org/10.1126/science.1202775

- **Holiday Effect:** In the 88 countries where national holiday data were available, sentiment is substantially higher on holidays. The effect size corresponds to 58% of daily within-country variation in tweet sentiment, making it a sizable shift. This result is in line with findings that public holidays tend to be associated with more positive social interactions and leisure time, which is reflected in social media sentiment.

Overall, these results suggest that the dataset captures meaningful temporal fluctuations in sentiment, providing reassurance that it reflects real-world mood variations rather than random noise. This strengthens its credibility as a resource for studying broader cross-national patterns in online discourse.

Supplementary Table 16: People tweet more positive messages on the weekend

	Dependent variable:
	Sentiment (z-scored)
Tuesday	−0.06
	(−0.08, −0.03)
Wednesday	−0.05
	(−0.08, −0.03)
Thursday	−0.02
	(−0.05, 0.003)
Friday	0.07
	(0.05, 0.10)
Saturday	0.18
	(0.15, 0.20)
Sunday	0.07
	(0.05, 0.10)
Observations	54,188
Adjusted R^2	0.003
Note:	Brackets include 89% confidence intervals.

Cross-Country Variation in Sentiment Scores

To better understand the structure of sentiment variation, we calculate the intraclass correlation coefficient (ICC), which quantifies how much of the total variance in sentiment is due

Supplementary Table 17: People tweet more positive messages on national holidays

	Dependent variable:
	Sentiment (z-scored)
National holiday (dummy)	0.58
	(0.53, 0.62)
Observations	31,171
Adjusted R^2	0.01
Note:	Brackets include 89% confidence intervals.

to systematic differences between countries versus temporal fluctuations within each country. The ICC estimate is 0.42, meaning that 42% of the total variation in tweet sentiment scores is due to systematic differences between countries, while the remaining 58% is due to variations within each country over time. Substantively, this result underscores that while daily events (e.g., weekday effects) and other time-varying factors play a significant role in shaping sentiment, a substantial portion of the variation is anchored in a country's inherent characteristics. Thus, when interpreting any additional effects (like the influence of the day of the week), it is important to recognize that these operate on top of sizable, persistent differences between countries.

Given this substantial between-country variation, we examine cross-country differences in sentiment by aggregating sentiment scores across the full year. To reduce noise, we exclude countries with fewer than 100 daily tweets on average—a threshold that removes approximately 6.7% of the dataset. After filtering, our final dataset consists of 152 countries.

Predictors of Cross-Country Sentiment Differences To study systematic cross-country differences, we examine four key macro-level predictors: gross domestic product (GDP), democracy, economic inequality, and life satisfaction. Research on subjective well-being suggests that each of these factors is strongly associated with higher self-reported happiness and life satisfaction:

- Wealthier countries tend to have happier citizens, as higher GDP per capita is associated with better living conditions, healthcare, and economic stability[17].

[17] Diener, E., & Biswas-Diener, R. (2002). Will money increase subjective well-being? *Social Indicators Research, 57*(2), 119-169. https://doi.org/10.1023/A:1014411319119

- Democratic countries exhibit greater well-being, as political freedoms, stable governance, and participatory rights enhance overall life satisfaction[18].
- Greater economic inequality is typically linked to lower well-being, as disparities in income contribute to stress, lower social trust, and reduced social cohesion[19].
- Gallup's life satisfaction index is a direct measure of national well-being, summarizing the self-reported happiness of residents[20].

To assess how these factors relate to average national sentiment scores, we compute Pearson's correlation coefficients, reported in Table 18, and visualize scatterplots of these relationships in Figure 30.

Surprisingly, the observed relationships run counter to well-established findings from survey-based life satisfaction research. Whereas Gallup's survey ratings indicate that richer, more democratic, and more equal countries tend to have higher life satisfaction, Twitter sentiment scores show the opposite pattern. Specifically:

- Countries with higher GDP, greater democracy, and higher self-reported life satisfaction actually exhibit lower sentiment scores on Twitter.
- Only economic inequality remains negatively correlated with sentiment, aligning with expectations that greater inequality is associated with lower well-being.

Supplementary Table 18: Correlations between Twitter sentiment and macro indicators

	TSGI Sentiment	Gallup Life stsf	GDP	Lib.democ	Gini
TSGI Sentiment	1	-0.43	-0.27	-0.30	-0.27
Gallup Life stsf	-0.43	1	0.74	0.63	-0.24
GDP	-0.27	0.74	1	0.72	-0.33
Lib.democ	-0.30	0.63	0.72	1	-0.14
Gini	-0.27	-0.24	-0.33	-0.14	1

[18] Helliwell, J. F., Huang, H., & Wang, S. (2014). Social capital and well-being in times of crisis. *Journal of Happiness Studies, 15*(1), 145-162. https://doi.org/10.1007/s10902-013-9441-z

[19] Wilkinson, R., & Pickett, K. (2009). The spirit level: Why more equal societies almost always do better. London: Allen Lane.

[20] Diener, E., Inglehart, R., & Tay, L. (2013). Theory and validity of life satisfaction scales. *Social Indicators Research, 112*(3), 497-527. https://doi.org/10.1007/s11205-012-0076-y

Supplementary Fig. 30: Displaying Twitter sentiment scores against well-established macro predictors of positive affect.

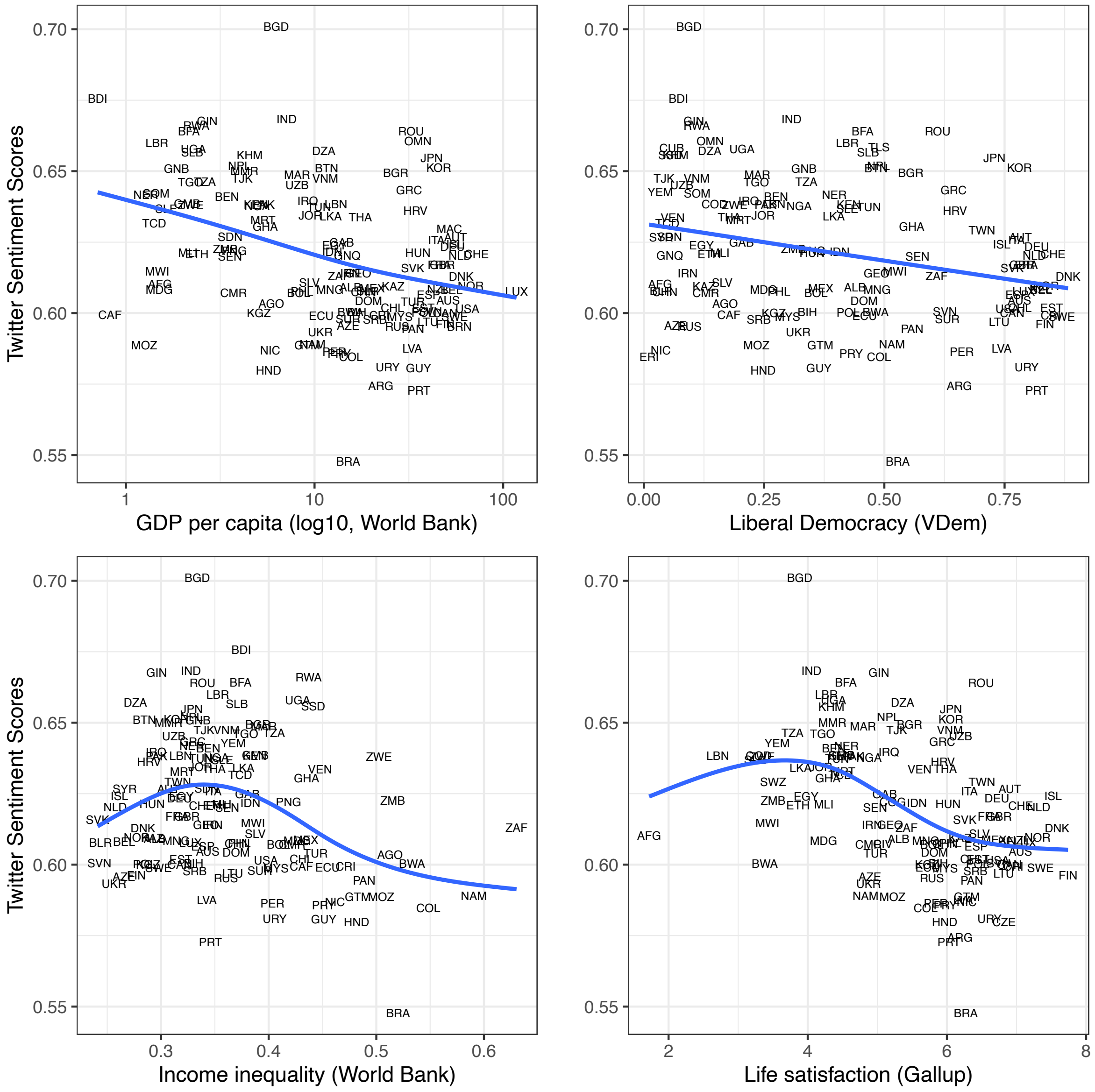

Note: Blue lines are GAM trendlines.

Several factors may explain these unexpected findings. First, despite Twitter's status as a major global platform, its user base remains highly skewed in many non-Western countries, where it is primarily used by a globalized elite rather than a representative sample of the population. Second, as discussed above, only a small fraction of tweets are geo-tagged, and in some countries, a non-trivial proportion of these may come from tourists, whose posts are more likely to be positive, distorting national sentiment estimates. Third, cross-linguistic sentiment analysis remains a highly noisy process, especially when applied to languages for which machine learning models are less well-trained. Even though we limited our analyses to countries with at least 100 tweets per day, the median country-day estimate in our dataset is still based on just 1,823 non-representative tweets, which may be insufficient for reliable estimation.

These limitations reinforce our decision to rely primarily on self-reported measures of hostility. Despite the intuitive appeal of behavioral data, we currently lack both open-access comparative behavioral datasets and sufficiently reliable machine-learning tools for sentiment analysis at scale. Until these challenges are addressed, survey-based approaches remain the most robust method for cross-national research on online hostility.
