# Supplementary material for: Social media users experience more political hostility in less economically equal and less democratic societies
Source: Nat Hum Behav. 2026 Apr 3;10(6):1083–93. doi: 10.1038/s41562-026-02432-5 (PMC13290475; doi:10.1038/s41562-026-02432-5)
Supplement: Supplementary file 2 — Reporting Summary [file 41562_2026_2432_MOESM2_ESM.pdf]

nature portfolio

Corresponding author(s): Alexander Bor
Last updated by author(s): Oct 16, 2025

Reporting Summary

Nature Portfolio wishes to improve the reproducibility of the work that we publish. This form provides structure for consistency and transparency in reporting. For further information on Nature Portfolio policies, see our Editorial Policies and the Editorial Policy Checklist.

Statistics

For all statistical analyses, confirm that the following items are present in the figure legend, table legend, main text, or Methods section.

n/a	Confirmed	
☐	☒	The exact sample size (*n*) for each experimental group/condition, given as a discrete number and unit of measurement
☐	☒	A statement on whether measurements were taken from distinct samples or whether the same sample was measured repeatedly
☐	☒	The statistical test(s) used AND whether they are one- or two-sided<br>*Only common tests should be described solely by name; describe more complex techniques in the Methods section.*
☐	☒	A description of all covariates tested
☒	☐	A description of any assumptions or corrections, such as tests of normality and adjustment for multiple comparisons
☐	☒	A full description of the statistical parameters including central tendency (e.g. means) or other basic estimates (e.g. regression coefficient) AND variation (e.g. standard deviation) or associated estimates of uncertainty (e.g. confidence intervals)
☒	☐	For null hypothesis testing, the test statistic (e.g. *F*, *t*, *r*) with confidence intervals, effect sizes, degrees of freedom and *P* value noted<br>*Give P values as exact values whenever suitable.*
☐	☒	For Bayesian analysis, information on the choice of priors and Markov chain Monte Carlo settings
☐	☒	For hierarchical and complex designs, identification of the appropriate level for tests and full reporting of outcomes
☐	☒	Estimates of effect sizes (e.g. Cohen's *d*, Pearson's *r*), indicating how they were calculated

Our web collection on statistics for biologists contains articles on many of the points above.

Software and code

Policy information about availability of computer code

Data collection	Data was collected using YouGov's native survey platform.
Data analysis	R version 4.2.1 (2022-06-23)<br>Platform: x86_64-apple-darwin17.0 (64-bit)<br>Running under: macOS Monterey 12.6.1<br>attached base packages:<br>[1] stats graphics grDevices utils datasets methods base<br><br>other attached packages:<br>[1] mice_3.15.0 Hmisc_4.7-1 Formula_1.2-4 survival_3.3-1 lattice_0.20-45<br>[6] ggokabeito_0.1.0 patchwork_1.1.2 ggridges_0.5.4 tidybayes_3.0.2 here_1.0.1<br>[11] brms_2.18.0 Rcpp_1.0.11 broom_1.0.1 rio_0.5.29 forcats_0.5.2<br>[16] stringr_1.4.1 dplyr_1.1.2 purrr_1.0.1 readr_2.1.3 tidyr_1.2.1<br>[21] tibble_3.2.1 ggplot2_3.4.2 tidyverse_1.3.2

For manuscripts utilizing custom algorithms or software that are central to the research but not yet described in published literature, software must be made available to editors and reviewers. We strongly encourage code deposition in a community repository (e.g. GitHub). See the Nature Portfolio guidelines for submitting code & software for further information.

Data

Policy information about availability of data

All manuscripts must include a data availability statement. This statement should provide the following information, where applicable:

- Accession codes, unique identifiers, or web links for publicly available datasets
- A description of any restrictions on data availability
- For clinical datasets or third party data, please ensure that the statement adheres to our policy

All materials, data and code are deposited on the OSF at https://osf.io/9r4vs/?view_only=0701044010934367bb96783678befaba

Research involving human participants, their data, or biological material

Policy information about studies with human participants or human data. See also policy information about sex, gender (identity/presentation), and sexual orientation and race, ethnicity and racism.

Reporting on sex and gender	We rely on self-reported data on gender provided by YouGov. We report the gender composition of each of our 30 samples in Table A1. in the online appendix.
Reporting on race, ethnicity, or other socially relevant groupings	N/A
Population characteristics	See above
Recruitment	Respondents were recruited from YouGov's standing panels.
Ethics oversight	The study design was approved by Aarhus University's Research Ethics Committee (BSS-2022-113)

Note that full information on the approval of the study protocol must also be provided in the manuscript.

Field-specific reporting

Please select the one below that is the best fit for your research. If you are not sure, read the appropriate sections before making your selection.

☐ Life sciences ☒ Behavioural & social sciences ☐ Ecological, evolutionary & environmental sciences

For a reference copy of the document with all sections, see nature.com/documents/nr-reporting-summary-flat.pdf

Behavioural & social sciences study design

All studies must disclose on these points even when the disclosure is negative.

Study description	We report survey data from 30 countries: Algeria, Argentina, Australia, Belgium, Brazil, Colombia, Denmark, Egypt, France, Germany, Hungary, Indonesia, Iraq, Ireland, Malaysia, Mexico, Morocco, Netherlands, Norway, Pakistan, Philippines, Poland, Singapore, Slovakia, Sweden, Switzerland, Thailand, Turkey, United Arab Emirates, United States
Research sample	Our sample consists of online respondents from 30 countries. Given that we are primarily interested in people's online behavior this is an appropriate participant pool. Online surveys allow a relatively cost effective method for collecting data from diverse populations across the world.
Sampling strategy	Respondents were quota sampled by YouGov from their national pools of panelists. Our primary constrain had been money. That said we performed and preregistered design calculations to ensure that our sample will be informative for our hypotheses.
Data collection	YouGov recruits respondents through survey links, which respondents fill out on their own internet-enabled deviced at their own environment in a double-blind manner.
Timing	Data were collected between April 14 and July 07, 2023. County-level data collection dates are reported in Table B1 in the online appendix.
Data exclusions	Respondents who indicated using no Social Media accounts or who failed a very simple attention check at the beginning of the survey were screen out, as preregistered. No participant who finished the survey was excluded.
Non-participation	YouGov provides no data on response rates. For questions that we deemed sensitive, we offered participants an NA option. Figure A1 in the online appendix shows that the share of respondents who were not willing to report e.g. their frequency of talking about politics was negligible.
Randomization	Our study is fundamentally observational. Yet, we do randomize the order of the two key variables: self-reported online and offline

Randomization	hostility (which appear either at the top or bottom of our survey). We relied on YouGov's native randomization algorithms to do the randomization, and report "experimental" results in Online Appendix M.

Reporting for specific materials, systems and methods

We require information from authors about some types of materials, experimental systems and methods used in many studies. Here, indicate whether each material, system or method listed is relevant to your study. If you are not sure if a list item applies to your research, read the appropriate section before selecting a response.

Materials & experimental systems

n/a	Involved in the study
☒	☐ Antibodies
☒	☐ Eukaryotic cell lines
☒	☐ Palaeontology and archaeology
☒	☐ Animals and other organisms
☒	☐ Clinical data
☒	☐ Dual use research of concern
☒	☐ Plants

Methods

n/a	Involved in the study
☒	☐ ChIP-seq
☒	☐ Flow cytometry
☒	☐ MRI-based neuroimaging

Plants

Seed stocks	n/a
Novel plant genotypes	n/a
Authentication	n/a
