# Supplementary material for: Social media users experience more political hostility in less economically equal and less democratic societies
Source: Nat Hum Behav. 2026 Apr 3;10(6):1083–93. doi: 10.1038/s41562-026-02432-5 (PMC13290475; doi:10.1038/s41562-026-02432-5)
Supplement: Supplementary file 3 — Peer Review File [file 41562_2026_2432_MOESM3_ESM.pdf]

nature portfolio

Peer Review File

Social media users experience more political hostility in less economically equal and less democratic societies

Corresponding Author: Dr Alexander Bor

Version 0:

Decision Letter:

15th July 2024

Dear Dr Bor,

Thank you once again for your manuscript, entitled "Undemocratic and unequal countries experience more political hostility on social media -- Evidence from 30 Countries", and for your patience during the peer review process.

Your Article has now been evaluated by 3 referees. You will see from their comments copied below that, although they find your work of potential interest, they have raised quite substantial concerns. In light of these comments, we cannot accept the manuscript for publication, but would be interested in considering a revised version if you are willing and able to fully address reviewer and editorial concerns.

We hope you will find the referees' comments useful as you decide how to proceed. If you wish to submit a substantially revised manuscript, please bear in mind that we will be reluctant to approach the referees again in the absence of major revisions. We are committed to providing a fair and constructive peer-review process. Do not hesitate to contact us if there are specific requests from the reviewers that you believe are technically impossible or unlikely to yield a meaningful outcome.

To guide the scope of the revisions, the editors discuss the referee reports in detail within the team, including with the chief editor, with a view to (1) identifying key priorities that should be addressed in revision and (2) overruling referee requests that are deemed beyond the scope of the current study. We hope that you will find the prioritised set of referee points to be useful when revising your study. Please do not hesitate to get in touch if you would like to discuss these issues further. In particular, we would ask you that:

(1) Reviewers 1 and 2 highlight that the current evidence is not sufficient to demonstrate the claimed mechanistic relationship between social media hostility and democracy/equality. This is important to demonstrate (with additional evidence). It is equally important to exclude alternative explanations.

(2) The reviewers raise concerns about the measure of hostility used in your manuscript, as it confounds observations of hostility online and personal victimization. Relatedly, Reviewers 1 and 2 are particularly concerned about the exclusive reliance on self-reported measures, which are known to be unreliable. Please clarify the construct of hostility your measure is intended to capture and supplement your self-report data with an automated measure of hostility from online text, as recommended by Reviewer 2. Finally, please formally demonstrate the invariance of measurement, per Reviewer 1’s request.

(3) Please improve the theoretical reasoning of hypotheses and the discussion of your findings (Reviewers 1 and 3).

Finally, your revised manuscript must comply fully with our editorial policies and formatting requirements. Failure to do so will result in your manuscript being returned to you, which will delay its consideration. To assist you in this process, I have attached a checklist that lists all of our requirements. If you have any questions about any of our policies or formatting, please don't hesitate to contact me.

If you wish to submit a suitably revised manuscript, we would hope to receive it within 4 months. I would be grateful if you could contact us as soon as possible if you foresee difficulties with meeting this target resubmission date.

With your revision, please:

• Include a “Response to the editors and reviewers” document detailing, point-by-point, how you addressed each editor and referee comment. If no action was taken to address a point, you must provide a compelling argument. When formatting this document, please respond to each reviewer comment individually, including the full text of the reviewer comment verbatim followed by your response to the individual point. This response will be used by the editors to evaluate your revision and sent back to the reviewers along with the revised manuscript.

• Highlight all changes made to your manuscript or provide us with a version that tracks changes.

Please use the link below to submit your revised manuscript and related files:

Link Redacted

Note: This URL links to your confidential home page and associated information about manuscripts you may have submitted, or that you are reviewing for us. If you wish to forward this email to co-authors, please delete the link to your homepage.

Thank you for the opportunity to review your work. Please do not hesitate to contact me if you have any questions or would like to discuss the required revisions further.

Sincerely,

[redacted]
Nature Human Behaviour

Reviewer expertise:

Reviewer #1: communication, research methodology

Reviewer #2: online communication, computational social science, Bayesian approach

Reviewer #3: political communication, public opinion

REVIEWER COMMENTS:

Reviewer #1:
Remarks to the Author:
The present research focuses on the role of contextual factors that increase the prevalence of hostility on social media. Specifically, the paper argues and shows that online hostility is more prevalent in undemocratic countries and is mostly due to status seeking individuals. The authors present a reasonable argument and present data from 30 countries to support their assumptions. Although I appreciate the effort of the authors to make a convincing case, I have several concerns that make me reluctant to recommend the manuscript for publication in Nature Human Behavior. These concerns refer to the theorizing, but especially to the methods and measurement. The methodological issues should be definitely addressed since they are a serious problem and can impede the validity of the findings.
Theory/Rationale
The authors argue that hostility is a result of people trying to overcome inequality (be it political and/or economic). This should be more likely in poor and undemocratic countries. This makes political and/or economic inequality the proximal cause and level of democracy a distal cause. I do not understand how the authors can disentangle these interdependent relationships and formulate 3 hypotheses. These hypotheses cannot be independently tested since the variables are confounded. Can the authors clarify?
The theory section suggests that the paper is about the personal experience of hostility. However, given the specific measurement it becomes clear that the study cannot disentangle people's observations of hostility online and personal victimization. The self-report questions leave a lot of room for interpretation: "I saw content that ridiculed people like me", "I saw content that threatened or harassed someone like me". It is unclear whether people agreeing that this happened to them in last 30 days were truly victims themselves. The validation of this measure at the country level does not help very much since there may alternative explanations for the prevalence of hostility. There are several alternative explanations that can interfere or invalidate the conclusions that the authors draw from their findings. For instance, differences in findings may be also due to the fact that moderation practice on platforms is more professional in richer /more democratic countries compared to poorer/undemocratic countries. This would result in higher prevalence of online hostility regardless of status seeking motivations or level of inequality. It is well known that platforms do a better moderation job in some countries versus others.
Methods: The authors argue that the survey is representative of the national OR online populations. I am wondering how the authors can know the online population of a given country. Do the authors compare demographics with census data to show that samples are representative in terms of selected traits? Is the sampling comparable across countries? I doubt it since internet penetration rates differ considerably. This makes the comparison of samples across countries a complicated issue. How do the authors justify the sample size of 500 per country? Is that based on a power analysis?
The authors relied on self-reported measures of hostility and argued that this was a valid measure. They refer to a study in which it is stated that "Beliefs about what hostile speech is likely to fluctuate across individuals and national contexts". I would also assume that users in countries with a higher exposure to online and offline hostility become desensitized and respond differently to self-report measures of hostility. Thus, self-report measures would not be a valid indicator in the present study. Another, yet related issue, is the invariance of measurement that needs to be demonstrated, e.g., by means of confirmatory factor analysis. If measurements are not invariant comparisons across populations become problematic because differences in means or correlations may be due to differences in measurement and do not represent meaningful differences or correlations. Differences in reliabilities in the methods section indicate a lack of measurement invariance that should be tested using formal tests.
It is unclear what the use of the "novel, seven-item survey measure to capture the three dominant narratives of social media's impact on politics" should be. There is no theory that explains the role of this variable and how it is related to perception of hostility.
Results: Can the authors say anything about the size of the effects? What does a regression coefficient between .025 and .05 mean? When the authors look at platform differences their interpretation is that there are only small differences. Transferred to the regression coefficients: are these effects meaningful?

Findings related to anomia and polarization: The authors demonstrate that anomie and polarization are not related to the experience of online hostility. Although this is an important finding, the more important result would be to formally test whether these effects are different from the effects that were obtained. Thus, do the effects that were obtained are different from the non-significant results on anomia and polarization.

Reviewer #2:
Remarks to the Author:
In their manuscript "Undemocratic and unequal countries experience more political hostility on social media – Evidence from 30 Countries" the authors describe the results of a large survey about self-reported experiences with political hostility online and also offline.
The work makes an interesting contribution to an important topic and the main value is the global perspective that is provided here. I think some conclusions from the data are warranted and important, most interestingly the interaction between individual factors, such as offline hostility or status seeking, and the country-level factors. Those results potentially contribute to the picture of social media making certain groups particularly visible, namely the unsatisfied with the system. Those groups might be smaller in more democratic countries and broader in less democratic countries, and thereby either causing more turmoil in the "functioning" systems and having a very similar effect in less "functioning" systems, but where the turmoil is justified and rather materialized and seen as liberation.
Other conclusions are less justified by the data in my view, namely that social media platforms and algorithms play a little role in those behaviours, because the survey is neither specific in the type of platforms that are used nor how platforms co-evolve with their users, which can lead to different outcomes in different countries but not excluding the possibility that the same mechanisms are at play (see above, making the dissatisfied be more visible in a system, for example). The other conclusion, that shines through the manuscript and is not supported by the survey data, in my view, is the causal direction of people being status seeking or hostile offline leading them to be hostile online, the mechanism could also work the other way, i.e., people radicalizing online and carrying this hostility offline, cross-sectional data just cannot distinguish that. Lastly, the self-reported measures may have an advantage of comparability due to language-barriers of e.g., automated toxicity detection across languages, but it also comes with a downside that self-reporting on online behavior is not very reliable (see https://www.nature.com/articles/s41562-021-01117-5). This could potentially be compensated by backing the hostility prevalence data with some automated measure of hostility from online text (e.g., comment sections), in addition.
Those are my main concerns that need to be resolved in my view, so to make this a contribution that adds to our understanding of the global effects of social media use (which is mostly the aspect of empowering the unsatisfied, that can be a liberating or destabilizing thing, depending on the system). I would be interested to hear from the authors about this and to read a revised version of the manuscript that makes the scope of the contribution clearer.

a few more specific questions:
- is there data on the platforms that people mostly use in the respective countries?
- are there other country-level attributes that can be used to describe online victimhood?
- the problems of self-reported hostility are probably affected by demand characteristics, correct?

Reviewer #3:
Remarks to the Author:
This study uses self-reported survey data from 30 countries to examine sociopolitical context and factors that may play a role in the developing hostility – thus going against the social science (and public discourse) that blames much of recent sociopolitical hostility on social media platforms. It is a much-welcome intervention into this space, which has been so dominated by a convenient but not empirically supported boogeyman. The authors argue that online hostility may be driven by a small set of status-seeking individuals, who may be just as likely to engage in offline hostility, and who may be particularly apt to surface in contexts that suffer democratic threats, including inequality. Across this well-done study, the authors provide a strong argument for how we should think of the role of social media in fomenting our political ills – and a clarion call for including all-important sociopolitical context when analyzing those relationships. Political context – and culture – matter.

A note: this study finds that a likely small set of status-seeking individuals may be responsible for driving online hostility – see also the work on repeat offenders, the "dirty dozen", elite disinformation, below:
https://252f2edd-1c8b-49f5-9bb2-cb57bb47e4ba.filesusr.com/ugd/f4d9b9_b7cedc0553604720b7137f8663366ee5.pdf
https://www.cip.uw.edu/2023/10/20/new-elites-twitter-x-most-influential-accounts-hamas-israel/
Tripodi, F. B. (2022). The propagandists' playbook: How conservative elites manipulate search and threaten democracy. Yale University Press.

The manuscript states, "Finally, we find that people in more undemocratic countries, despite more experiences with hostile online debates, are more likely to emphasize the liberating potential of social media." This aligns with a recent Knight & Gallup study that found Black Americans were more likely to engage in offline political activities because of online political discussions, despite being endemically marginalized from democratic processes within the country (US). https://knightfoundation.org/reports/media-and-democracy/

The manuscript states, "Importantly, the returns to individuals who are motivated to dominate are particularly high in sociocultural contexts that are characterized by various forms of inequality." I hope the US is considered in this case – AND its uniqueness, thought similar to other western democratic countries, of the hostility coming from those who do actually possess power – often by upholding systems of oppression – but nonetheless *feel* they are the ones impacted by inequalities.

How do the authors feel about the measure of victimhood not accurately reflecting true targets of hostility? As in, for example, a cis

straight white man feels a gay-pride post is hostile. Normatively, there is no victim here – the cis straight white male still holds exceptionally more power than any queer person.

Of figure 1 – can you note that the scale and key, I assume, is for reported victimhood of online political hostility?

On polarization, the manuscript states, “We sought to benchmark the role of status-driven risk taking and meaninglessness to a popular psychological predictor, namely affective polarization. Surprisingly, we found no evidence for a relationship between polarization and political hostility either.” Perhaps worth incorporating this recent critique of polarization work to contextualize this finding: Kreiss, D., & McGregor, S. C. (2024). A review and provocation: On polarization and platforms. New Media & Society, 26(1), 556-579.

The study includes this fascinating finding: “Individuals high in status-driven risk taking are more hostile if they live in a more democratic or – to a lesser extent – in an unequal country.” There are many possible interpretations for this, but one likely one is because the status-seeking individuals would rather live in a less democratic country where they can take advantage of their relatively high status within an unequal society.

The manuscript states, “In democracies and economically equal countries, online hostility is, in contrast, more exclusively driven by the usual suspects of aggression: young male status seekers.” Might be worth tying to some of the work that examines their particular role in social media (e.g., the manosphere, trolling, incels, etc.)

I’d suggest giving an example or two of the “usual suspects” references here: “In undemocratic or unequal countries – even if they suffer more from online hostility overall – the same connectivity may liberate a broader public disenfranchised from their society, as we found engagement in hostility beyond the ‘usual suspects’ in these countries.”

Just wanted to give a huge amen to this, in the manuscript: “Decision makers’ current efforts to regulate tech giants can have beneficial effects, but they are unlikely to resolve the social tensions reflected on social media. Instead, politicians need to “fix society”: to face the possibility that to truly improve the climate of online political discussions, it will be necessary to make societies more democratic, more equal, and more harmonious offline.”

Were the sample medians (e.g., for age), calculated per country or across the whole multi-country sample?

Version 1:

Decision Letter:

23rd April 2025

Dear Dr Bor,

Thank you once again for your revised manuscript, entitled "Undemocratic and unequal countries experience more political hostility on social media," and for your patience during the re-review process.

Your manuscript has now been evaluated by the same reviewers who evaluated your original manuscript. All reviewer feedback is included at the end of this letter. Although the reviewers found your manuscript to have improved during revision, they also raise some important outstanding concerns. We remain interested in the possibility of publishing your study in Nature Human Behaviour, but would like to consider your response to these outstanding concerns in the form of a revised manuscript before we make a decision on publication.

As you will see, Reviewer 2 continues to maintain substantial reservations about the causal/mechanistic framing and claims/interpretations in your discussion. These are concerns we share. We appreciate that you preregistered directional hypotheses; however, what has become clear during the peer review process is that the hypotheses cannot be unequivocally tested by your data. Nonetheless, like Reviewer 2, we believe that a descriptive presentation of your work would still be valuable, accompanied by a discussion of alternative potential mechanisms with which your data are consistent. It is still important to present your hypotheses in the introduction (given that you preregistered them) - however, it is equally important to be clear from the outset that your data cannot provide unequivocal tests of these hypotheses and that you discuss alternative mechanisms. To be clear, the changes required are not merely 'cosmetic' - how your work is presented needs to be fundamentally reconceptualized in order to be suitable for publication in Nature Human Behaviour.

Finally, your revised manuscript must comply fully with our editorial policies and formatting requirements. Failure to do so will result in your manuscript being returned to you, which will delay its consideration. To assist you in this process, I have attached a checklist that lists all of our requirements. If you have any questions about any of our policies or formatting, please don't hesitate to contact me.

In sum, we invite you to revise your manuscript taking into account all reviewer and editor comments. We are committed to providing a fair and constructive peer-review process. Do not hesitate to contact us if there are specific requests from the reviewers

that you believe are technically impossible or unlikely to yield a meaningful outcome.

We hope to receive your revised manuscript within 4-8 weeks. I would be grateful if you could contact us as soon as possible if you foresee difficulties with meeting this target resubmission date.

With your revision, please:

• Include a "Response to the editors and reviewers" document detailing, point-by-point, how you addressed each editor and referee comment. If no action was taken to address a point, you must provide a compelling argument. This response will be used by the editors and reviewers to evaluate your revision.

• Highlight all changes made to your manuscript or provide us with a version that tracks changes.

Please use the link below to submit your revised manuscript and related files:

Link Redacted

Note: This URL links to your confidential home page and associated information about manuscripts you may have submitted, or that you are reviewing for us. If you wish to forward this email to co-authors, please delete the link to your homepage.

We look forward to seeing the revised manuscript and thank you for the opportunity to review your work. Please do not hesitate to contact me if you have any questions or would like to discuss these revisions further.

Sincerely,

[redacted]
Nature Human Behaviour

Reviewer expertise:

Reviewer #1: communication, research methodology

Reviewer #2: online communication, computational social science, Bayesian approach

Reviewer #3: political communication, public opinion

REVIEWER COMMENTS:

Reviewer #1 (Remarks to the Author):

I appreciate the effort that the authors put into revising the manuscript. In this revision, they have addressed nearly all of my concerns in a very convincing way, either by providing additional empirical evidence to support their argument or by offering clarifications and explanations that enhance the understanding of their argument.
One final issue that remains pertains to the findings at the end of the paper. I still believe that the last part of the paper is not well integrated and lacks specificity. The discussion around perceptions of threats or narratives (liberation, turmoil, oppression) remains unclear, and the clarifications in the revised manuscript did not help alleviate my concerns. This issue becomes apparent when examining the hypotheses: the findings related to perceived online hostility are based on hypotheses that are sufficiently theorized, and all headings are affirmative in nature. The section on perceptions of the impact of social media begins with a question and lacks expectations regarding how specific contexts or predictors may influence such perceptions. The justification that a single paper provided a "structured framework" for formulating survey items is weak. It is unclear what these items truly measure and whether they form a valid and reliable scale. Developing a news scale requires more than just one inspiring paper. Methods books outline the extensive process required to validate a scale and establish construct validity, steps that are absent from the present manuscript. For example, did the items survive a confirmatory factor analysis? Do the scales correlate as expected with constructs that should be related to these scales? Are these scales invariant across cultural contexts? I suggest removing this weaker section from the manuscript, as I believe the paper is stronger without it, but I defer that decision to the editor.

Reviewer #2 (Remarks to the Author):

I thank the authors for their response and the revision of the manuscript. Unfortunately, I still see the existing concerns that I raised initially, which mostly boils down to claims and conclusions that are not fully supported by the presented data. I do find the dataset still very interesting and valuable and I agree with some of the conclusions, e.g., that the role of social media may be different depending on the social- and political context. However, the present data does not allow for specific mechanisms like "Social media may thus have provided democratic audiences with a window into minority frustrations that hitherto went unnoticed.". It could

as well be that the effects of social media interact with country-level factors. If I read the manuscript correctly, the main message is that social media is merely a mirror of society and not a driver, which I don't see can be confidently argued with the data at hand. In my view the narrative of the paper needs to be thoroughly revised to report the descriptive data in a neutral way and discuss different potential mechanisms behind the observations in parallel. The current presentation of the manuscript makes it additionally difficult to understand the (slightly mechanistic) argumentation of the paper that is based on a combination of various cross-sectional descriptive results.

Let me explain in more detail:

For example, an argument I disagree with is stated in the introduction of the revised manuscript as "If online hostility were solely a product of the technological affordances of global social media platforms, one would expect similar levels of hostility across countries, with only minor random variation."
This is only true if one would assume purely homogeneous effects of social media and no interactions with individual factors, which is indeed a naive view that is not propagated in the scientific discussion.

Relatedly, the fact that online and offline hostility are correlated is not a proof for selection effects, those behaviours can influence each other in both ways. There is evidence for online hostility leading to offline hate crimes, for example.

How does the idea that status-seeking risk taking is a stable psychological trait fit with the finding that such individuals are "more numerous in undemocratic countries"? The authors themselves write later that this behaviour may depend on social/economic factors, hence it can well be that status-seeking is influenced by online hostility, too.

Relatedly, here the authors don't provide a reference to prior research: "While our data cannot establish causality, theory and prior research suggest that status-driven risk-taking, as a stable trait, is more likely to drive online hostility than be shaped by it." As this sentence carries a strong assumption about the independence of certain behaviour from the environment, it would be important.

Furthermore, in the discussion the authors argue: "our findings offer an important corrective to conventional wisdom about the origins of hostility in online discussions on societal matters", which again is suggesting a causal order that I can't read in the data. In addition, I am not sure what conventional wisdom the authors are arguing against, at least in the scientific literature.

Same here: "Our findings, however, clearly show that online hostility is not just an accident or an artifact of well-meaning individuals' corruption by technology." It is very difficult to show these things and usually requires controlled- or natural experiments or some temporal dimension. The descriptive data is not a problem in itself, but it should not be matched with such strong statements.

Further down in the discussion section the authors say: "Our cross-cultural data challenge the overly pessimistic narrative about social media's impact on society that prevails in the West. Policy, journalistic and academic narratives in the U.S. and Europe often single out social media as uniquely responsible for fueling affective polarization, spreading fake news and conspiracy theories, and harming mental health." If the conclusion here would be that online hostility shows remarkable heterogeneity across political contexts, I would totally agree, but I feel it is a bit of a strawman to present the findings as "challenge overly pessimistic narratives" and implies that the data rules out some of those potential effects.

In addition I still find it very hard to follow the Hypothesis in connection to the preregistration as the numbering has completely changed, maybe a table in the methods section that states which have been confirmed and which have not would help a lot.

Furthermore, in the follow-up study 4 I don't see why the effects of narrative only go in one direction and why this would mean that the cross-country differences reported here are a lower bound, please expand.

As I said, I would appreciate a paper that reports the differences of online hostility across countries, the individual-level correlates and discuss potential mechanisms behind that. I am a huger supporter of descriptive work, but in the current form the narrative around it is too strong and does not discuss alternative explanations.

Reviewer #2 (Remarks on code availability):

I only had a brief look at the code and it seems well documented and I could follow the code behind the main figures. The data will be a valuable resource for the community.

Reviewer #3 (Remarks to the Author):

The authors have done a thorough job responding to comments from the reviewers, as well as from the editors. It is especially good that they've made it even more clear that their aim is to be descriptive, which is welcome in this regard.

I agree with the authors that the hostility measures in this case cannot be supplemented with scraped data as observed behavioral measures from platforms. Furthermore, I support the authors' contention that the self-reported measures the study employs

purposively measure whether people "fee" offended or affected by hostile messages, which is most in line with the study's intent to capture perceptions of group-based victimization, which is of most concern in the democratic sense. This also captures then hostility from those with status, in unequal contexts, to those seeking equal status.

I'm glad to see the authors address the normative aspect briefly, though the descriptives from the MRP analysis that they provided in the response letter (that shows young men with low education and minorities report more hostilities) is a good example to highlight the point they make in the text.

Version 2:

Decision Letter:

Our ref: NATHUMBEHAV-24041490B

21st August 2025

Dear Dr. Bor,

Thank you for submitting your revised manuscript "Undemocratic and unequal countries experience more political hostility on social media" (NATHUMBEHAV-24041490B). It has now been seen by the original referee and their comments are below. As you can see, the reviewer find that the paper has improved in revision. We will therefore be happy in principle to publish it in Nature Human Behaviour, pending minor revisions to satisfy the referees' final requests and to comply with our editorial and formatting guidelines.

We are now performing detailed checks on your paper and will send you a checklist detailing our editorial and formatting requirements within three weeks. Please do not upload the final materials and make any revisions until you receive this additional information from us.

Please do not hesitate to contact me if you have any questions.

Sincerely,

[redacted]
Nature Human Behaviour

Reviewer #2 (Remarks to the Author):

First, I want to say sorry for my delayed evaluation. Second, I am satisfied with the adjustments that the authors made around the causal language. Also, I am happy with the discussions of the complexity of social media effects, specifically the fact that feedback loops are involved between social media and broader society. Overall, the study adds now further data to our understanding of those complex interplays.

Reviewer #2 (Remarks on code availability):

I did have a look at the data and code in the last round.

Version 3:

Decision Letter:

Dear Dr Bor,

We are pleased to inform you that your Article "Social media users experience more political hostility in less economically equal and less democratic societies", has now been accepted for publication in Nature Human Behaviour.

Authors may need to take specific actions to achieve compliance with funder and institutional open access mandates. If your research is supported by a funder that requires immediate open access (e.g. according to Plan S principles or the NIH public access policy) then you should select the gold OA route, and we will direct you to the compliant route where possible. Because authors warrant under our

subscription licensing terms that they haven't committed to licensing any version of their article under a licence inconsistent with the terms of our agreement – including the applicable embargo period – publication under the subscription model isn't suitable for authors whose funders require no embargo.

Once your manuscript is typeset and you have completed the appropriate grant of rights, you will receive a link to your electronic proof via email with a request to make any corrections within 48 hours. If, when you receive your proof, you cannot meet this deadline, please inform us at rjsproduction@springernature.com immediately. Once your paper has been scheduled for online publication, the Nature press office will be in touch to confirm the details.

Acceptance of your manuscript is conditional on all authors' agreement with our publication policies (see http://www.nature.com/nathumbehav/info/gta). In particular your manuscript must not be published elsewhere and there must be no announcement of the work to any media outlet until the publication date (the day on which it is uploaded onto our web site).

If you have posted a preprint on any preprint server, please ensure that the preprint details are updated with a publication reference, including the DOI and a URL to the published version of the article on the journal website.

An online order form for reprints of your paper is available at https://www.nature.com/reprints/author-reprints.html. All co-authors, authors' institutions and authors' funding agencies can order reprints using the form appropriate to their geographical region.

We welcome the submission of potential cover material (including a short caption of around 40 words) related to your manuscript; suggestions should be sent to Nature Human Behaviour as electronic files (the image should be 300 dpi at 210 x 297 mm in either TIFF or JPEG format). Please note that such pictures should be selected more for their aesthetic appeal than for their scientific content, and that colour images work better than black and white or grayscale images. Please do not try to design a cover with the Nature Human Behaviour logo etc., and please do not submit composites of images related to your work. I am sure you will understand that we cannot make any promise as to whether any of your suggestions might be selected for the cover of the journal.

You can now use a single sign-on for all your accounts, view the status of all your manuscript submissions and reviews, access usage statistics for your published articles and download a record of your refereeing activity for the Nature journals.

To assist our authors in disseminating their research to the broader community, our SharedIt initiative provides you with a unique shareable link that will allow anyone (with or without a subscription) to read the published article. Recipients of the link with a subscription will also be able to download and print the PDF.

As soon as your article is published, you will receive an automated email with your shareable link.

In approximately 10 business days you will receive an email with a link to choose the appropriate publishing options for your paper and our Author Services team will be in touch regarding any additional information that may be required.

If you have any questions about our publishing options, costs, Open Access requirements, or our legal forms, please contact ASJournals@springernature.com

We look forward to publishing your paper.

With best regards,

[redacted]
Nature Human Behaviour

P.S. Click on the following link if you would like to recommend Nature Human Behaviour to your librarian http://www.nature.com/subscriptions/recommend.html#forms

** Visit the Springer Nature Editorial and Publishing website at www.springernature.com/editorial-and-publishing-jobs for more information about our career opportunities. If you have any questions please click here.**

Open Access This Peer Review File is licensed under a Creative Commons Attribution 4.0 International License, which permits use, sharing, adaptation, distribution and reproduction in any medium or format, as long as you give appropriate credit to the original author(s) and the source, provide a link to the Creative Commons license, and indicate if changes were made.
In cases where reviewers are anonymous, credit should be given to 'Anonymous Referee' and the source.
The images or other third party material in this Peer Review File are included in the article's Creative Commons license, unless indicated otherwise in a credit line to the material. If material is not included in the article's Creative Commons license and your intended use is not permitted by statutory regulation or exceeds the permitted use, you will need to obtain permission directly from the copyright holder.
To view a copy of this license, visit https://creativecommons.org/licenses/by/4.0/

- We found a small anchoring bias, but only from a high anchor, indicating that our hostility measure may underestimate differences across countries (i.e., our tests are too conservative). (Follow-up study 4)

Please clarify the construct of hostility your measure is intended to capture …

In our revision, we clarified (and demonstrated) that we measure **group-based** victimization, i.e., victimization of group members the user identifies with. This is an important feature of our design, as it builds on prior research demonstrating that it is specifically the high levels of group-based (versus personal) victimization which is unique about online political discussions. Prior research shows that relatively few individuals are victims of personal attacks online, and this minority also has many conflicts in political discussions that happen off the internet. The unique feature of the online sphere is that people encounter a lot of hostile content online aimed either at other individuals or at groups they identify with (Bor & Petersen, 2022). An overly narrow measure focused at personal victimization would therefore risk underestimating exposure to content which individuals find psychologically harmful.

We conducted follow up study 1 to test the implications of this design choice, with the goal of gauging the sensitivity of our measure of victimhood to who is the target of hostility (ego vs ingroup vs anyone). We found that our measure specifically captures group-based victimization, distinguishing it from general observations of hostility and direct personal victimization.

… and supplement your self-report data with an automated measure of hostility from online text, as recommended by Reviewer 2.

We appreciate the reviewers' recommendation to supplement our data with automated behavioral measures, and we have carefully evaluated the feasibility and potential benefits of doing so. While we recognize the intuitive appeal of using scraped text data, we have concluded that such an approach is unlikely to generate cross-cultural measures of hostility that improve on our self-reports, for two main reasons:

1) **Lack of representative cross-country social media data.** To the best of our knowledge, there are no existing datasets or methodologies for sampling behavioral data that could be plausibly considered to be representative of (or at least resembling) the national online discussion environments. The most popular global platforms (e.g. Facebook, Instagram) do not offer data access to researchers. Meanwhile, comparisons across smaller platforms run into issues of sampling bias. Imagine we found that there is more hostility around current discussions about Greenland in the US than in Denmark on X. Would it be prudent to attribute this to country differences in average levels of hostility? Or would it be because different parts of society are drawn to this topic in the US (hawkish Republicans) than in Denmark (an ideologically diverse crowd)? Or would it be because X plays a very different role in the US online ecosystem than in Denmark (where it is less widely used)? It would be impossible to tell.
2) **Lack of valid machine-learning methods for cross-cultural hostility detection.** Even if we had comparable data from multiple countries, it is unclear how we could measure

REVIEWER COMMENTS:

Reviewer #1:

The present research focuses on the role of contextual factors that increase the prevalence of hostility on social media. Specifically, the paper argues and shows that online hostility is more prevalent in undemocratic countries and is mostly due to status seeking individuals. The authors present a reasonable argument and present data from 30 countries to support their assumptions. Although I appreciate the effort of the authors to make a convincing case, I have several concerns that make me reluctant to recommend the manuscript for publication in Nature Human Behavior. These concerns refer to the theorizing, but especially to the methods and measurement. The methodological issues should be definitely addressed since they are a serious problem and can impede the validity of the findings.

Theory/Rationale
The authors argue that hostility is a result of people trying to overcome inequality (be it political and/or economic). This should be more likely in poor and undemocratic countries. This makes political and/or economic inequality the proximal cause and level of democracy a distal cause. I do not understand how the authors can disentangle these interdependent relationships and formulate 3 hypotheses. These hypotheses cannot be independently tested since the variables are confounded. Can the authors clarify?

Thank you very much for asking us to clarify. We revised our theory / methods / and conclusions sections to clarify a number of important points:

1) Our goal was not quite to argue that “hostility is the result of people trying to overcome inequality”. Instead, we clarify our view in the revised introduction as such (following a novel and detailed paragraph on prior research on status, inequalities and hostility:

   “On the basis of this research, we expect systematic variation across countries in their levels of online political hostility. Status-seeking individuals may be more active or numerous in politically and economically unequal countries (i.e., in countries that are more undemocratic and with greater income disparity), making all forms of interactions more hostile, including online discussions about societal matters. According to this account, psychological motivations for acquiring status would get activated or exacerbated to varying degrees by the levels of inequalities observed in one's broader environment, thereby generating across countries variable levels of hostile discussions about politics on the internet.” p2

2) Given that it is impossible to experimentally manipulate societal levels of political and economic inequality and virtually impossible to identify as-good-as-random shocks in inequalities in a cross-sectional setting, our empirical ambition is not to demonstrate causal relationships, but to describe cross-country differences. This is not a trivial task to

do well. Reassuringly, the importance of descriptive work, especially in the domain of digital media is increasingly well-recognized in the literature (see e.g. Munger et al 2021).

3) Despite the fact that we are not in a position to demonstrate causation, our effort goes well beyond extant research–too often focused on US and Western samples–and is an important first step in our understanding of online hostility and their plausible causal explanations.

4) We have amended our theory section with references to prior studies demonstrating that frustrations of status aspirations are a cause of violent behavior and political violence. We hope our revisions make it clear that we test pre-registered, descriptive implications building on these causal theory.

The theory section suggests that the paper is about the personal experience of hostility. However, given the specific measurement it becomes clear that the study cannot disentangle people's observations of hostility online and personal victimization. The self-report questions leave a lot of room for interpretation: "I saw content that ridiculed people like me", "I saw content that threatened or harassed someone like me". It is unclear whether people agreeing that this happened to them in the last 30 days were truly victims themselves.

Thanks for requesting this important clarification. In our revised manuscript, we clarify that we measure **group-based** political victimization (online), not personal victimization. We remain convinced that using this group-based, self-reported measure is an important strength and not a weakness of our design. We updated the description of the measure in the main text:

> "our measure of online victimhood reflects the unique role group-based experiences play online. Prior research found that the bad reputation of online political discussions can be attributed primarily not to negative personal experiences, but to the elevated exposure to indirect hostility targeted at groups or individuals the person feels close to \cite{bor2022psychology}" p5

And also in the Methods section:

> "Note that we use the phrase ``[people] \textit{like me}" to specify the target of hostility. A viable measure of victimhood must take into account encounters with hostile content targeting individuals with whom the respondent identifies (because they identify with the same gender or political party, for instance). At the same time, most offensive content people encounter online does not target them personally \cite{bor2022psychology}." p20

We conducted Follow-up Study 1 to assess how victimization scores differ based on the target: personal victimization, group-based victimization, and general observations of hostility online. Our findings indicate that group-based victimization, as measured in our study, is distinct from both personally experiencing hostility (personal victimization) and merely observing hateful content online. This distinction supports the validity of our approach in capturing group-based

victimization as a separate phenomenon. We included a subsection on our follow-up studies in the methods section of the manuscript. Specifically, we write:

> "Follow-up Study 1 (N = 1,296) investigated whether our measure of victimhood varies depending on the target of hostility -- whether it is directed at the respondent personally, their ingroup, or anyone online. Our findings indicate that the measure specifically captures group-based victimization, distinguishing it from both general observations of hostility and direct personal victimization online." p22

We also note that we find similarly strong correlations between our pre-registered macro variables (lack of democracy, economic inequality) and country-level average hostility using our hostility-as-perpetrator measure, which is narrowly focused on hostile acts perpetrated by the respondent (see Online Appendix C). Accordingly, we can rule out the possibility that our findings are an artefact of the group-based victimization measure.

> The validation of this measure at the country level does not help very much since there may be alternative explanations for the prevalence of hostility. There are several alternative explanations that can interfere or invalidate the conclusions that the authors draw from their findings. For instance, differences in findings may be also due to the fact that moderation practices on platforms is more professional in richer/more democratic countries compared to poorer/undemocratic countries. This would result in higher prevalence of online hostility regardless of status seeking motivations or level of inequality. It is well known that platforms do a better moderation job in some countries versus others.

We concur with the reviewer that our empirical tests cannot unambiguously establish a causal link between macro-social variables and victimization. We have carefully revised our manuscript to avoid giving the wrong impression that we establish (or sought to establish) such a causal relationship. Instead, we clarify that our contribution is to map hitherto uncharted cross-cultural differences in average online hostility, relying on well-established psychological theories and confirming pre-registered descriptive hypotheses.

We also agree with the reviewer that moderation practices may contribute to cross-country differences in hostility. However, we view moderation practices as a plausible causal *mechanism* contributing to the observed hostility differences between equal and unequal countries. As such, we view this theory as complementary, rather than contradictory, to our main narrative emphasizing status-seeking motivations and political and economic inequalities.

To provide some insight, we used V-Dem data to examine the relationship between levels of liberal democracy, inequality and moderation. We rely on an expert coded indicator called "defamation protection" defined as the degree to which a country's legal framework provides protection against defamatory online content or hate speech. We find a positive correlation between liberal democracy and defamation protection (r = 0.32, p = 0.09), and a negative correlation between inequality and defamation protection (r = -0.47, p = 0.01) which suggests that democracies tend to have stronger legal frameworks for addressing harmful content, while

more economically unequal countries have less legal frameworks for addressing harmful content. Importantly, we believe it is much more plausible that democracy (economic inequality) drives better (worse) content moderation practices, rather than the reverse (i.e., that better moderation increases (reduces) democratic systems (economic inequality)). Going a bit further, we find that controlling for defamation protection reduce the association between both liberal democracy (by 18%) and inequality (by 52%), see details in Table 1 below. These analyses are consistent with – but should not be taken as causal evidence for – the argument that defamation protection and moderation practices mediate the relationships between macro indicators and average online victimhood. We have included these analyses in Online Appendix E4.

Table E4: OLS Regression Coefficients Defamation Protection and Falling Victim To Online Hostility

	(1)	(2)	(3)	(4)
Liberal Democracy Index	-0.18	-0.15		
	[-0.25, -0.11]	[-0.22, -0.08]		
Inequality (Gini)			0.39	0.19
			[0.07, 0.71]	[-0.15, 0.52]
Defamation Protection		-0.16		-0.22
		[-0.29, -0.04]		[-0.39, -0.05]
Constant	0.39	0.49	0.16	0.38
	[0.35, 0.43]	[0.40, 0.57]	[0.04, 0.27]	[0.18, 0.59]
Num.Obs.	30	30	30	30
R2	0.508	0.613	0.186	0.349
R2 Adj.	0.491	0.584	0.156	0.300

Note: 95% CI in parentheses

> Methods: The authors argue that the survey is representative of the national OR online populations. I am wondering how the authors can know the online population of a given country. Do the authors compare demographics with census data to show that samples are representative in terms of selected traits? Is the sampling comparable across countries? I doubt it since internet penetration rates differ considerably. This makes the comparison of samples across countries a complicated issue.

We clarified that our samples were designed to be broadly representative of social media users in each country. To do this, we relied on best practices from our survey provider, YouGov. In

countries with near universal internet penetration, YouGov quota samples benchmarking to the population demographic margins as provided by the national statistical bureau. In a few countries, where internet penetration is lower AND is skewed compared to the general population, YouGov relies on the demographic margins of the internet-using population – again based on the estimates of the national statistical bureau. Additionally, we screened out individuals who indicated no experience with using large online platforms (including instant messaging). We believe our procedure is by far the best to acquire comparable cross-country data, and the data quality and representativity of our data are on par or higher quality than most recent papers published in top outlets that often have to rely on convenience samples to overcome the difficulties of large cross-cultural studies (e.g. Arechar et al 2023 NHB, Imhoff et al 2023 NHB, Van Bavel et al Nat Comm).

We also conducted an empirical validation to assess the quality of our sample. We report the following in Online Appendix A:

> “Finally, Figure A5 benchmarks our data using our estimated popularity of Facebook against external data from the Meta Ad Manage, accessed via https://datareportal.com. Note that calculating the number of Facebook users per country is not an easy task, as discussed in detailed on DataReportal’s Methods page, https://datareportal.com/notes-on-data. Hence these numbers should be taken with a grain of salt. Furthermore, even apart from the noisiness of the FB benchmark, the absolute levels between the benchmark and our data are not comparable, because national FB user numbers are calculated as a proportion of eligible population (above the age of 14). Meanwhile, our samples include only adults (18+) and only people who use at least one social media platform. Nonetheless, apart from a few countries (e.g. Pakistan), the countries where Meta reports Facebook to be more popular also include more Facebook users in our sample.”

As we note in the manuscript, the correlation between these two measures is strong to begin with ($r = 0.48$). If we drop our worst outlier, Pakistan, it increases even further to $r = 0.58$. We take this as notable empirical support for the quality of our data.

Figure A.5: The popularity of Facebook according to the Meta Ad Manager and our samples. Pearson's r = 0.48 (95%CI: 0.14-0.72).

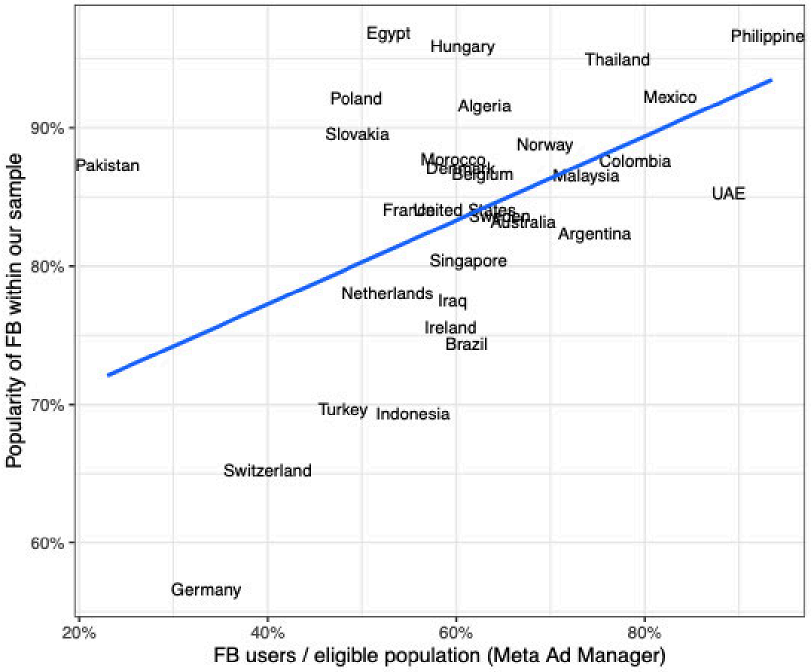

Note: We access data on Facebook user base via https://datareportal.com/ relying on their 2023 country reports (x-axis). We calculate the proportion of respondents in each country sample who says they use Facebook at least occasionally (y-axis).

How do the authors justify the sample size of 500 per country? Is that based on a power analysis?

We discuss sample size justification in our pre-registration:

> "Our primary constraint is money. That said, based on a single country pilot study, with N = 500, we found that the standard error of standardized beta coefficients for the association between online and offline hostility (H1), and online hostility and status-driven risk taking (H2) are around 0.02 and 0.04, respectively. This means we have 80% power to find support for our prediction as long as the true association with offline hostility is above 0.5 + 2.44 * 0.02 = 0.55 in a country; and above 0.1 + 2.44 * 0.04 = 0.20 for status driven risk taking and meaninglessness. When it comes to country means (e.g. estimating prevalence of victimhood) the 89% credible intervals should span 1/sqrt(500) * +/- 2* 1.6 = +/- 0.14 SD-s. Should we want to directly compare hostility across two countries, we would have 80% power using a 89% credible interval to detect a mean difference of 2/sqrt(500) * 2.44 = 0.22 SD. We base our sample size calculations on using 80% power and 89% credible intervals: Howard S. Bloom. Minimum Detectable Effects: A Simple Way to Report the Statistical Power of Experimental Designs. Evaluation Review, 19(5):547–556, 1995."

We added an explicit reference to this power analysis to the Methods section.

> The authors relied on self-reported measures of hostility and argued that this was a valid measure. They refer to a study in which it is stated that "Beliefs about what hostile speech is likely to fluctuate across individuals and national contexts".

We acknowledge that the manuscript was not clear enough about this important issue. In the revised manuscript, we make a crucial distinction between objective hostility and subjective victimization. "Objective hostility" refers to the ambition to identify speech acts which are unacceptable by some (near) universal moral standard at least within a given community. Policy makers, judges, platform moderators must decide whether a given speech act is offensive enough to warrant some action (censorship, bans, fines, etc). We argue that attempting to find moral standards applicable to 30 countries is impossible except for the most severe cases (death threats, etc). In short, we are in full agreement with the quoted sentence.

For this reason, we thought that it is most fruitful – and indeed, the only feasible comparative approach – to focus on "subjective victimization" instead. Here, we judge speech acts not against some universal and objective moral standard, but against receivers' subjective experience. If a respondent claims that they were offended by something, we consider it as a normatively important and empirically valid perception. Basically, we see people as the competent and ultimate judges of their experiences. Our measure hence overcomes the issue that the exact same sentence may be offensive to some people, but not others (especially across countries).

Admittedly, self-reported measures have their weaknesses. We sought to mitigate the weakness intrinsic in self-report measures by using best practices e.g. by offering respondents detailed definitions to avoid ambiguities and by asking about a specific time window to reduce memory biases. We address the issue of demand effects in followup study 2, by examining the extent to which our measure of hostility is potentially biased by respondents under-reporting hostile behaviors out of social desirability concerns (we use a virtual dice rolling method to do so). Our results show no evidence of social desirability bias in our measure of victimhood or hostility perpetration. The forced-response design allowed respondents to answer honestly without fear of judgment, as any response could be attributed to a dice roll. If social desirability bias was present, we would expect higher estimates of victimization and lower estimates of hostility perpetration in the dice-roll condition compared to direct self-reports, but no such differences emerged. The estimated prevalence of both victimhood and hostility remained unchanged. We included a subsection on our follow-up studies in the methods section of the manuscript. Specifically, we write:

> "Follow-up Study 2 (N = 849) examined whether respondents might under-report hostile behaviors due to social desirability concerns. To assess this potential bias, we implemented a virtual dice-rolling game --- a method specifically designed to reduce social desirability in self-reports \cite{BlairDice}. Our results reveal no evidence of social desirability bias in measures of victimhood or hostility perpetration." pp 22-23

I would also assume that users in countries with a higher exposure to online and offline hostility become desensitized and respond differently to self-report measures of hostility. Thus, self-report measures would not be a valid indicator in the present study.

We thank the reviewer for this suggestion. First, we would like to note that if desensitization substantially affected our victimization estimates, it would suppress the cross-country differences we find, thus making our hypothesis tests too conservative. Put differently, bias due to desensitization would lead us to underestimate country differences, because people in hostile countries would be numb to the hostility, whereas people in peaceful countries would "overreact". As such, desensitization does not fundamentally threaten the validity of our conclusions.

Nonetheless, as an additional test of the validity of our measures, we conducted Follow-up Study 3 to empirically examine the potential impact of desensitization. Specifically, employing an establish experimental framework for hostility desensitization (Soral et al 2018), we investigated whether repeated exposure to online hostility might lead respondents to downplay their own victimization, thereby biasing our measure of hostility victimization. To test this, we exposed participants to a series of hostile messages and assessed their self-reported victimhood and perpetration of hostility. Our results show no evidence that repeated exposure reduces perceptions of victimhood. Similarly, exposure does not make individuals significantly more or less likely to report engaging in hostile behavior themselves. We included a subsection on our follow-up studies in the methods section of the manuscript. Specifically, we write:

> "Follow-up Study 3 (N = 864) explored whether repeated exposure to online hostility desensitizes respondents, potentially biasing victimhood estimates. Following an established procedure to gauge the effects of desensitization to hostility \cite{soral2018exposure}, participants were exposed to a series of hostile messages, after which we assessed their perceived victimhood. We find no evidence that repeated exposure diminishes perceptions of victimhood, nor does it significantly influence individuals' likelihood of reporting that they engage in hostile behavior themselves." p23

Another, yet related issue, is the invariance of measurement that needs to be demonstrated, e.g., by means of confirmatory factor analysis. If measurements are not invariant, comparisons across populations become problematic because differences in means or correlations may be due to differences in measurement and do not represent meaningful differences or correlations. Differences in reliabilities in the methods section indicate a lack of measurement invariance that should be tested using formal tests.

First of all, we are grateful for the nudge to pay more attention to measurement. We conducted formal tests of measurement invariance closely following the methodology employed in a recently published Nature Human Behavior paper by Imhoff and colleagues (2023). We find that our online and offline hostility measures are consistent with scalar invariance assumption,

whereas victimhood attains partial scalar invariance. Status-driven risk taking attains metric invariance. Overall, these results (as well as the pre-registered theoretically predicted macro-correlations) strongly imply that our results reflect meaningful differences and similarities across countries. We report details in Online Appendix L.

> It is unclear what the use of the "novel, seven-item survey measure to capture the three dominant narratives of social media's impact on politics" should be. There is no theory that explains the role of this variable and how it is related to perception of hostility.

The three dominant narratives are derived from the theoretical framework outlined by Tucker et al. (2017). We have clarified this in the manuscript. Our goal in looking at these narratives was not to directly examine the correlation between these narratives and political hostility, but rather to explore how people from different countries broadly perceive the impact of social media on society and how this relates to liberal democracy and economic inequality levels. This line of inquiry complements our analysis of victimhood and hostility to investigate public views on the societal effects of social media. We therefore did not have explicit expectations about how these narratives might relate to hostility a priori.

We have clarified this: (see pp. 12-13)

> "So far, our investigation has focused on \textit{political hostility} as a key aspect of citizens' experiences with social media. We showed that online hostility is worse in undemocratic and unequal countries and that it strongly correlates with offline hostility and status-driven risk-taking. Given the widespread concerns about the impact of social media, including in highly democratic countries \cite{haidt2022after}, a natural next step is to ask how people across societies perceive the broader role of social media: Do they see it primarily as a negative force that exacerbates societal turmoil, or do they also view it as offering political opportunities that might outweigh its downsides?
>
> To explore this, we assess endorsement of three dominant narratives in public and scholarly discourse about the political effects of social media, as outlined by \cite{tucker2017liberation}. These narratives offer a structured framework for understanding how different societies conceptualize the role of social media in political life. The \textit{turmoil} narrative sees social media as amplifying extremism and misinformation, distorting public opinion, and undermining democracy. The \textit{liberation} narrative emphasizes their role in democratizing discourse and fostering political engagement. Finally, the \textit{oppression} narrative highlights how illiberal states exploit them for manipulation and repression. To quantify public endorsement of these narratives, we developed a novel seven-item survey measure based on Tucker et al.'s framework, allowing us to assess how these perceptions relate to levels of democracy and inequality."

> Results: Can the authors say anything about the size of the effects? What does a regression coefficient between .025 and .05 mean? When the authors look at platform

differences their interpretation is that there are only small differences. Transferred to the regression coefficients: are these effects meaningful?

We revised our manuscript with an eye to give more meaningful interpretations of our effect sizes. The interpretation depends on the specific model. For example, in the cross-country analysis, we interpret a coefficient of 0.05 in this way: “Countries that are one standard deviation more democratic experience 5 percentage points less online hostility (89\%CI [-0.07, -0.04]), which constitutes a remarkable 71\% of a standard deviation in cross-country victimhood.” p7 However, a similar coefficient denotes a small difference when it comes to individual variation in victimhood, e.g. on platform coefficients: “Even the largest coefficient -- for Twitter -- shows only a difference of 0.05 (or 5 percentage points), amounting to 16\% of a standard deviation in respondent-level victimhood.” see Online Appendix D.

Findings related to anomia and polarization: The authors demonstrate that anomie and polarization are not related to the experience of online hostility. Although this is an important finding, the more important result would be to formally test whether these effects are different from the effects that were obtained. Thus, do the effects that were obtained are different from the non-significant results on anomia and polarization.

To demonstrate that indeed the correlation between status-driven risk taking (SDRT) are stronger than those with anomia and polarization, we amend our analyses in two ways. First, we display the country-level SDRT correlations as a benchmark for the null result for anomia and polarization. The large gap between the confidence intervals conveys clearly that there is no uncertainty in these models about the difference of these two estimates.

As a more formal test, we also employ Leave-one-out cross-validation to compare the predictive power of the model including SDRT and those that include anomia and polarization, respectively. In both cases, we find that the models with SDRT fit the data much better and do a better job at predicting hostility levels (for anomie, the model comparison yields an ELPD (SE) of -850.2 (69.9), and for polarization, an ELPD (SE) of -728.9 (64.4)).

Figure F.1: Feelings of meaninglessness are consistently unrelated to being hostile in online political conversations. The figure displays the country-level posterior distribution of standardized beta estimates. The vertical line at 0.1 denotes the effect size we preregistered to test our hypothesis against. We juxtapose meaninglessness estimates to identical estimates for status-driven risk taking (SDRT), highlighting the substantial difference between the two.

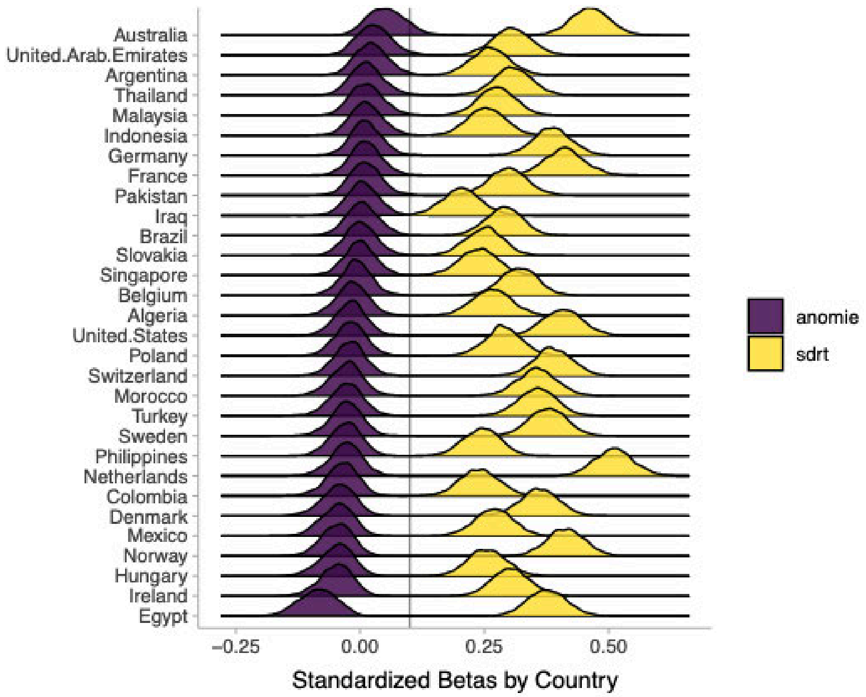

Note: Estimates are based on Bayesian multilevel regressions with weakly informative priors.

Reviewer #2:

Remarks to the Author:
In their manuscript "Undemocratic and unequal countries experience more political hostility on social media – Evidence from 30 Countries" the authors describe the results of a large survey about self-reported experiences with political hostility online and also offline.
The work makes an interesting contribution to an important topic and the main value is the global perspective that is provided here. I think some conclusions from the data are warranted and important, most interestingly the interaction between individual factors, such as offline hostility or status seeking, and the country-level factors. Those results potentially contribute to the picture of social media making certain groups particularly visible, namely the unsatisfied with the system. Those groups might be smaller in more democratic countries and broader in less democratic countries, and thereby either causing more turmoil in the "functioning" systems and having a very similar effect in less

"functioning" systems, but where the turmoil is justified and rather materialized and seen as liberation.

We are grateful for the praise.

Other conclusions are less justified by the data in my view, namely that social media platforms and algorithms play a little role in those behaviours, because the survey is neither specific in the type of platforms that are used nor how platforms co-evolve with their users, which can lead to different outcomes in different countries but not excluding the possibility that the same mechanisms are at play (see above, making the dissatisfied be more visible in a system, for example).

We acknowledge that the previous version of our manuscript has made too strong claims on the role of algorithms and platforms. We made several revisions to the manuscript to clarify that our work should be seen as an important complement to extant work on design features. The following new paragraph concluding our introduction offers a good summary:

"To sum up, these findings do not challenge the claim that the design features of social media may have exacerbated online political hostility. Instead, they provide strong evidence that online political hostility cannot be understood in isolation from the larger socioeconomic and political context of societies. In addition, our results suggest that social media may empower groups of people who are strongly dissatisfied with their standing within society. In democratic and equal countries, such empowerment may be viewed as disruptive; in more autocratic and less equal countries, it may allow some degree of liberation for some segments of society." pp 3-4

And in the General Discussion:

"[O]ur findings offer an important corrective to conventional wisdom about the origins of hostility in online discussions on societal matters. When proposing explanations of the hostile tone of social media interactions, pundits, policy makers and researchers often blame anonymity, algorithms, and other features of social media platforms. Our research did \textit{not} seek to test the role of these technological affordances. Our findings, however, clearly show that online hostility is not just an accident or an artifact of well-meaning individuals' corruption by technology. First, the sociopolitical context matters: undemocratic and unequal countries suffer more from online hostility. Second, our data show that personality dispositions are robust correlates of online hostility: in every country we surveyed, people who are hostile online are also hostile in face-to-face political discussions, and they score substantially higher on dispositions for aggression in the form of status-driven risk taking. P15

The other conclusion, that shines through the manuscript and is not supported by the survey data, in my view, is the causal direction of people being status seeking or hostile offline leading them to be hostile online, the mechanism could also work the other way,

i.e., people radicalizing online and carrying this hostility offline, cross-sectional data just cannot distinguish that.

We acknowledge this limitation in our revised conclusions: our empirical approach can establish only correlations and not causal relationships. We acknowledge that frustrations from hostile online interactions can spill over to offline relationships, although we are more skeptical of the idea that hostile online interactions could suffice to shape a core psychological trait like status seeking motivations. Specifically, we added the following qualification to the section on the psychological roots of online hostility:

> "While our data cannot establish causality, theory and prior research suggest that status-driven risk-taking, as a stable trait, is more likely to drive online hostility than be shaped by it." p9

> Lastly, the self-reported measures may have an advantage of comparability due to language-barriers of e.g., automated toxicity detection across languages, but it also comes with a downside that self-reporting on online behavior is not very reliable (see https://www.nature.com/articles/s41562-021-01117-5).

We are grateful for being pointed to this interesting research on the relationship between self-reported and objective measures of social media use. We reference this research in our updated manuscript. That said, our takeaway from the article is not that "self-reporting on online behavior is not very reliable". Parry et al (2021) focus on social media use, which is a habitual, often compulsive, emotionally neutral behavior. By contrast, engaging in hostile political discussions is rare and emotionally vivid. Indeed, a study comparing self-reported incivility with behavioral data concluded that "those who self-disclose uncivil online behavior also tend to generate content on social media that is uncivil" (Hopp et al 2020). Similarly, in the domain of political news sharing — another rare and often emotional form of online political behavior — self-report measures correlate fairly strongly with actual sharing decisions on social media (Mosleh et al 2020).

Still, it is plausible that there is some bias in our measures. However, even if self-reports are not fully accurate, our hypotheses concerning cross-country and individual-level differences would remain valid unless these biases systematically correlate with the very factors we analyze—such as status-seeking or cultural norms of expressing hostility. While we cannot test these alternative explanations, we see no strong theoretical arguments for their presence.

We have run Follow-up study 2 to examine the extent to which our measure of hostility perpetration was potentially biased by respondents under-reporting hostile behaviors out of social desirability concerns. Using a virtual dice-rolling game that is specifically designed to reduce social desirability effects, we find no evidence of social desirability bias in our measures of victimhood or hostility perpetration. We included a subsection on our follow-up studies in the methods section of the manuscript. Specifically, we write:

"Follow-up Study 2 (N = 849) examined whether respondents might under-report hostile behaviors due to social desirability concerns. To assess this potential bias, we implemented a virtual dice-rolling game --- a method specifically designed to reduce social desirability in self-reports \cite{BlairDice}. Our results reveal no evidence of social desirability bias in measures of victimhood or hostility perpetration." pp 22-23

This could potentially be compensated by backing the hostility prevalence data with some automated measure of hostility from online text (e.g., comment sections), in addition.

As we explained above in our response to the Editor, we carefully examined the question of using behavioral measures of hostility and ran original analyses on the Twitter Geographic Sentiment Index. We have arrived at the conclusion that this endeavour would likely not bring about the expected benefits. See our detailed discussion in pp 3-4 of this Memo.

Those are my main concerns that need to be resolved in my view, so to make this a contribution that adds to our understanding of the global effects of social media use (which is mostly the aspect of empowering the unsatisfied, that can be a liberating or destabilizing thing, depending on the system). I would be interested to hear from the authors about this and to read a revised version of the manuscript that makes the scope of the contribution clearer.

Thanks again for your constructive and thoughtful comments.

a few more specific questions:
- is there data on the platforms that people mostly use in the respective countries?

Yes, we report data on this in Figure A.2. (reproduced below)

Figure A.2: Distribution of social media platform use across countries. Lighter colors denote a higher share of society using a given platform.

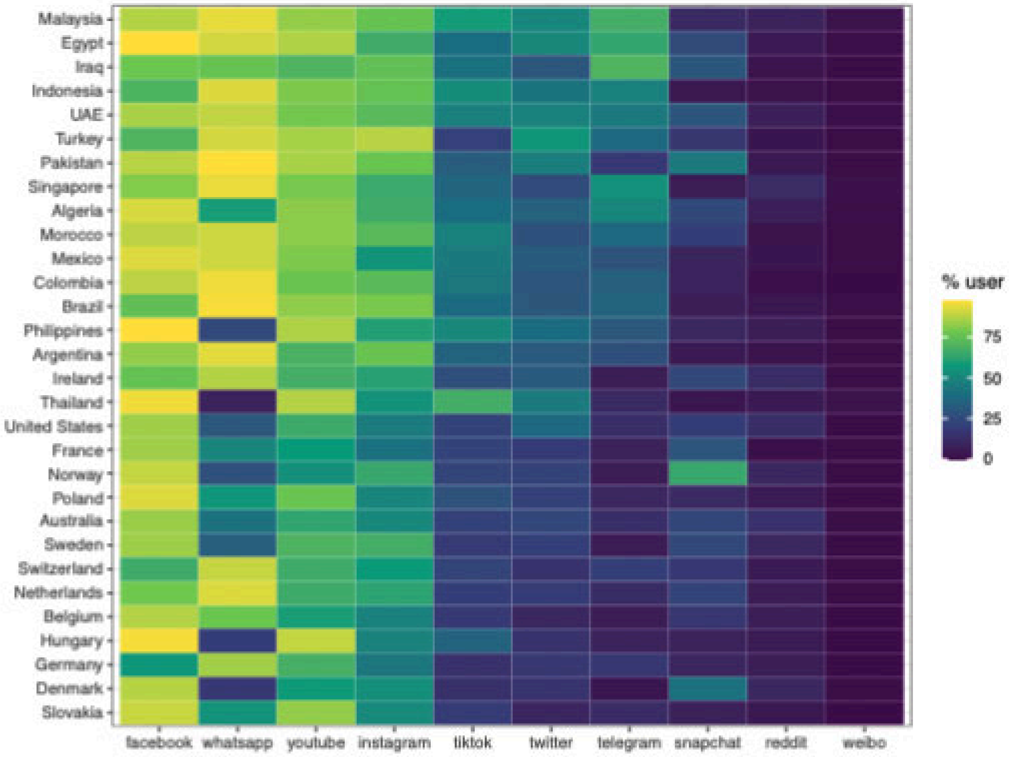

Note: Our questionnaire asked respondents "What type of social media accounts do you use?" Respondents could choose as many platforms as they wished. We employed survey weights in calculating the proportions in the figures.

- are there other country-level attributes that can be used to describe online victimhood?

Yes, we report these in Online Appendix E. Our results show that :

1. Victimhood tracks most forms of country-level measures of violence and hostility (e.g. political violence, hate speech);
2. Correlates negatively with each measure of democracy (electoral, participatory, deliberative, egalitarian)
3. Correlates positively with several measures of social inequality (e.g. equality of power distribution, access to public services, access to state jobs and businesses).

- the problems of self-reported hostility are probably affected by demand characteristics, correct?

We take the issue of demand effects very seriously.

1. We conducted followup study 2 to directly test this issue. As described in detail above (pp 11-12 of this memo), we find no evidence that demand characteristics bias our results.
2. These results dovetail with our prior empirical investigations which we sum up in the manuscript like this:

   “We address concerns about presumed differences in normatively permissible behavior online versus offline by estimating the correlation of hostile behavior across discussion environments for each of the five items in our index. Our logic is that even if such norm asymmetries existed (although Ref (7) finds no evidence), they would likely concern milder forms of hostility. People may disagree about where the line is when it comes to ridicule offline versus online, but it is unlikely that the same ambiguity exists about harassment for example. Results in OA Section N demonstrate that the correlation between offline and online hostility is equally strong across all forms of hostility” p24.

Reviewer #3:

Remarks to the Author:
This study uses self-reported survey data from 30 countries to examine sociopolitical context and factors that may play a role in the developing hostility – thus going against the social science (and public discourse) that blames much of recent sociopolitical hostility on social media platforms. It is a much-welcome intervention into this space, which has been so dominated by a convenient but not empirically supported boogeyman. The authors argue that online hostility may be driven by a small set of status-seeking individuals, who may be just as likely to engage in offline hostility, and who may be particularly apt to surface in contexts that suffer democratic threats, including inequality. Across this well-done study, the authors provide a strong argument for how we should think of the role of social media in fomenting our political ills – and a clarion call for including all-important sociopolitical context when analyzing those relationships. Political context – and culture – matter.

Thank you!

A note: this study finds that a likely small set of status-seeking individuals may be responsible for driving online hostility – see also the work on repeat offenders, the “dirty dozen”, elite disinformation, below:
https://252f2edd-1c8b-49f5-9bb2-cb57bb47e4ba.filesusr.com/ugd/f4d9b9_b7cedc0553604720b7137f8663366ee5.pdf
https://www.cip.uw.edu/2023/10/20/new-elites-twitter-x-most-influential-accounts-hamas-israel/
Tripodi, F. B. (2022). The propagandists' playbook: How conservative elites manipulate search and threaten democracy. Yale University Press.

We are grateful for these references, which we now cite in our paper “Social media can, in turn, amplify the voice of a few highly motivated individuals. Notably, a large chunk of political and medical misinformation in the US originate in a handful of users \cite{DisinformationDozen, Osmundsen et al , Tripodi 2022}.” p2

> The manuscript states, “Finally, we find that people in more undemocratic countries, despite more experiences with hostile online debates, are more likely to emphasize the liberating potential of social media.” This aligns with a recent Knight & Gallup study that found Black Americans were more likely to engage in offline political activities because of online political discussions, despite being endemically marginalized from democratic processes within the country (US).
> https://knightfoundation.org/reports/media-and-democracy/

Excellent point. We incorporated this reference on the liberating potential of social media and cited it in the General discussion.

> The manuscript states, “Importantly, the returns to individuals who are motivated to dominate are particularly high in sociocultural contexts that are characterized by various forms of inequality.” I hope the US is considered in this case – AND its uniqueness, thought similar to other western democratic countries, of the hostility coming from those who do actually possess power – often by upholding systems of oppression – but nonetheless *feel* they are the ones impacted by inequalities.

Thank you for the very interesting remark. We could partially address this question with the same data we use for our novel MRP analyses (N = 4200 quota sampled online respondents via Lucid). Using the same self-reported online hostility outcome we utilize in the present project, and standard demographic categories, we find that younger respondents, men, respondents with lower educational attainments, as well as rational minorities report significantly and substantially more political hostility than older, female, highly educated and white or Asian respondents. While we find these results very interesting, to comply with space limitations and given the cross-cultural focus of the present paper, we have not added them to the manuscript. If the Editors or Reviewer find the results important, we are happy to add them to the Online Appendix.

	Dependent variable:
	Online hostility
Age: 30-44	-0.11* (0.04)
45-64	-0.43*** (0.04)
65+	-0.60*** (0.05)
Female	-0.20*** (0.03)
Education: High School	-0.35*** (0.08)
Some college	-0.51*** (0.08)
College	-0.47*** (0.08)
Post-grad	-0.48*** (0.09)
Ethnicity: Black	0.23*** (0.05)
Hispanic	0.10* (0.05)
Constant	0.77*** (0.08)
Observations	4,227
Adjusted R^2	0.09
Residual Std. Error	0.95 (df = 4216)
Note:	*p<0.05; **p<0.01; ***p<0.001

We also call attention to recent work on the Need for Chaos, (Petersen et al 2023). Test 6 (pp 1500-1502) offers a much more detailed and nuanced analysis of these questions in the US context than what our present data allow.

> How do the authors feel about the measure of victimhood not accurately reflecting true targets of hostility? As in, for example, a cis straight white man feels a gay-pride post is hostile. Normatively, there is no victim here – the cis straight white male still holds exceptionally more power than any queer person.

We acknowledge that our measure of victimhood does not distinguish between normatively justified and unjustified perceptions of victimhood. This is indeed a limitation of our approach. However, any attempt to determine when feeling victimized is 'true' or 'false' would require engaging in complex normative judgments about power, legitimacy, and perceived harm—an important but vast and contentious debate. Given our study's scope, we prioritize capturing subjective experiences as they shape political behavior, while recognizing that these perceptions do not always align with structural inequalities. We added the following acknowledgement to our discussion of the measure: “However, a potential limitation of our measure is that it may include perceptions of hostility that are not universally recognized as harm, such as individuals from majority groups feeling targeted by expressions of minority identity or advocacy.”

> Of figure 1 – can you note that the scale and key, I assume, is for reported victimhood of online political hostility?

We are grateful for this good suggestion which we implemented.

> On polarization, the manuscript states, “We sought to benchmark the role of status-driven risk taking and meaninglessness to a popular psychological predictor, namely affective polarization. Surprisingly, we found no evidence for a relationship between polarization and political hostility either.” Perhaps worth incorporating this recent critique of polarization work to contextualize this finding: Kreiss, D., & McGregor, S. C. (2024). A review and provocation: On polarization and platforms. New Media & Society, 26(1), 556-579.

Thanks, we amended the sentence to incorporate Kreiss and McGregor’s important work: “To our surprise, but consistently with some recent critique of the polarization literature (Kreiss & McGregor 2024), we found no evidence for a relationship between polarization and political hostility either”.

> The study includes this fascinating finding: “Individuals high in status-driven risk taking are more hostile if they live in a more democratic or – to a lesser extent – in an unequal country.” There are many possible interpretations for this, but one likely one is because the status-seeking individuals would rather live in a less democratic country where they can take advantage of their relatively high status within an unequal society.

This is a plausible interpretation. Alas, our data does not allow us to explore it empirically.

> The manuscript states, “In democracies and economically equal countries, online hostility is, in contrast, more exclusively driven by the usual suspects of aggression: young male status seekers.” Might be worth trying to find some of the work that examines their particular role in social media (e.g., the manosphere, trolling, incels, etc.)

This is a good point, we added citations to the following two papers studying the online manosphere at the end of this paragraph:

> “In democracies and economically equal countries, online hostility is, in contrast, more exclusively driven by the usual suspects of aggression: young male status seekers (Gotell & Dutton 2016, Horta Ribeiro et al 2021).” p12

> I’d suggest giving an example or two of the “usual suspects” references here: “In undemocratic or unequal countries – even if they suffer more from online hostility overall – the same connectivity may liberate a broader public disenfranchised from their society, as we found engagement in hostility beyond the ‘usual suspects’ in these countries.”

We have clarified the problematic sentence: “ In undemocratic or unequal countries, connectivity plays a different role. Although people are exposed to more online hostility overall, this same connectivity allows a broader and more diverse public—spanning various ages and genders—to

engage in political discourse. We found that hostility in these contexts was not limited to young males but extended across a wider demographic." (p16)

> Just wanted to give a huge amen to this, in the manuscript: "Decision makers' current efforts to regulate tech giants can have beneficial effects, but they are unlikely to resolve the social tensions reflected on social media. Instead, politicians need to "fix society": to face the possibility that to truly improve the climate of online political discussions, it will be necessary to make societies more democratic, more equal, and more harmonious offline."

Thank you!

> Were the sample medians (e.g., for age), calculated per country or across the whole multi-country sample?

We now clarify that it's per country.

References:

Arechar, A. A., Allen, J., Berinsky, A. J., Cole, R., Epstein, Z., Garimella, K., ... & Rand, D. G. (2023). Understanding and combatting misinformation across 16 countries on six continents. *Nature Human Behaviour*, *7*(9), 1502-1513.

Bartram, D. (2013). Scalar equivalence of OPQ32: Big Five profiles of 31 countries. *Journal of Cross-Cultural Psychology*, *44*(1), 61-83.

Chan, D. (2010). So why ask me? Are self-report data really that bad?. In *Statistical and methodological myths and urban legends* (pp. 329-356). Routledge.

Cinelli, C., & Hazlett, C. (2020). Making sense of sensitivity: Extending omitted variable bias. *Journal of the Royal Statistical Society Series B: Statistical Methodology*, *82*(1), 39-67.

Dong, Y., & Dumas, D. (2020). Are personality measures valid for different populations? A systematic review of measurement invariance across cultures, gender, and age. *Personality and individual differences*, *160*, 109956.

Grinberg, N., Joseph, K., Friedland, L., Swire-Thompson, B., & Lazer, D. (2019). Fake news on Twitter during the 2016 US presidential election. *Science*, *363*(6425), 374-378.

Gotell, L. and Dutton, E. (2016) "Sexual Violence in the 'Manosphere': Antifeminist Men's Rights Discourses on Rape", International Journal for Crime, Justice and Social Democracy, 5(2), pp. 65-80. doi: 10.5204/ijcjsd.v5i2.310.

Guess, A., Nagler, J., & Tucker, J. (2019). Less than you think: Prevalence and predictors of fake news dissemination on Facebook. Science advances, 5(1), eaau4586.

Hopp, T., Vargo, C. J., Dixon, L., & Thain, N. (2020). Correlating self-report and trace data measures of incivility: A proof of concept. *Social Science Computer Review*, *38*(5), 584-599.

Horta Ribeiro, M., Blackburn, J., Bradlyn, B., De Cristofaro, E., Stringhini, G., Long, S., Greenberg, S., & Zannettou, S. (2021). The Evolution of the Manosphere across the Web. Proceedings of the International AAAI Conference on Web and Social Media, 15(1), 196-207.

Imhoff, R., Zimmer, F., Klein, O., António, J. H., Babinska, M., Bangerter, A., ... & Van Prooijen, J. W. (2022). Conspiracy mentality and political orientation across 26 countries. *Nature human behaviour*, *6*(3), 392-403.

Kreiss, D., & McGregor, S. C. (2024). A review and provocation: On polarization and platforms. New Media & Society, 26(1), 556-579.

Luo, J., Lyu, H., Luo, H., Tang, Z., Kong, D., \& Wen, X. (2023). Global sentiment tracking on Twitter during the COVID-19 pandemic. \textit{Scientific Data, 10}, 744.

Mosleh, Mohsen & Pennycook, Gordon. (2020). Self-reported willingness to share political news articles in online surveys correlates with actual sharing on Twitter. PLOS ONE. 15. e0228882. 10.1371/journal.pone.0228882.

Munger, K., Guess, A. M., & Hargittai, E. (2021). Quantitative description of digital media: A modest proposal to disrupt academic publishing. *Journal of Quantitative Description*, (1), 1-13.

Parry, D. A., Davidson, B. I., Sewall, C. J., Fisher, J. T., Mieczkowski, H., & Quintana, D. S. (2021). A systematic review and meta-analysis of discrepancies between logged and self-reported digital media use. *Nature Human Behaviour*, *5*(11), 1535-1547.

Petersen, M. B., Osmundsen, M., & Arceneaux, K. (2023). The “need for chaos” and motivations to share hostile political rumors. *American Political Science Review*, *117*(4), 1486-1505.

Soral, W., Bilewicz, M., & Winiewski, M. (2018). Exposure to hate speech increases prejudice through desensitization. Aggressive behavior, 44(2), 136-146.

Tripodi, F. B. (2022). The propagandists' playbook: How conservative elites manipulate search and threaten democracy. Yale University Press.

Van Bavel, J.J., Cichocka, A., Capraro, V. et al. National identity predicts public health support during a global pandemic. Nat Commun 13, 517 (2022).

Welzel, C., Brunkert, L., Kruse, S., & Inglehart, R. F. (2023). Non-invariance? An overstated problem with misconceived causes. Sociological Methods & Research, 52(3), 1368-1400.

Yu, Z., Sen, I., Assenmacher, D., Samory, M., Fröhling, L., Dahn, C., ... & Wagner, C. (2024). The Unseen Targets of Hate: A Systematic Review of Hateful Communication Datasets. Social Science Computer Review, 08944393241258771.

Zhirkov, K., & Welzel, C. (2024). Rethinking Measurement Equivalence in Comparative Political Research. *Political Research Quarterly*, 10659129241241753.

REVIEWER COMMENTS:

Reviewer #1 (Remarks to the Author):

I appreciate the effort that the authors put into revising the manuscript. In this revision, they have addressed nearly all of my concerns in a very convincing way, either by providing additional empirical evidence to support their argument or by offering clarifications and explanations that enhance the understanding of their argument.

Thank you!

One final issue that remains pertains to the findings at the end of the paper. I still believe that the last part of the paper is not well integrated and lacks specificity. The discussion around perceptions of threats or narratives (liberation, turmoil, oppression) remains unclear, and the clarifications in the revised manuscript did not help alleviate my concerns. This issue becomes apparent when examining the hypotheses: the findings related to perceived online hostility are based on hypotheses that are sufficiently theorized, and all headings are affirmative in nature. The section on perceptions of the impact of social media begins with a question and lacks expectations regarding how specific contexts or predictors may influence such perceptions. The justification that a single paper provided a “structured framework” for formulating survey items is weak. It is unclear what these items truly measure and whether they form a valid and reliable scale. Developing a news scale requires more than just one inspiring paper. Methods books outline the extensive process required to validate a scale and establish construct validity, steps

that are absent from the present manuscript. For example, did the items survive a confirmatory factor analysis? Do the scales correlate as expected with constructs that should be related to these scales? Are these scales invariant across cultural contexts? I suggest removing this weaker section from the manuscript, as I believe the paper is stronger without it, but I defer that decision to the editor.

We have followed the Reviewer's suggestion and moved the section called "How do people around the world perceive the impact of social media on society?" to the Online Appendix (K).

Reviewer #2 (Remarks to the Author):

I thank the authors for their response and the revision of the manuscript. Unfortunately, I still see the existing concerns that I raised initially, which mostly boils down to claims and conclusions that are not fully supported by the presented data. I do find the dataset still very interesting and valuable and I agree with some of the conclusions, e.g., that the role of social media may be different depending on the social- and political context.

Thank you!

However, the present data does not allow for specific mechanisms like "Social media may thus have provided democratic audiences with a window into minority frustrations that hitherto went unnoticed." It could as well be that the effects of social media interact with country-level factors.

We deleted this sentence from the abstract, and revised it to include a more neutral interpretation of the empirical findings. We conclude the abstract with the statement "Overall, these findings highlight how online political hostility is intertwined with wider societal tensions."

If I read the manuscript correctly, the main message is that social media is merely a mirror of society and not a driver, which I don't see can be confidently argued with the data at hand. In my view the narrative of the paper needs to be thoroughly revised to report the descriptive data in a neutral way and discuss different potential mechanisms behind the observations in parallel. The current presentation of the manuscript makes it additionally difficult to understand the (slightly mechanistic) argumentation of the paper that is based on a combination of various cross-sectional descriptive results.

We have carefully revised the manuscript to give a more neutral interpretation of our descriptive data, eliminating discussion on what we perceive to be the common wisdom on social media having large effects on generating hostility and the admittedly speculative discussion of potential implications from the previous version. Furthermore, we have removed all mentions of social media being a "mirror" or "window" of society and added a new paragraph acknowledging that social media may also be a driver of offline hostility (see paragraph quoted above).

Let me explain in more detail:

For example, an argument I disagree with is stated in the introduction of the revised manuscript as "If online hostility were solely a product of the technological affordances of global social media platforms, one would expect similar levels of hostility across countries, with only minor random variation."
This is only true if one would assume purely homogeneous effects of social media and no interactions with individual factors, which is indeed a naive view that is not propagated in the scientific discussion.

In our revision, we removed the null hypothesis of no cross-country differences, and put more emphasis on the alternative view, where we are actually in complete agreement with the Reviewer: “social media platforms may also be intertwined with broader socioeconomic and political tensions, produced outside the platforms”.

Relatedly, the fact that online and offline hostility are correlated is not a proof for selection effects, those behaviours can influence each other in both ways. There is evidence for online hostility leading to offline hate crimes, for example.

We explicitly acknowledge the possibility of reverse causality in a new paragraph in the Discussion section discussing alternative mechanisms (see paragraph quoted above).

How does the idea that status-seeking risk taking is a stable psychological trait fit with the finding that such individuals are "more numerous in undemocratic countries"? The authors themselves write later that this behaviour may depend on social/economic factors, hence it can well be that status-seeking is influenced by online hostility, too.

Trait “stability” means *rank-order* consistency: people keep the same relative position in a group even as everyone changes (Roberts & Del Vecchio 2000) While we lack direct empirical studies on SDRT, longitudinal data for aggression is illustrative here: children who are more aggressive than peers at 8 remain so decades later (Huesmann, Dubow & Boxer 2009) Absolute levels, however, move with context: early maltreatment elevates later violence (Lansford et al. 2007) income-inequality raises national violence rates (Wolf, Gray & Fazel 2014) and aggression peaks in late adolescence before declining (Nagin & Tremblay 1999). Hence individual trait stability can co-exist with mean-level cross-country differences. Kunst et al. 2017, for example, show that "the degree of structural inequality, social instability, and violence in different countries and US states is reflected in their populations’ minds in the form of support of group-based hegemony".

For the sake of brevity we summarise this in the following way in the manuscript:

> Another possibility is that there are more status-driven people in more unequal and undemocratic countries, i.e., political and economic inequalities encourage more people to adopt aggressive status-seeking strategies \cite{kunst2017preferences}. Note, that is consistent with the notion that status-seeking is a stable trait, since stability refers to rank-order persistence rather than fixed mean levels \cite{roberts2000rank}. (p10)

Additionally, we now explicitly acknowledge the Reviewer's point that "status-seeking is influenced by online hostility": "Some of our correlations may be inflated by individuals who are radicalized online and subsequently turn hostile offline and report elevated status-driven motivations." (p13)

Relatedly, here the authors don't provide a reference to prior research: "While our data cannot establish causality, theory and prior research suggest that status-driven risk-taking, as a stable trait, is more likely to drive online hostility than be shaped by it." As this sentence carries a strong assumption about the independence of certain behaviour from the environment, it would be important.

We deleted this sentence.

Furthermore, in the discussion the authors argue: "our findings offer an important corrective to conventional wisdom about the origins of hostility in online discussions on societal matters", which again is suggesting a causal order that I can't read in the data. In addition, I am not sure what conventional wisdom the authors are arguing against, at least in the scientific literature.

We deleted this sentence.

Same here: "Our findings, however, clearly show that online hostility is not just an accident or an artifact of well-meaning individuals' corruption by technology." It is very difficult to show these things and usually requires controlled- or natural experiments or some temporal dimension. The descriptive data is not a problem in itself, but it should not be matched with such strong statements.

We deleted this sentence.

Further down in the discussion section the authors say: "Our cross-cultural data challenge the overly pessimistic narrative about social media's impact on society that prevails in the West. Policy, journalistic and academic narratives in the U.S. and Europe often single out social media as uniquely responsible for fueling affective polarization, spreading fake news and conspiracy theories, and harming mental health." If the conclusion here would be that online hostility shows remarkable heterogeneity across political contexts, I would totally agree, but I feel it is a bit of a strawman to present the findings as "challenge overly pessimistic narratives" and implies that the data rules out some of those potential effects.

We deleted the entire paragraph with our admittedly speculative view that our results warrant a more optimistic view on SoMe's impact. Our revised Discussion stays closer to a neutral discussion of our empirical results.

In addition I still find it very hard to follow the Hypothesis in connection to the preregistration as the numbering has completely changed, maybe a table in the methods section that states which have been confirmed and which have not would help a lot.

We added the following table to help the readers orient themselves.

Table B.1: Overview of our seven hypotheses. Note that we changed the order and numbering, but not the content of our hypotheses compared to the pre-registration.

Original Number	Updated Number	Descriptive Prediction	Concl.
H1	H4	People who report more (vs less) offline hostility also report more online hostility. Specifically, we predict that the standardized beta will be larger than 0.5.	Accept
H2	H5	People higher (vs lower) on status-driven risk taking are more hostile online. Specifically, we predict that the standardized beta will be larger than 0.1.	Accept
H3	H6	People who feel more (vs less) meaninglessness are more hostile online. Specifically, we predict that the standardized beta will be larger than 0.1.	Reject
H4	H7	The association between online hostility and status-driven risk taking is larger among individuals who feel more (vs less) meaninglessness. Specifically, we predict that the standardized beta (of the interaction term) will be larger than 0.	Reject
H5	H2	Countries with more (vs less) economic inequality have more online hostility.	Accept
H6	H3	Countries with more (vs less) poverty have more online hostility.	Accept
H7	H1	Countries with less (vs more) liberal democracy have more online hostility.	Accept

Furthermore, in the follow-up study 4 I don't see why the effects of narrative only go in one direction and why this would mean that the cross-country differences reported here are a lower bound, please expand.

We clarified our argument in the following way:

> While our data do not allow us to precisely estimate the resulting bias, they do indicate its likely direction. Specifically, as shown in Online Appendix L, more democratic countries---where self-reported victimization is lower---tend to have *stronger* narratives in circulation portraying social media as a source of turmoil. Insofar as turmoil narratives inflate self-reported victimhood, perceptions in more democratic countries are likely upwardly biased, exaggerating hostility victimization. In contrast, less democratic countries---despite reporting higher levels of victimization---are less permeated by turmoil narratives, suggesting their self-reported hostility is less inflated. We would expect more bias towards hostility over-reporting in countries with little hostility, and less bias in countries with more hostility. Following this logic, our estimates of cross-country differences in victimization are likely conservative; the true disparities may be even larger than observed. (p58)

As I said, I would appreciate a paper that reports the differences of online hostility across countries, the individual-level correlates and discuss potential mechanisms behind that. I am a

huge supporter of descriptive work, but in the current form the narrative around it is too strong and does not discuss alternative explanations.

Thank you! We added the following paragraph on alternative explanations:

> What *causes* the observed differences in hostility across countries and individuals remains an open question. Our own theorizing favors a mechanism wherein offline differences in personality and socioeconomic environment shape online behavior. But our observational data cannot rule out or quantify the opposite mechanism. It remains conceivable that online hostility also shapes the offline world, influencing respondents' (self-reported) personality and their broader social and political environment. Some of our correlations may be inflated by individuals who are radicalized online and subsequently turn hostile offline and report elevated status-driven motivations. It is plausible that hostile online political discussions undermine social forces pushing for more democracy and equality. Future research should examine longitudinal data on local inequalities or external shocks to individual status motivations to quantify their causal impact on online hostility. (p12-13)

Reviewer #2 (Remarks on code availability):

I only had a brief look at the code and it seems well documented and I could follow the code behind the main figures. The data will be a valuable resource for the community.

Thank you!

Reviewer #3 (Remarks to the Author):

The authors have done a thorough job responding to comments from the reviewers, as well as from the editors. It is especially good that they've made it even more clear that their aim is to be descriptive, which is welcome in this regard.

I agree with the authors that the hostility measures in this case cannot be supplemented with scraped data as observed behavioral measures from platforms. Furthermore, I support the authors' contention that the self-reported measures the study employs purposively measure whether people "feel" offended or affected by hostile messages, which is most in line with the study's intent to capture perceptions of group-based victimization, which is of most concern in the democratic sense. This also captures then hostility from those with status, in unequal contexts, to those seeking equal status.

I'm glad to see the authors address the normative aspect briefly, through the descriptives from the MRP analysis that they provided in the response letter (that shows young men with low

education and minorities report more hostilities) is a good example to highlight the point they make in the text.

We are grateful for the supportive comments!

Bibliography

Roberts, B. W., & Del Vecchio, W. F. (2000). *The rank-order consistency of personality traits from childhood to old age: A quantitative review of longitudinal studies. Psychological Bulletin, 126*, 3-25.

Huesmann, L. R., Dubow, E. F., & Boxer, P. (2009). *Continuity of aggression from childhood to early adulthood as a predictor of life outcomes. Aggressive Behavior, 35*, 136-149.

Lansford, J. E., Miller-Johnson, S., Berlin, L. J., Dodge, K. A., Bates, J. E., & Pettit, G. S. (2007). *Early physical abuse and later violent delinquency: A prospective longitudinal study. Child Maltreatment, 12*, 233-245.

Wolf, A., Gray, R., & Fazel, S. (2014). *Violence as a public health problem: An ecological study of 169 countries. Social Science & Medicine, 104*, 220-227.

Nagin, D. S., & Tremblay, R. E. (1999). *Trajectories of boys' physical aggression, opposition, and hyperactivity on the path to physically violent and non-violent juvenile delinquency. Child Development, 70*, 1181-1196.

Kunst, J. R., Fischer, R., Sidanius, J., & Thomsen, L. (2017). *Preferences for group dominance track and mediate the effects of macro-level social inequality and violence across societies. Proceedings of the National Academy of Sciences, 114*, 5407-5412.
